# Supplementary material for: Relationship between Structure and Antibacterial Activity of α-Aminophosphonate Derivatives Obtained via Lipase-Catalyzed Kabachnik−Fields Reaction
Source: Materials (Basel). 2022 May 27;15(11):3846. doi: 10.3390/ma15113846 (PMC9182137; doi:10.3390/ma15113846)
Supplement: Supplementary file 1 [file materials-15-03846-s001.zip › materials-1672088-supplementary.pdf]

materials

MDPI

Supplementary Materials

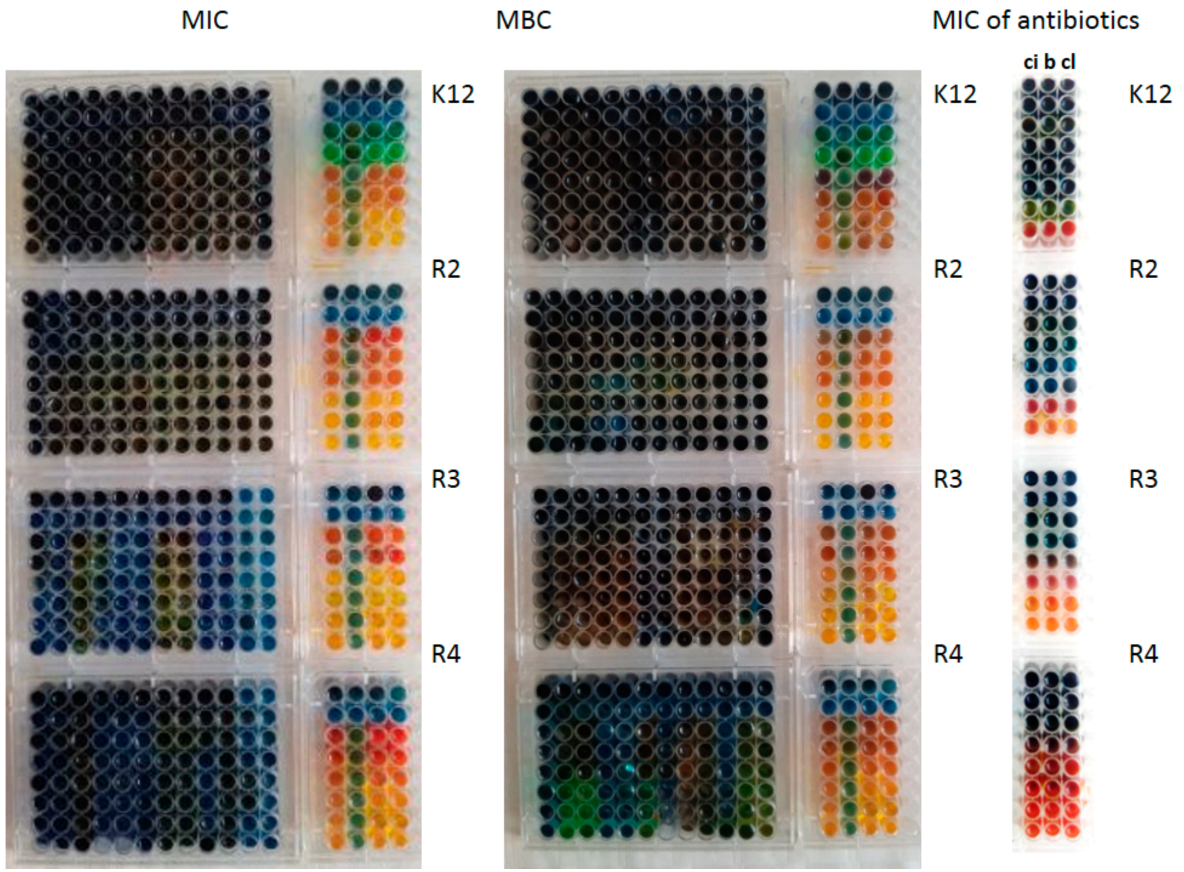

Figure S1. Examples of MIC and MBC on microplates with different concentration of studied compounds (μg/mL⁻¹). Resazurin was added as an indicator of microbial growth with K12, R2, R3, and R4 strains with tested 16 compounds, as described in Table 2. Additionally, examples of MIC with different strains K12, R2, R3, and R4 of studied antibiotics with ciprofloxacin (ci), bleomycin (b), and cloxacillin (cl) in (μg/mL⁻¹).

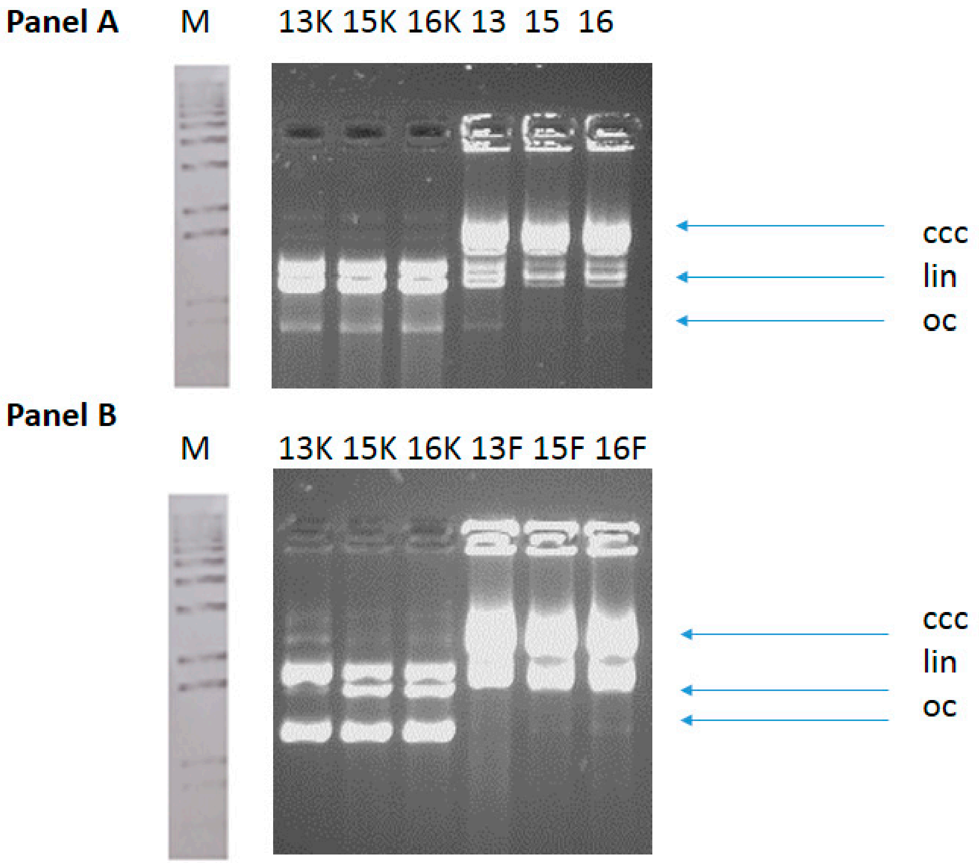

FigureS2. An example of an agarose gel electrophoresis separation of isolated plasmids DNA on R4 strains modified with selected coumarin derivatives (Panel A) from 3 selected compounds, as shown in Figure 3, and digested with repair Fpg protein (Panel B). M = marker.

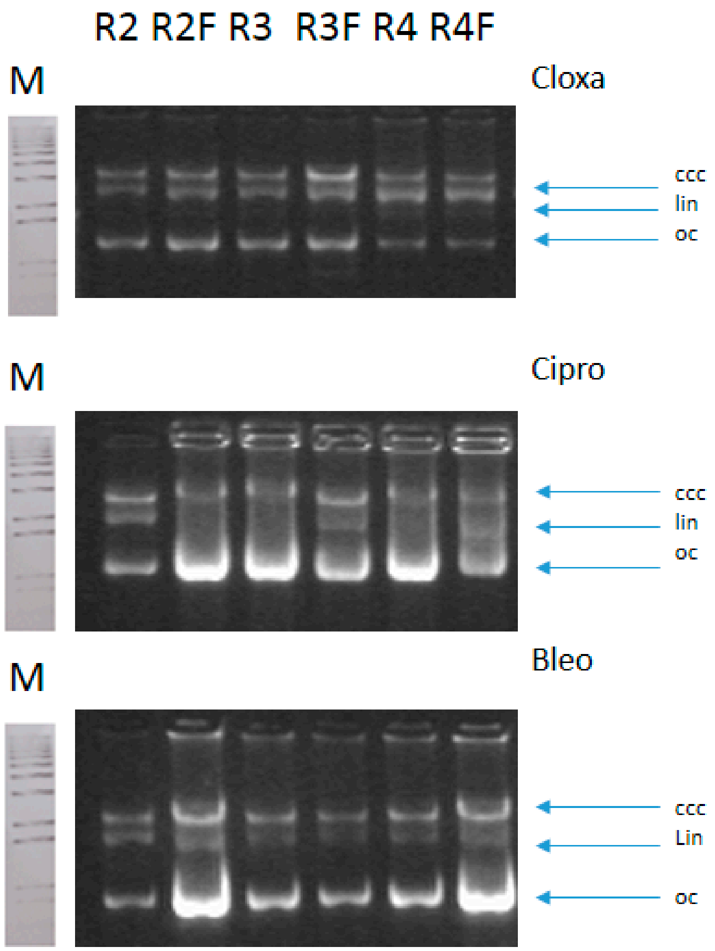

Figure S3. Example of an agarose gel electrophoresis separation of isolated plasmids DNA from R2-R4 strains modified with antibiotics: bleomycin, ciprofloxacin, and cloxacillin digested (or not) with repair enzymes Fpg. M = marker.

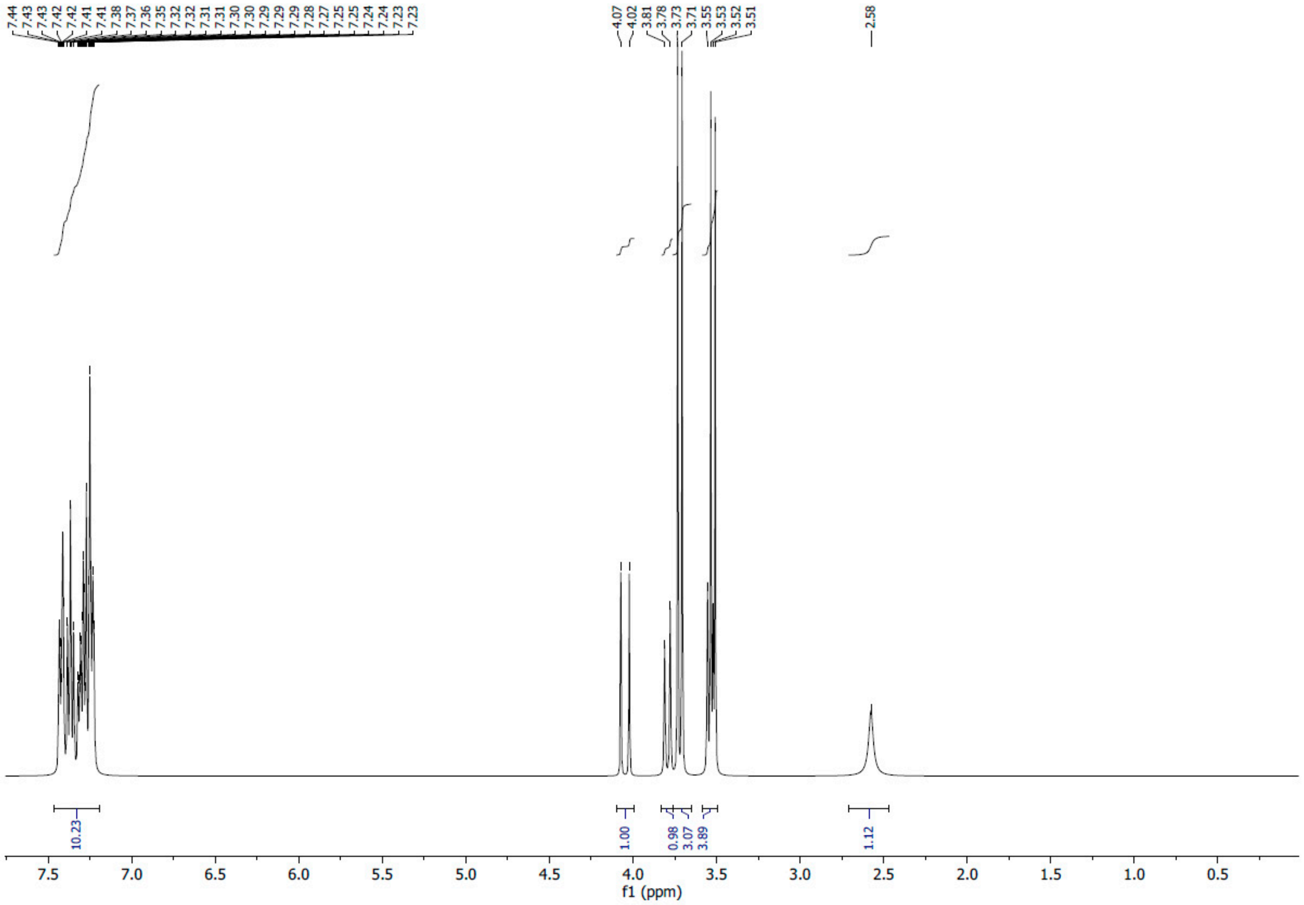

Figure S4. ^{1}HNMR (400 MHz, $CDCl_3$) spectra of compound **1**

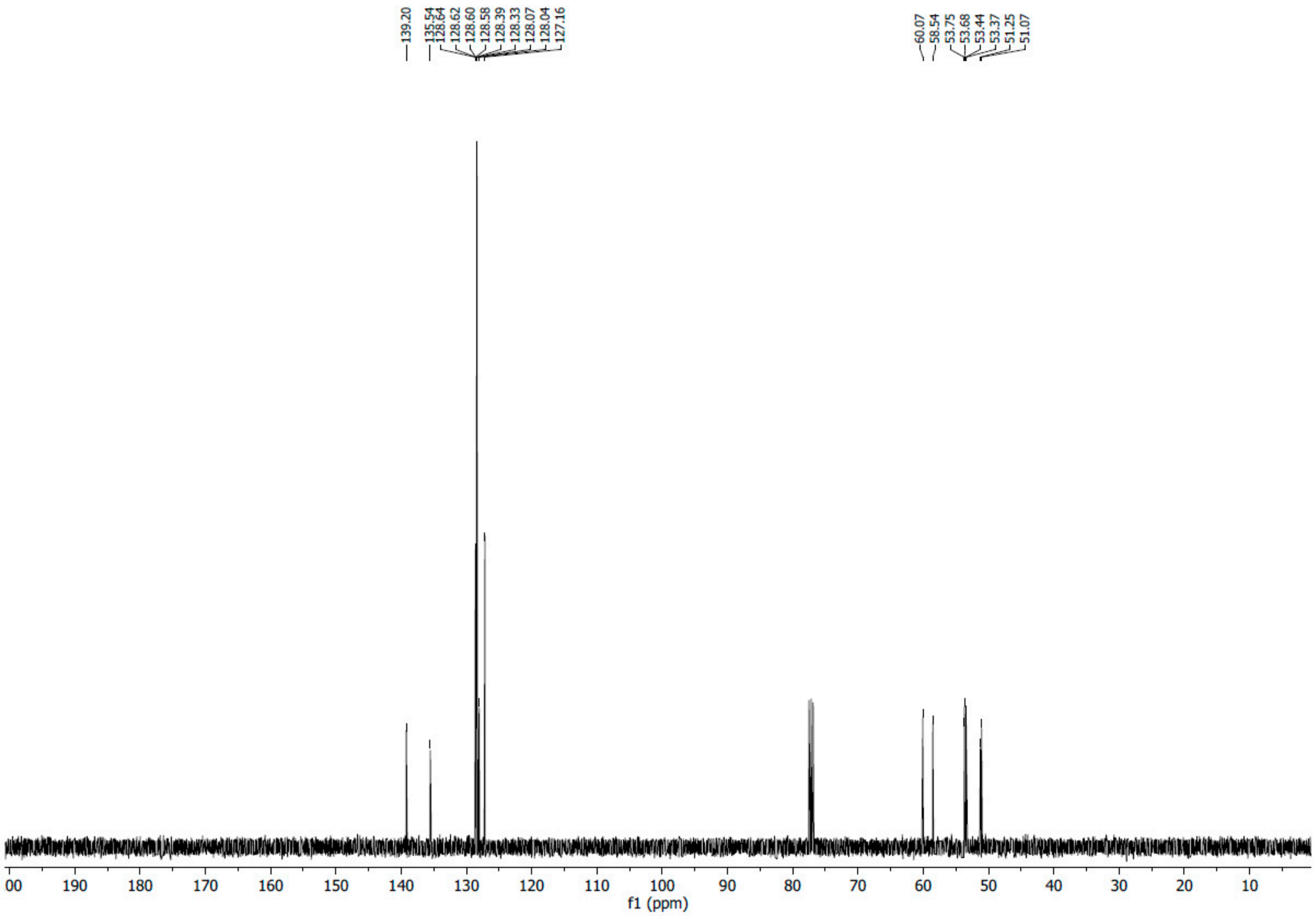

Figure S5. ^{13}CNMR (100 MHz, $CDCl_3$) spectra of compound **1**

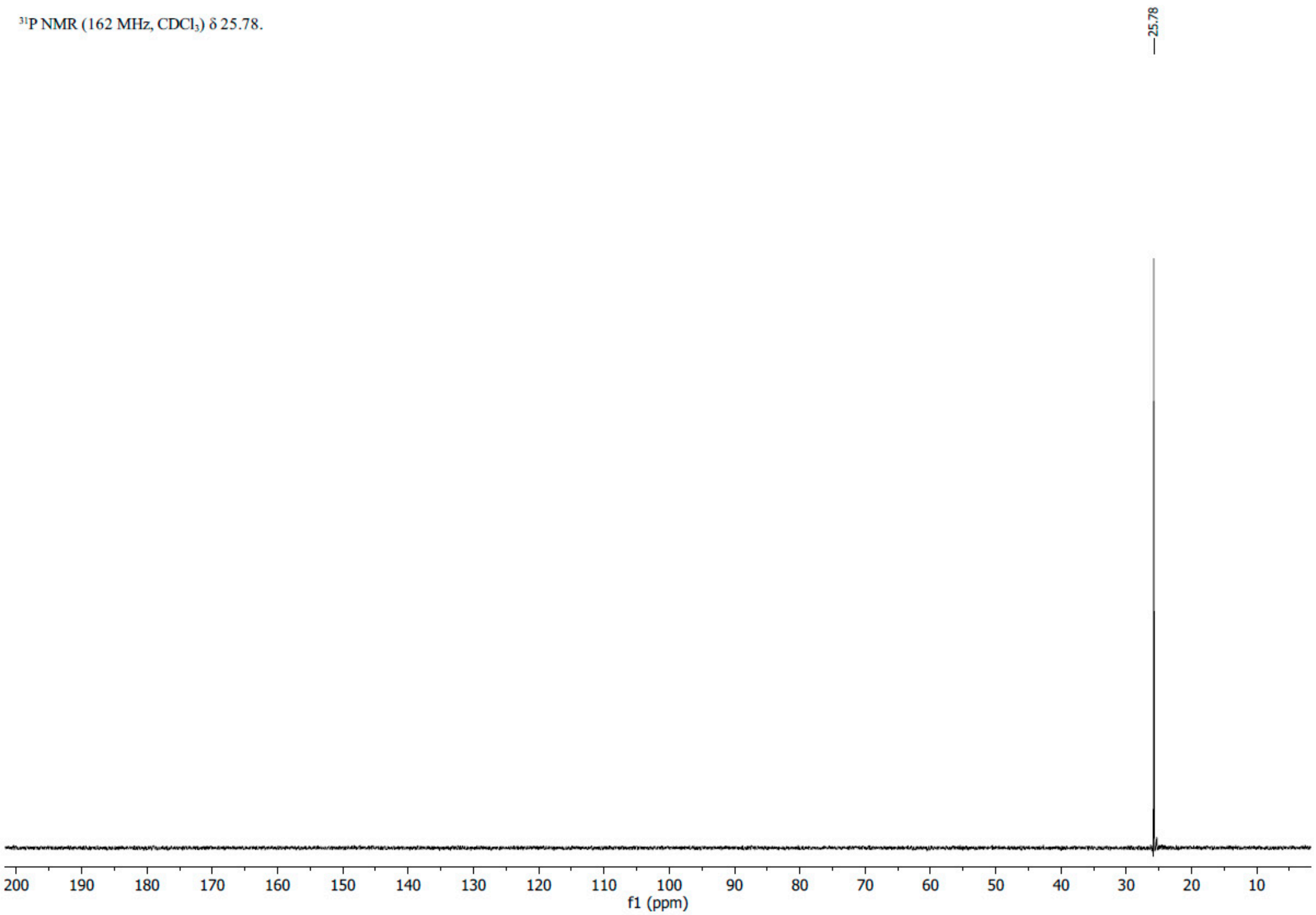

Figure S6. ^{31}PNMR (162 MHz, $CDCl_3$) spectra of compound **1**

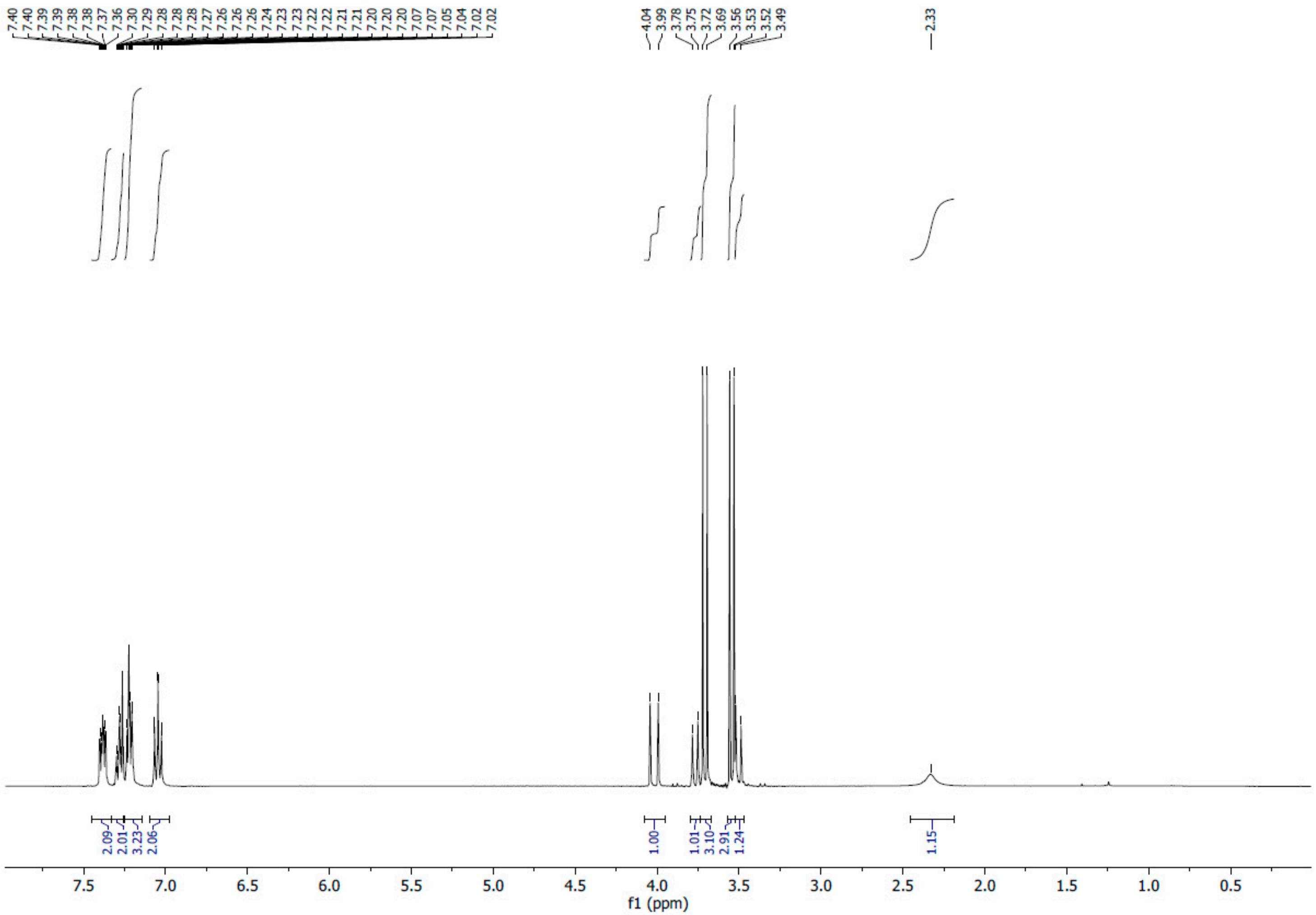

Figure S7. ^{1}HNMR (400 MHz, $CDCl_3$) spectra of compound **2**

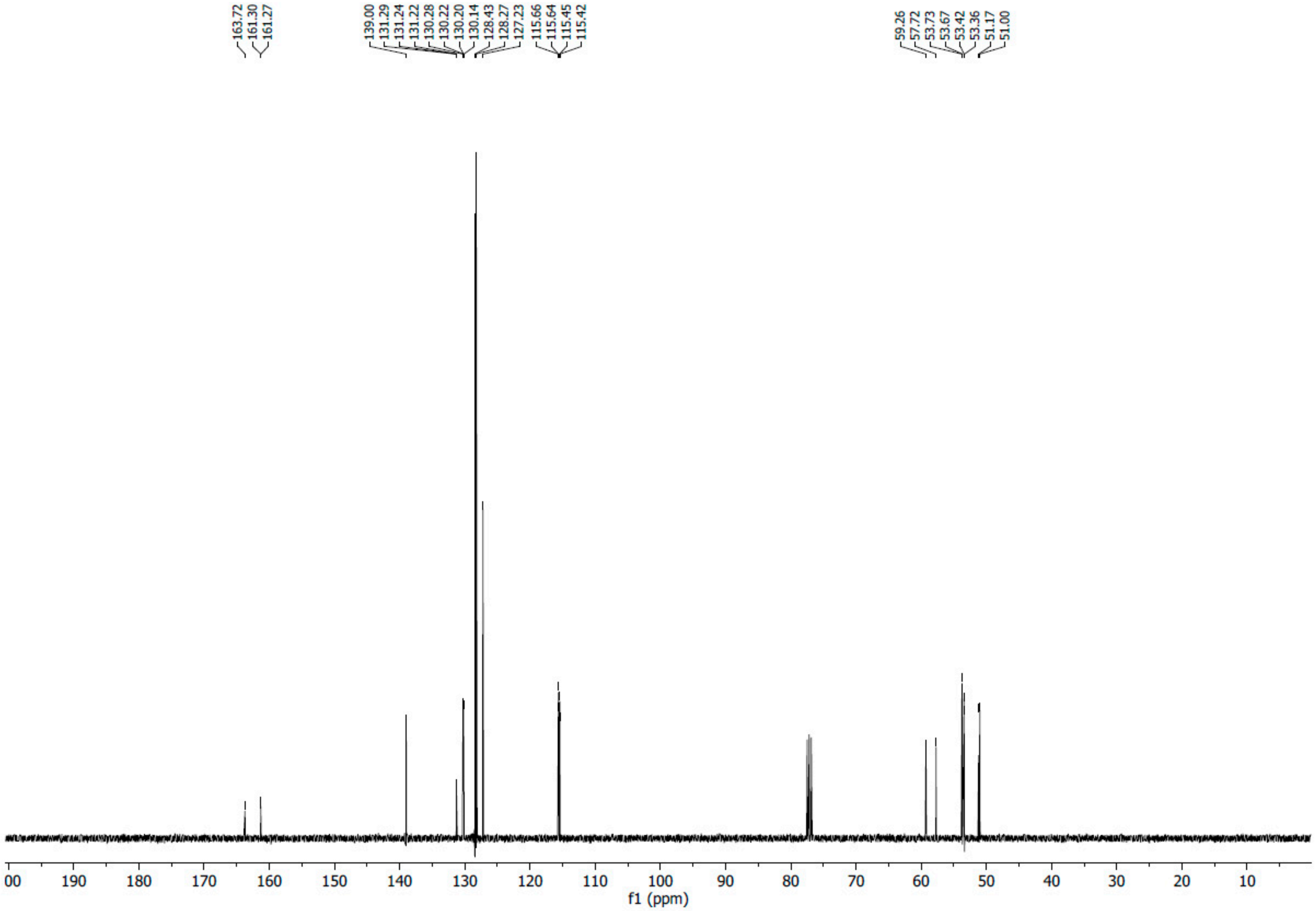

Figure S8. ^{13}CNMR (100 MHz, $CDCl_3$) spectra of compound **2**

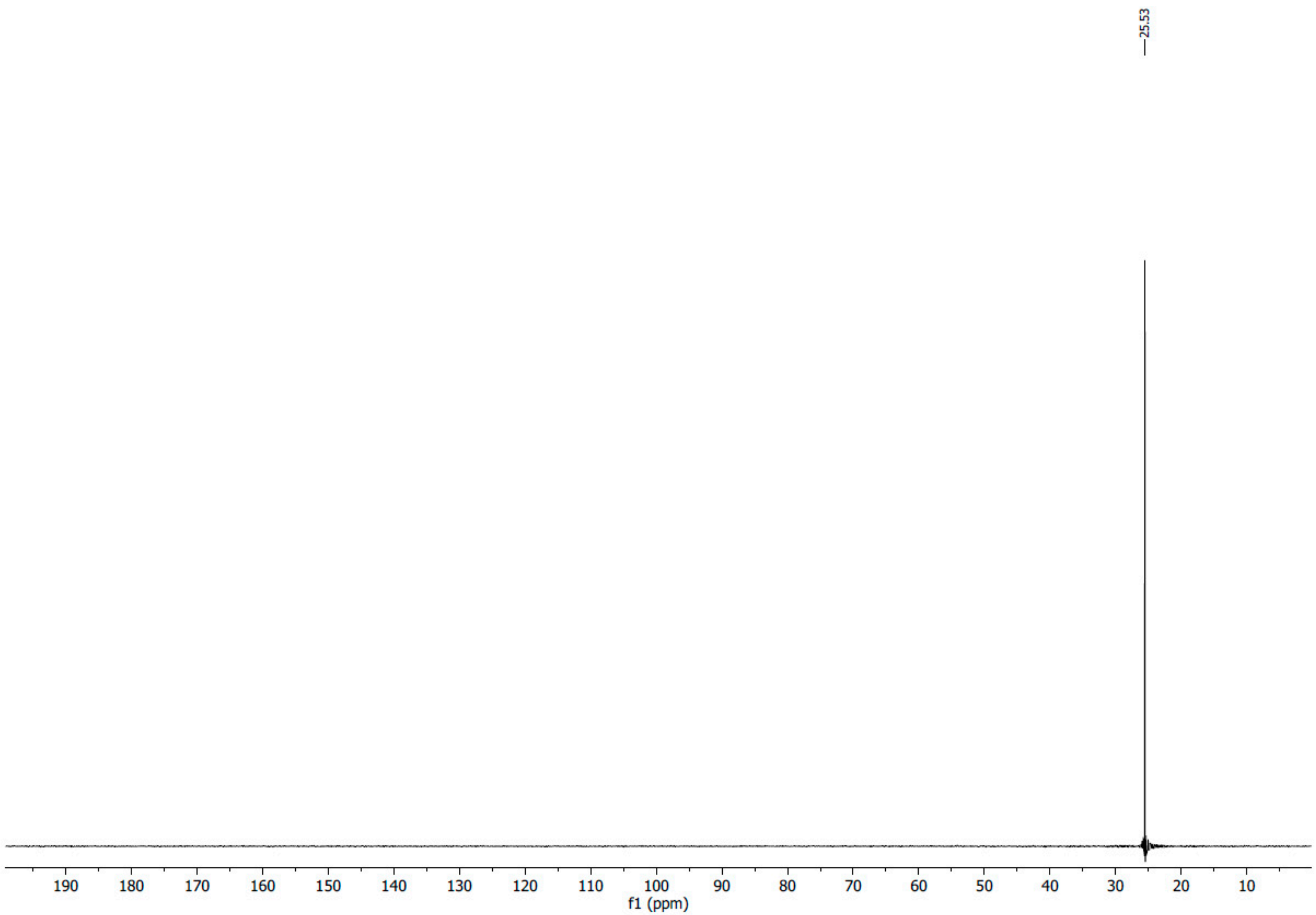

Figure S9. ^{31}PNMR (162 MHz, $CDCl_3$) spectra of compound **2**

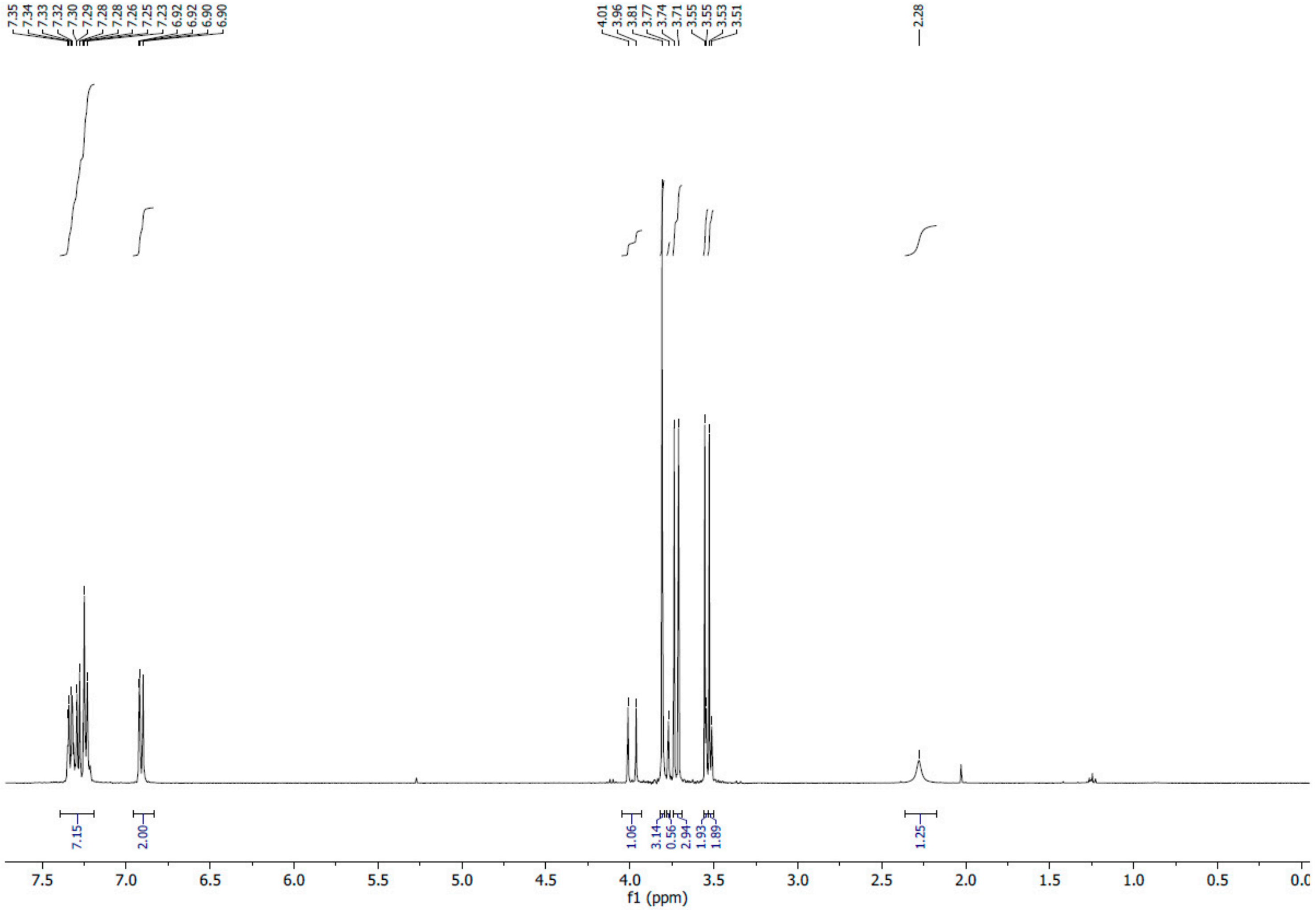

Figure S10. ^{1}HNMR (400 MHz, $CDCl_3$) spectra of compound **3**

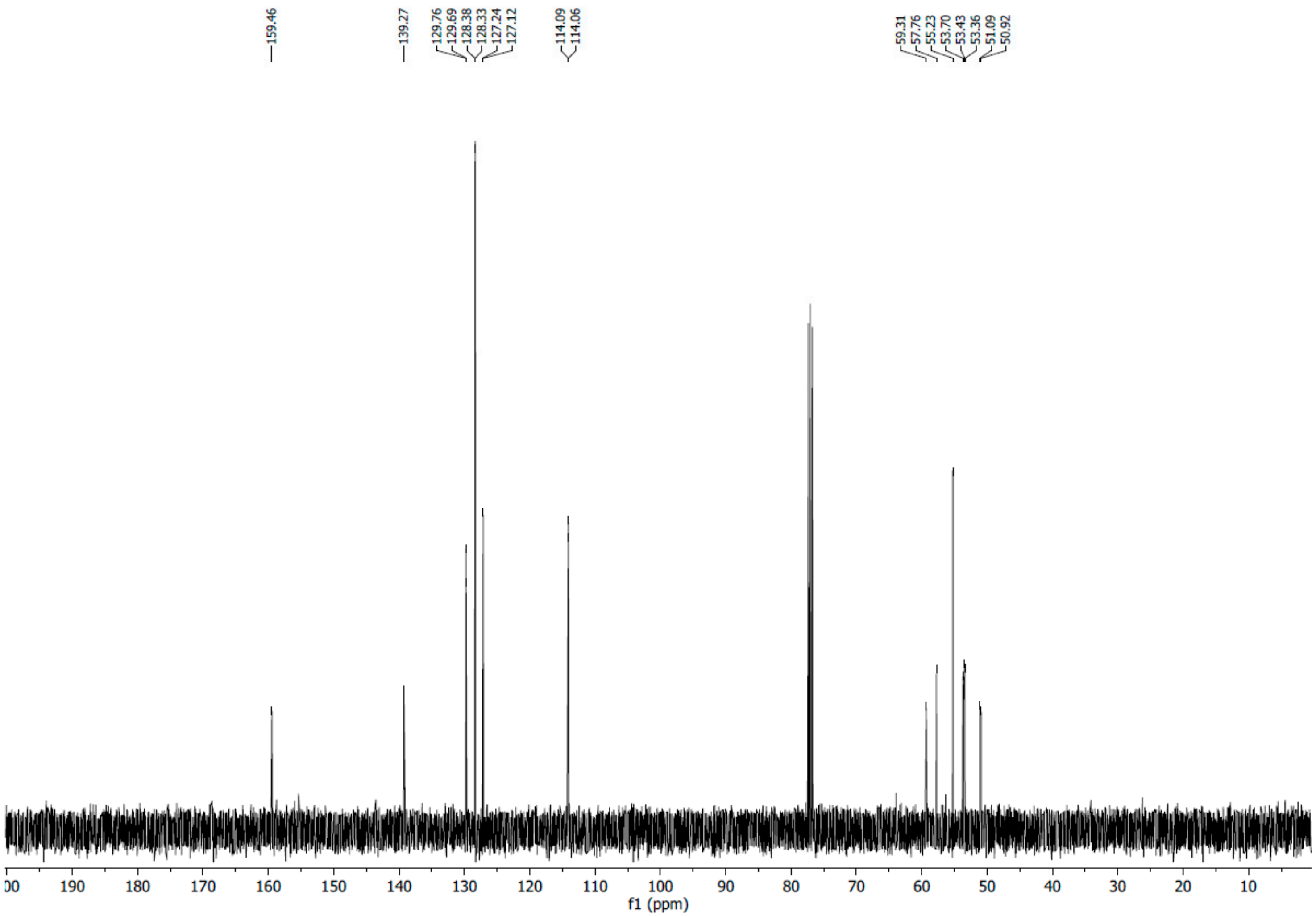

Figure S11. ^{13}CNMR (100 MHz, $CDCl_3$) spectra of compound **3**

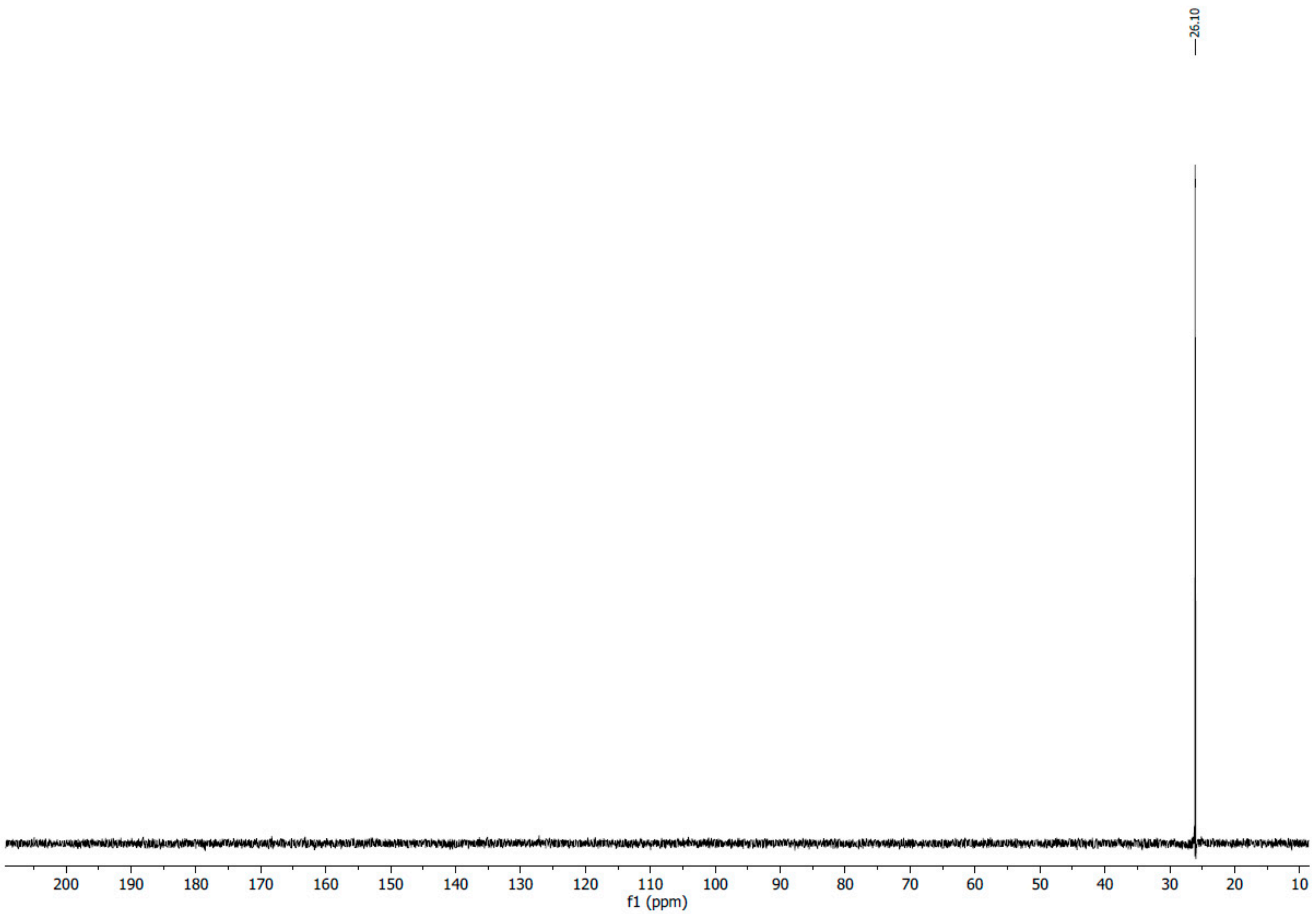

Figure S12. ^{31}PNMR (162 MHz, $CDCl_3$) spectra of compound **3**

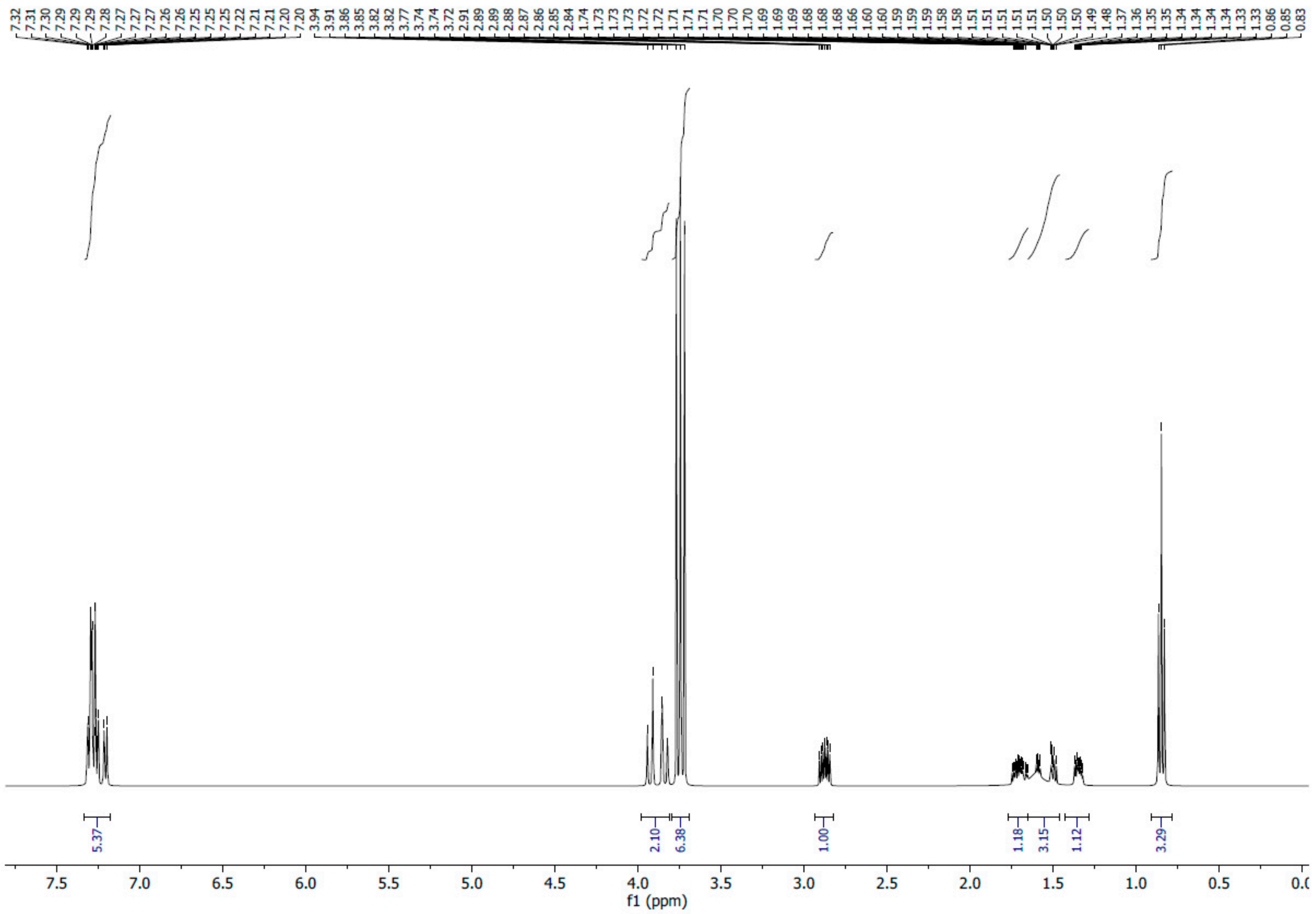

Figure S13. ^{1}HNMR (400 MHz, $CDCl_3$) spectra of compound **4**

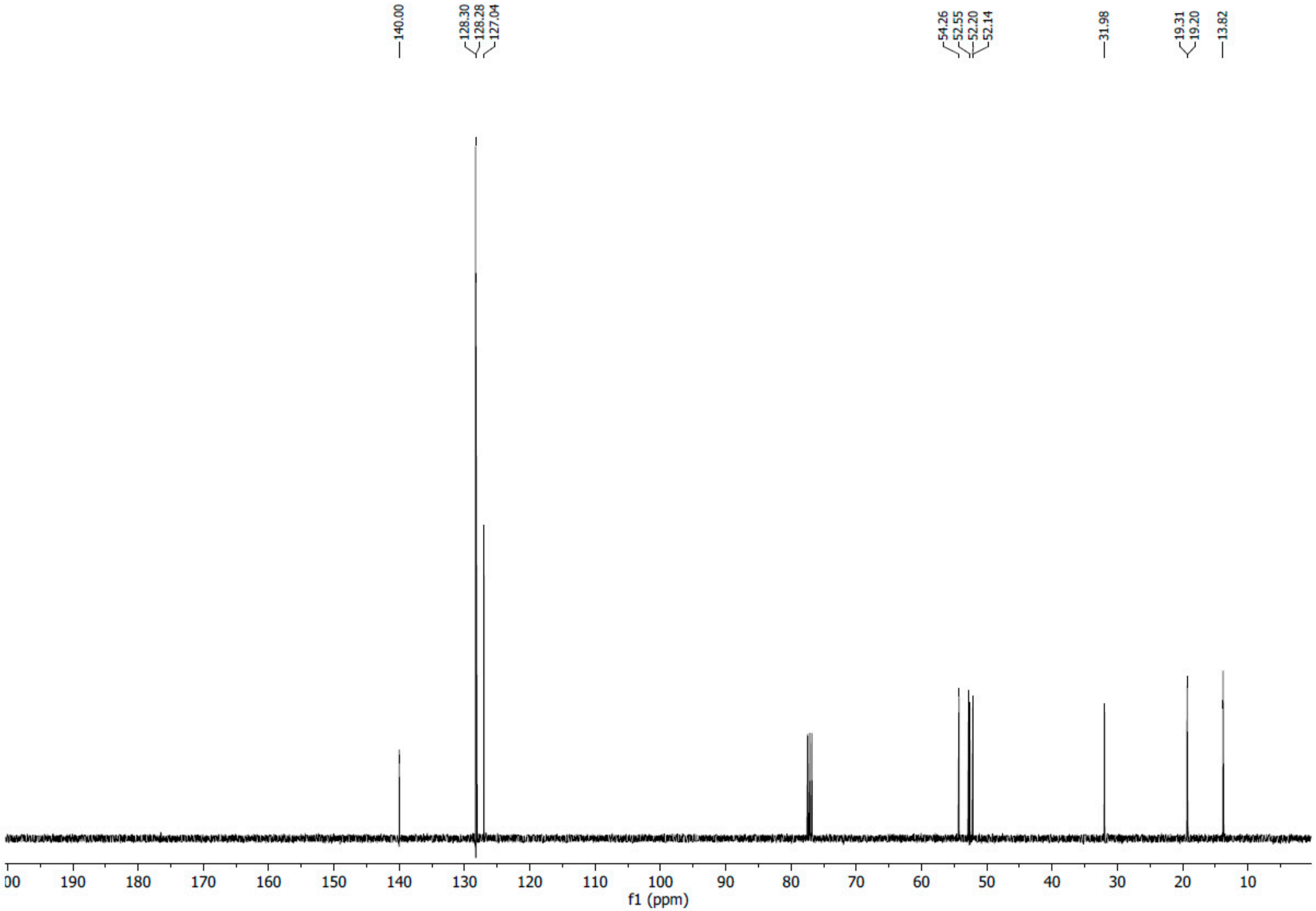

Figure S14. ^{13}CNMR (100 MHz, $CDCl_3$) spectra of compound **4**

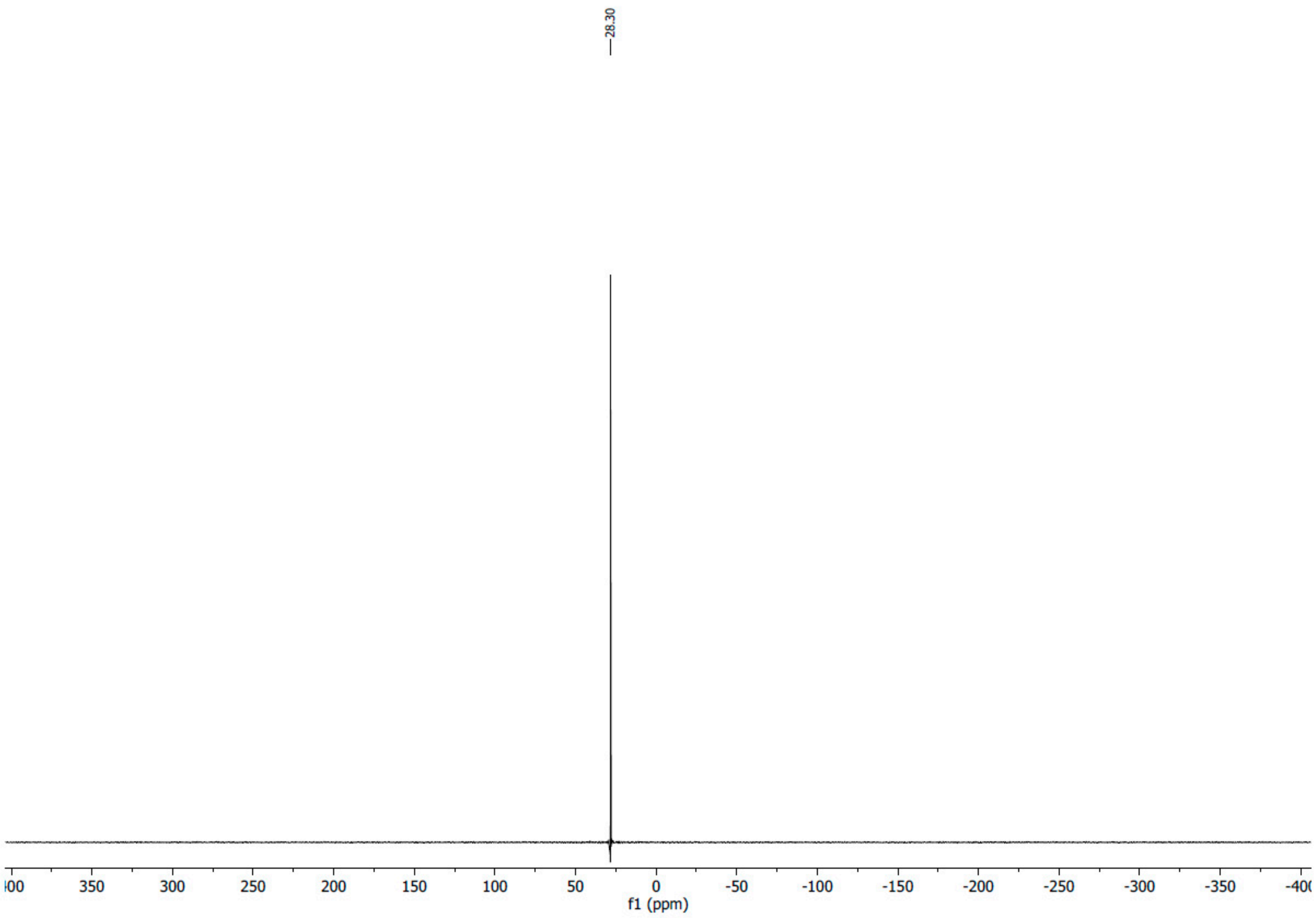

Figure S15. ^{31}PNMR (162 MHz, $CDCl_3$) spectra of compound **4**

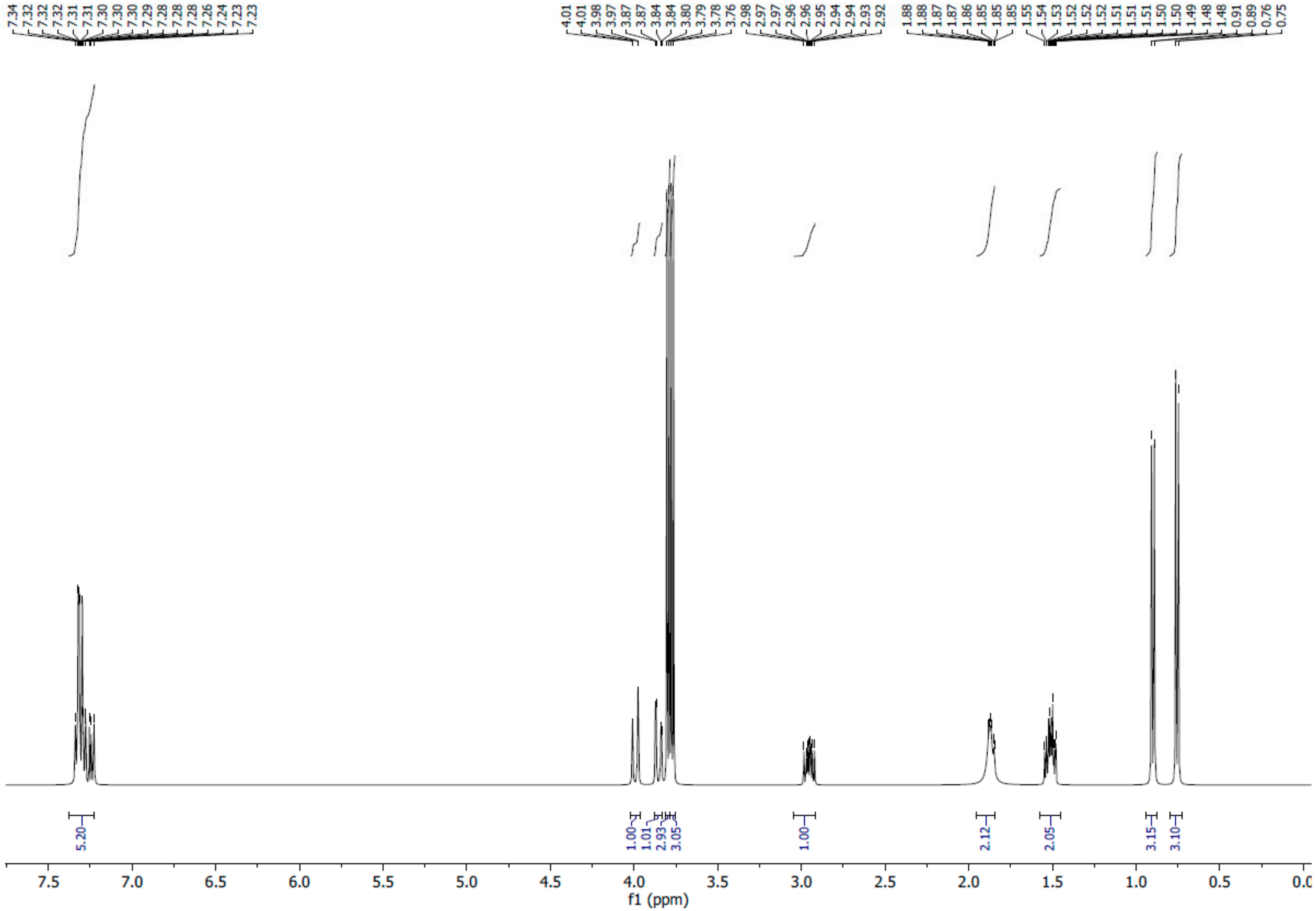

Figure S16. ^{1}HNMR (400 MHz, $CDCl_3$) spectra of compound **5**

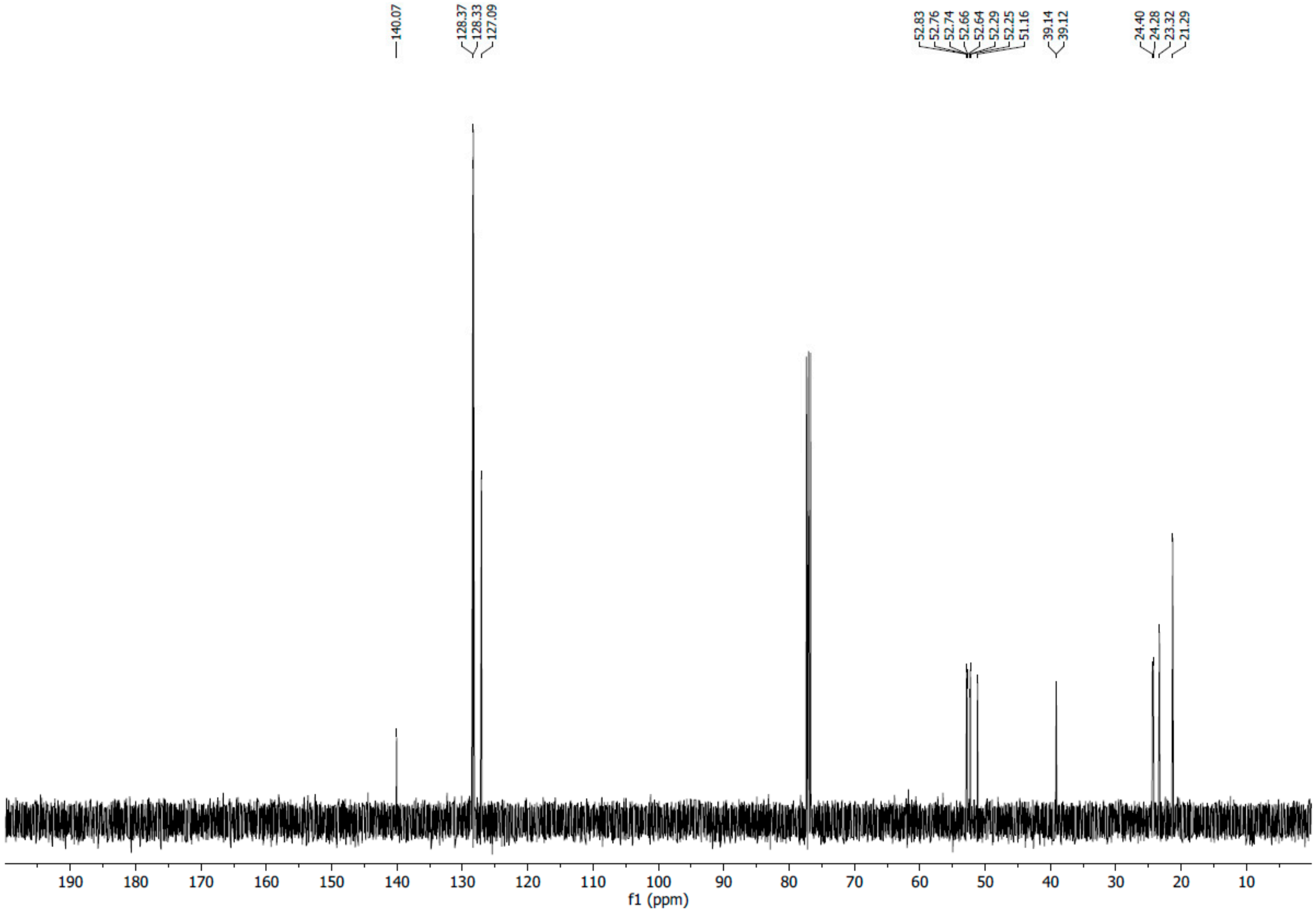

Figure S17. ^{13}CNMR (100 MHz, $CDCl_3$) spectra of compound **5**

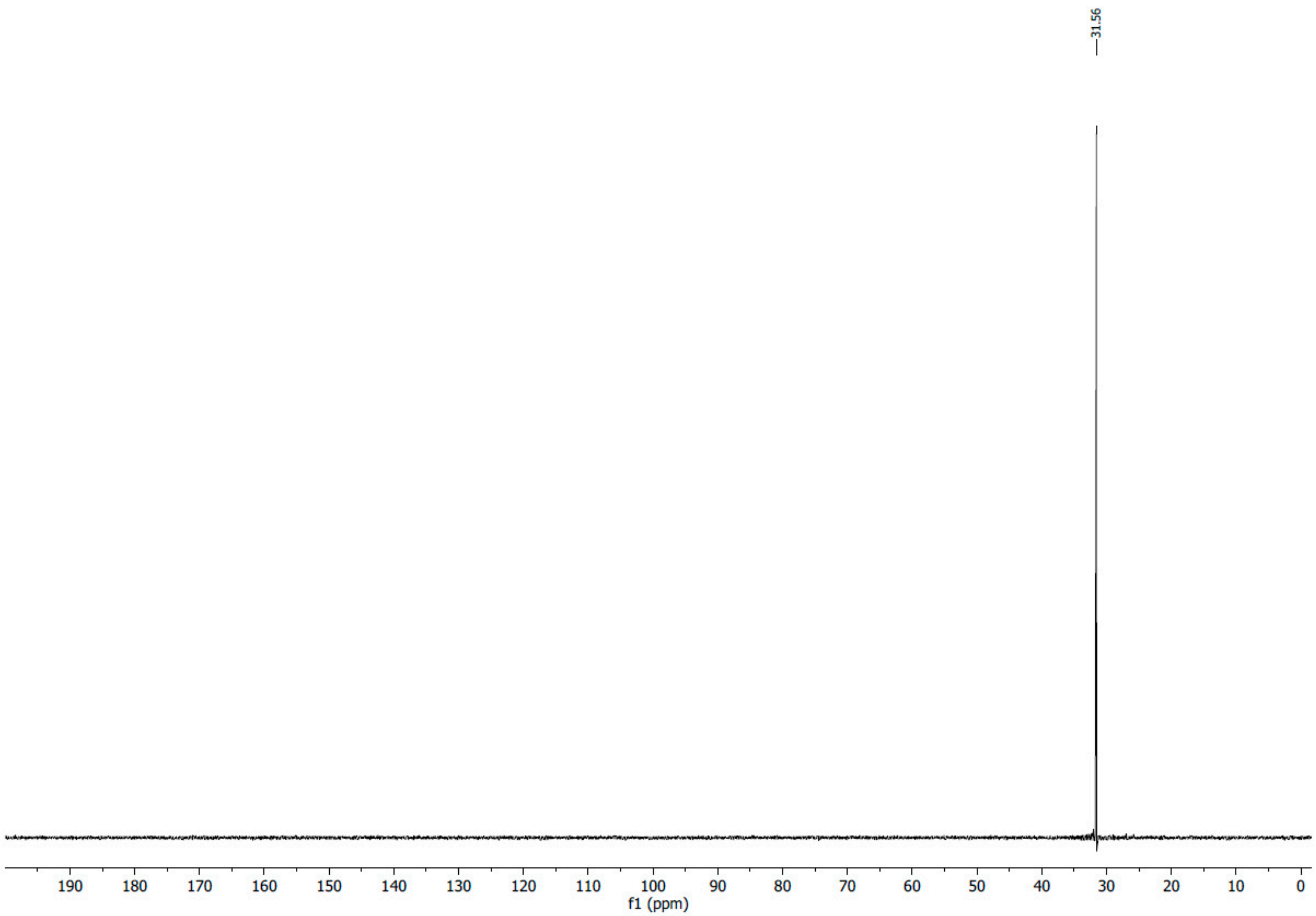

Figure S18. ^{31}PNMR (162 MHz, $CDCl_3$) spectra of compound **5**

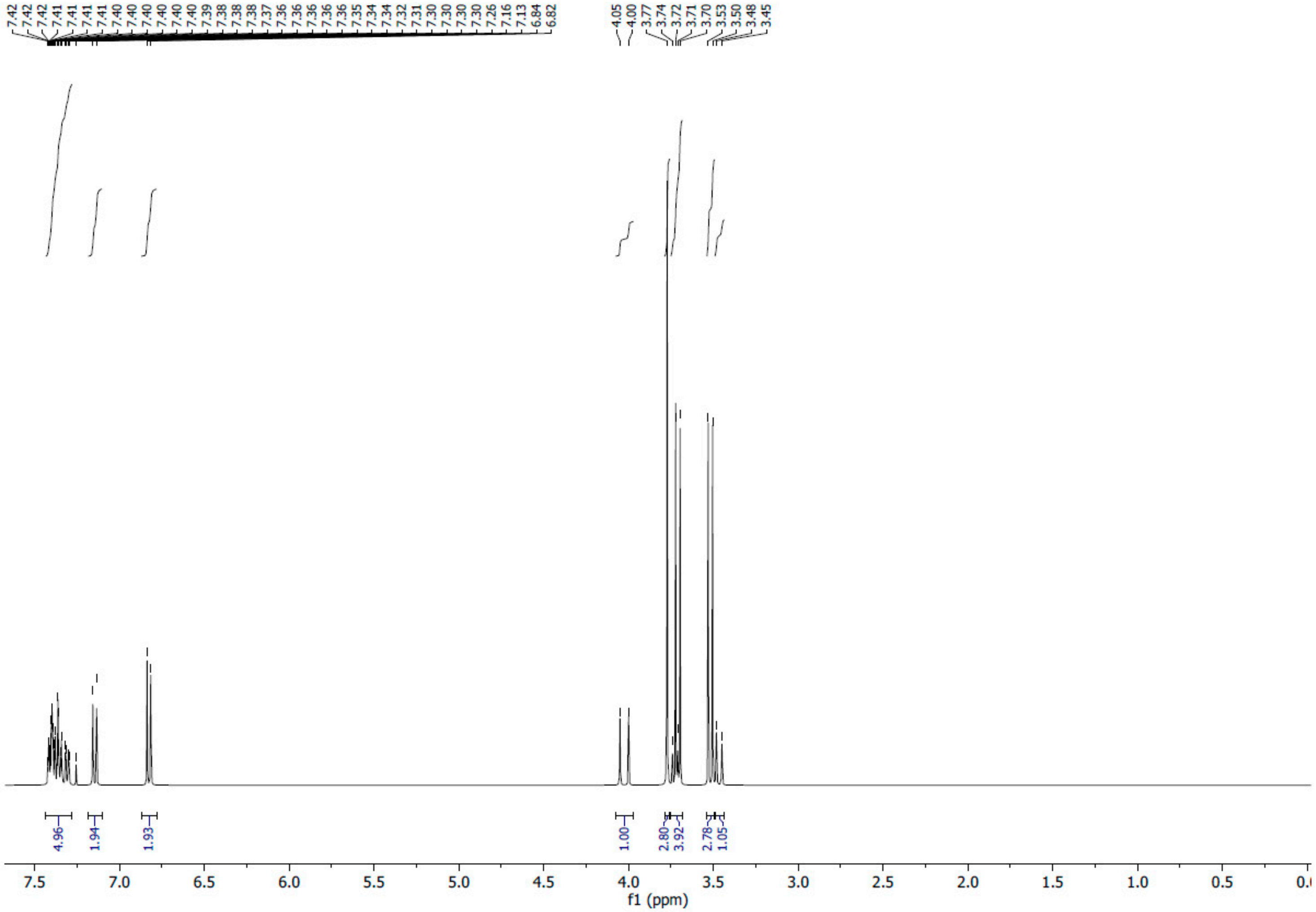

Figure S19. ^{1}HNMR (400 MHz, $CDCl_3$) spectra of compound **6**

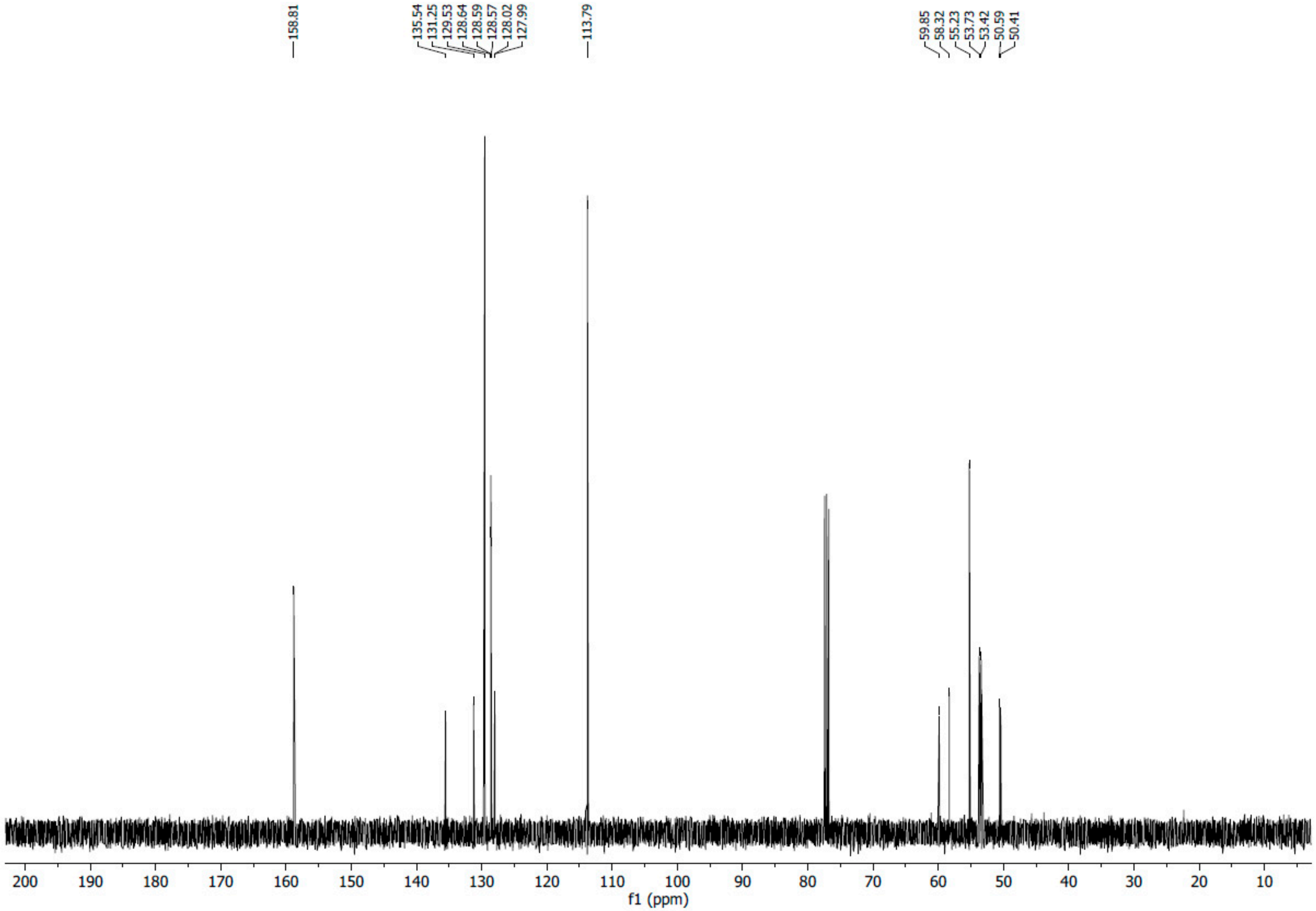

Figure S20. ^{13}CNMR (100 MHz, $CDCl_3$) spectra of compound **6**

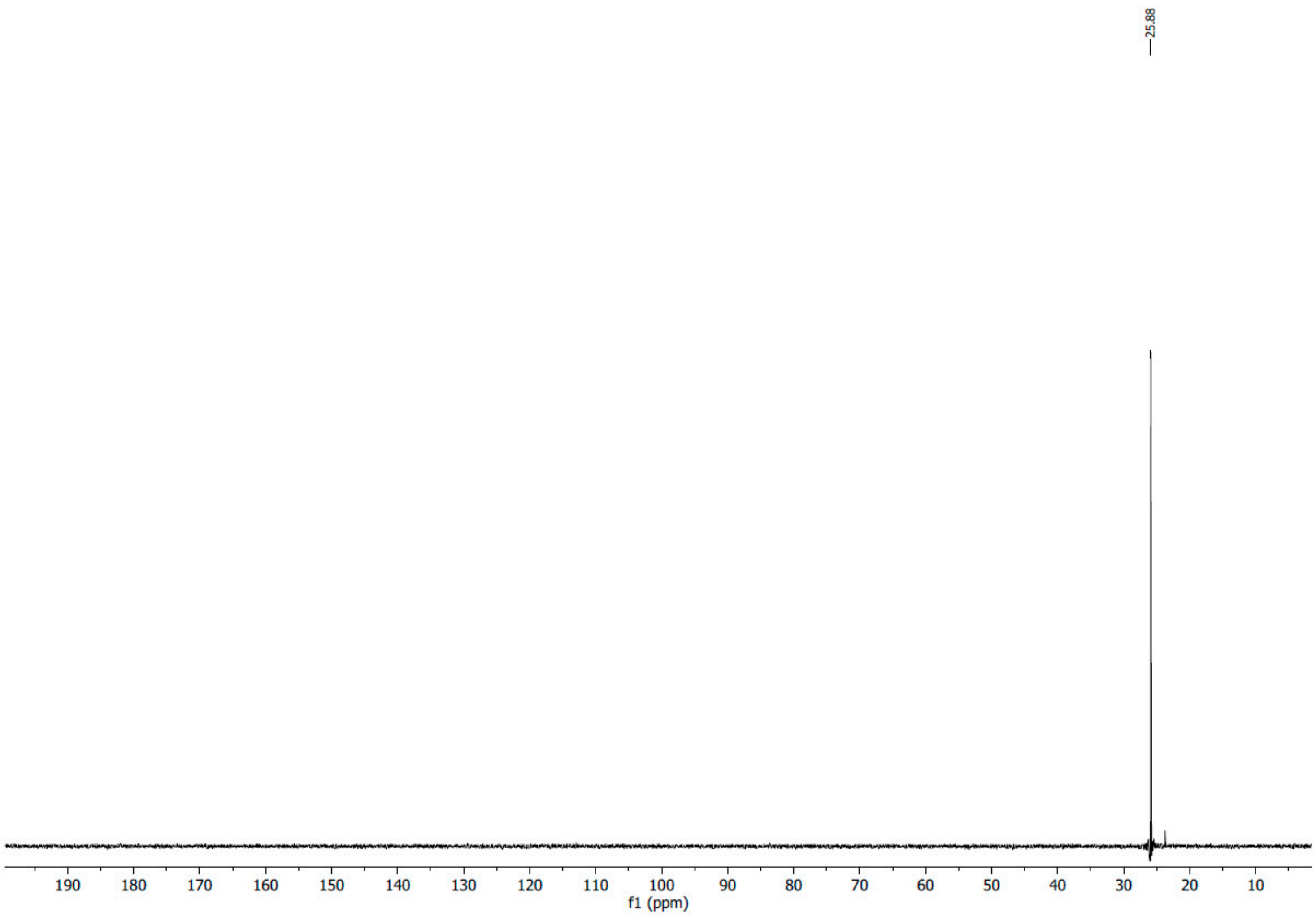

Figure S21. ^{31}PNMR (162 MHz, $CDCl_3$) spectra of compound **6**

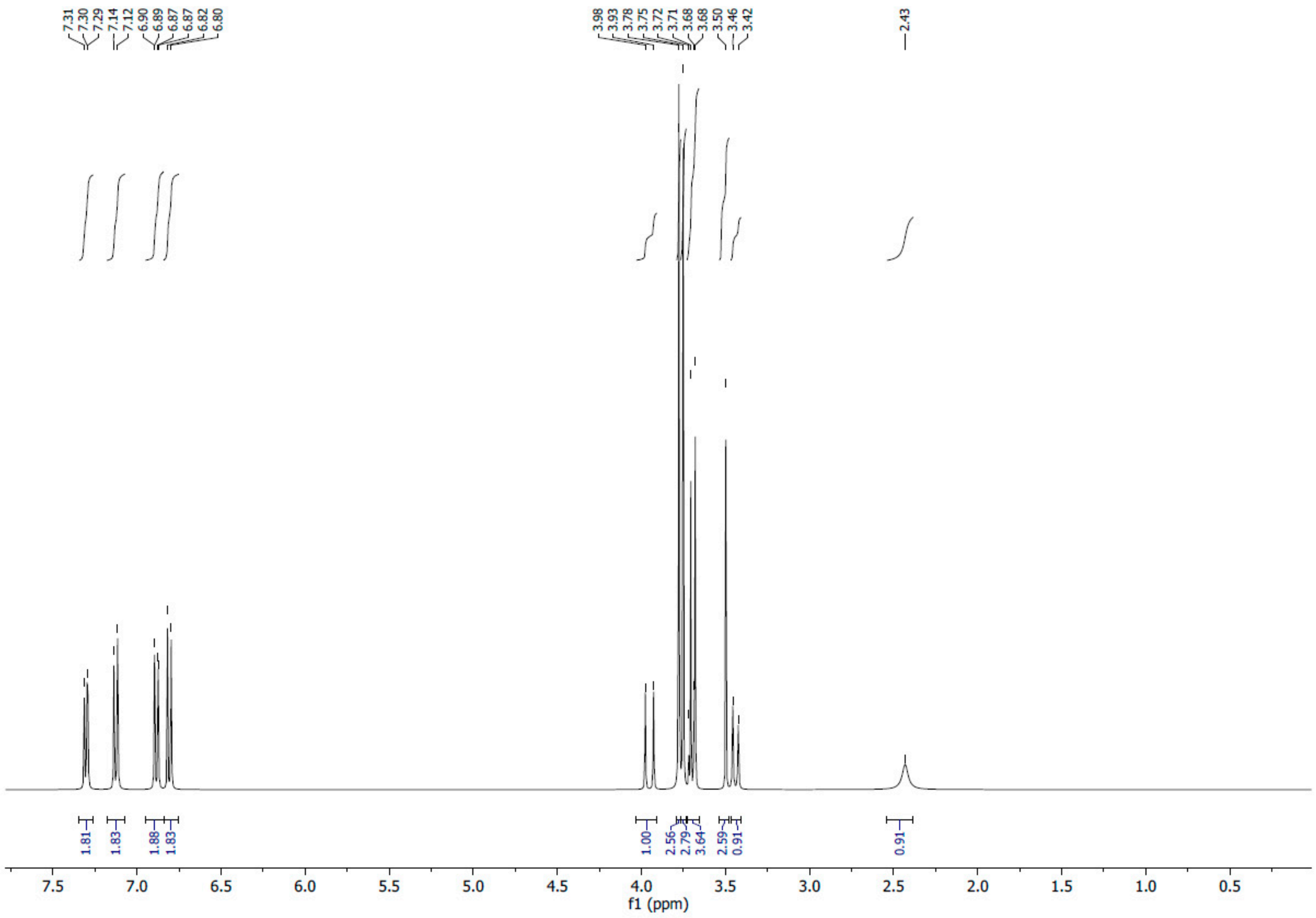

Figure S22. ^{1}HNMR (400 MHz, $CDCl_3$) spectra of compound **7**

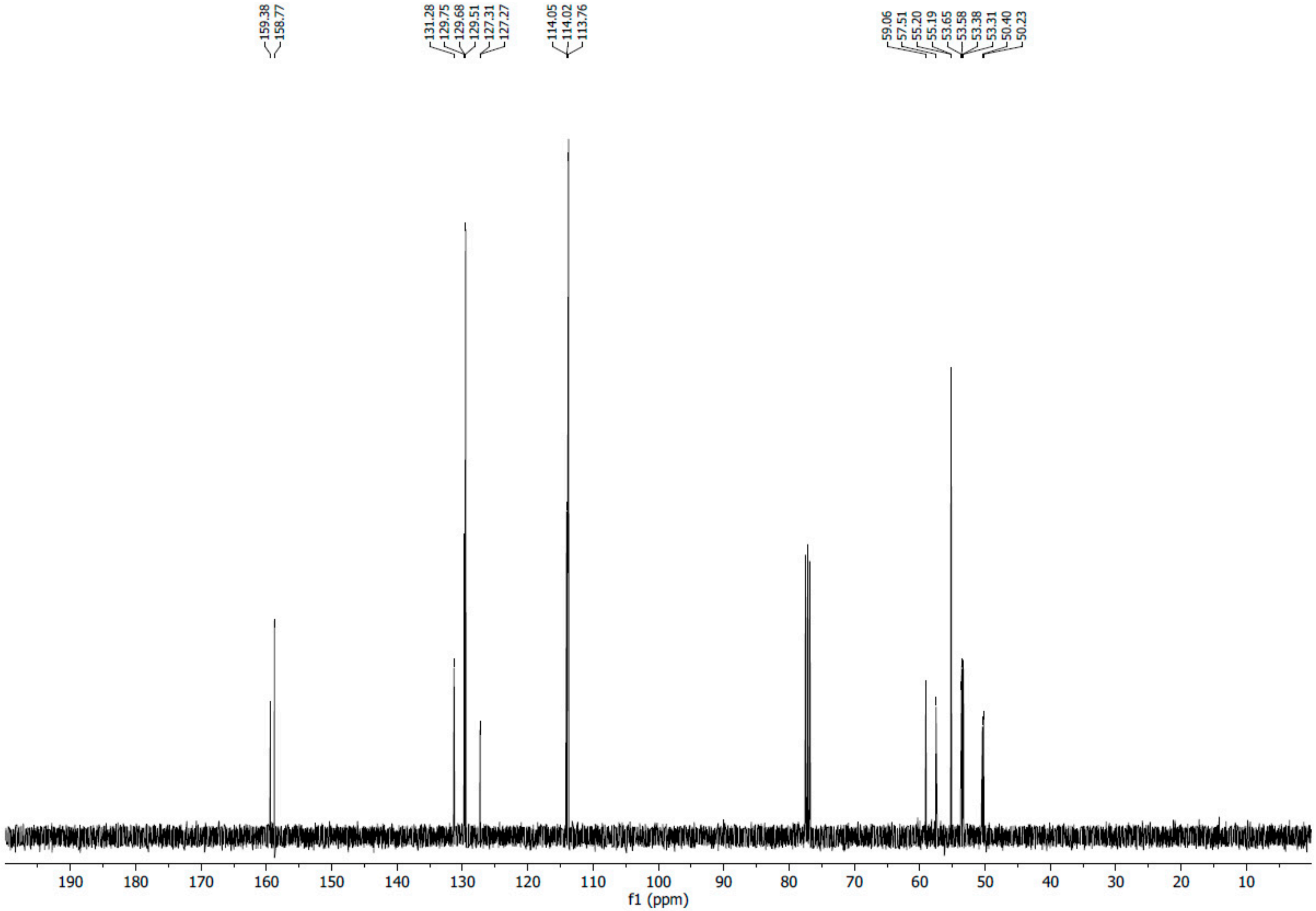

Figure S23. ^{13}CNMR (100 MHz, $CDCl_3$) spectra of compound **7**

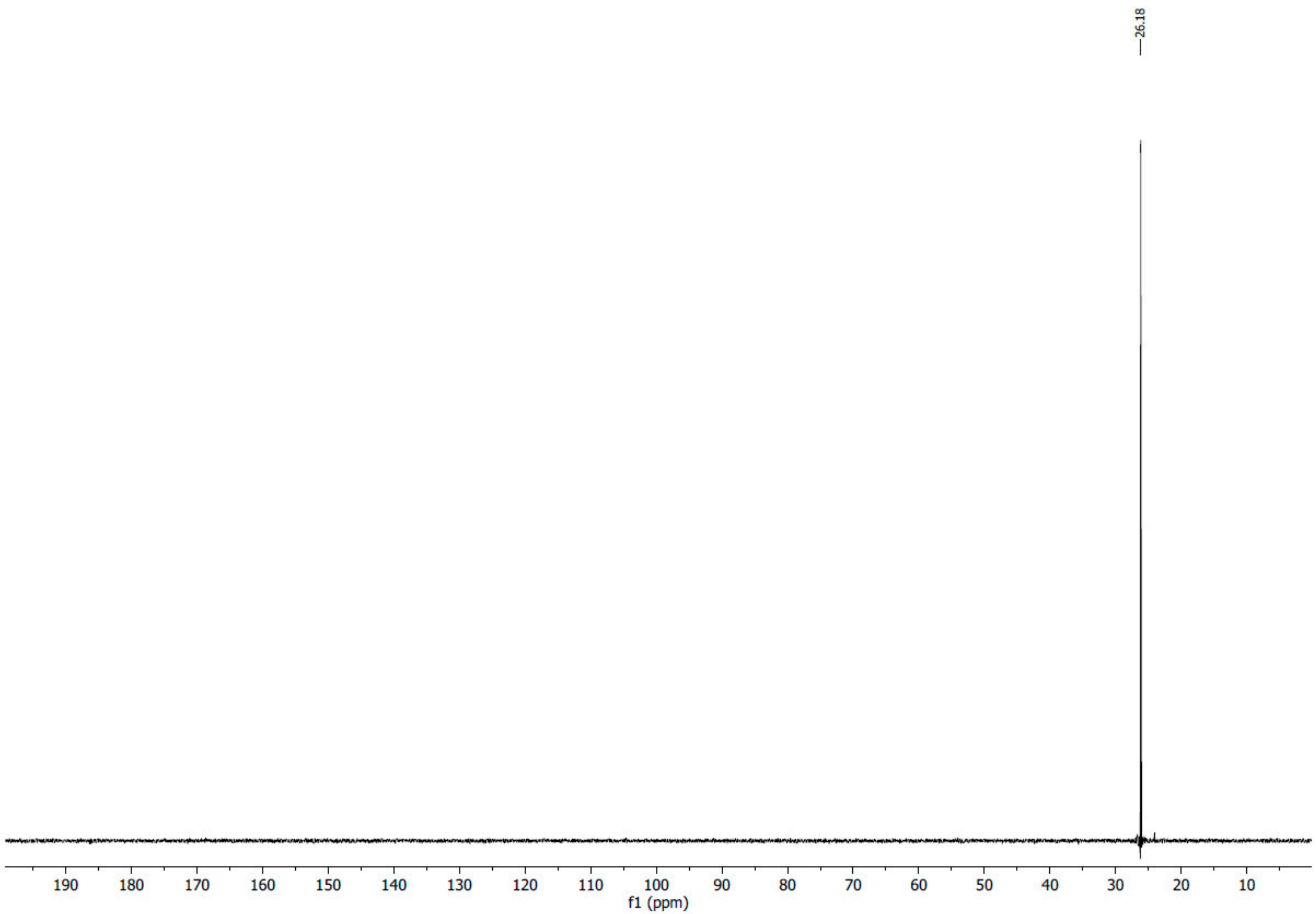

Figure S24. ^{31}PNMR (162 MHz, $CDCl_3$) spectra of compound **7**

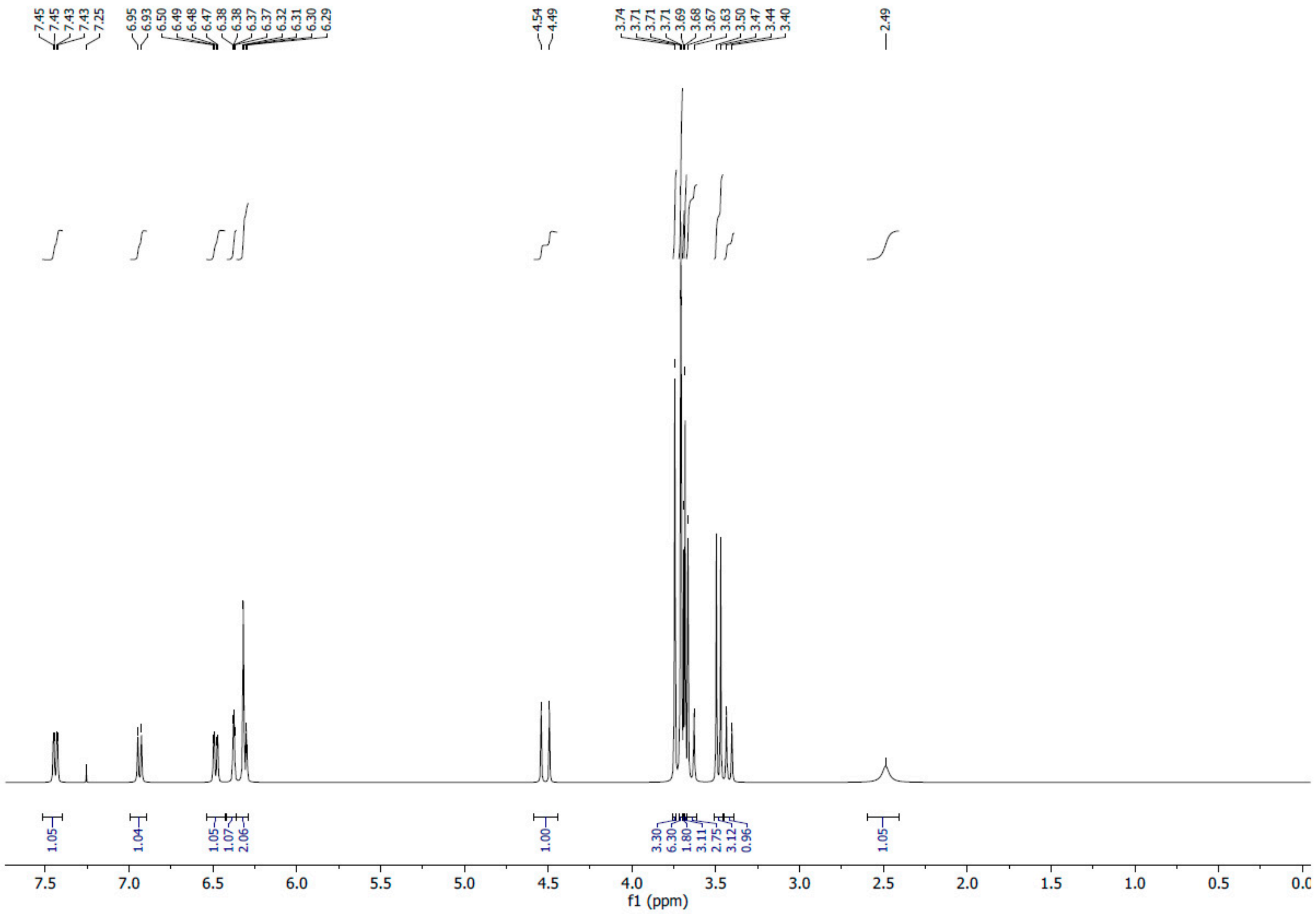

Figure S25. ^{1}HNMR (400 MHz, $CDCl_3$) spectra of compound **8**

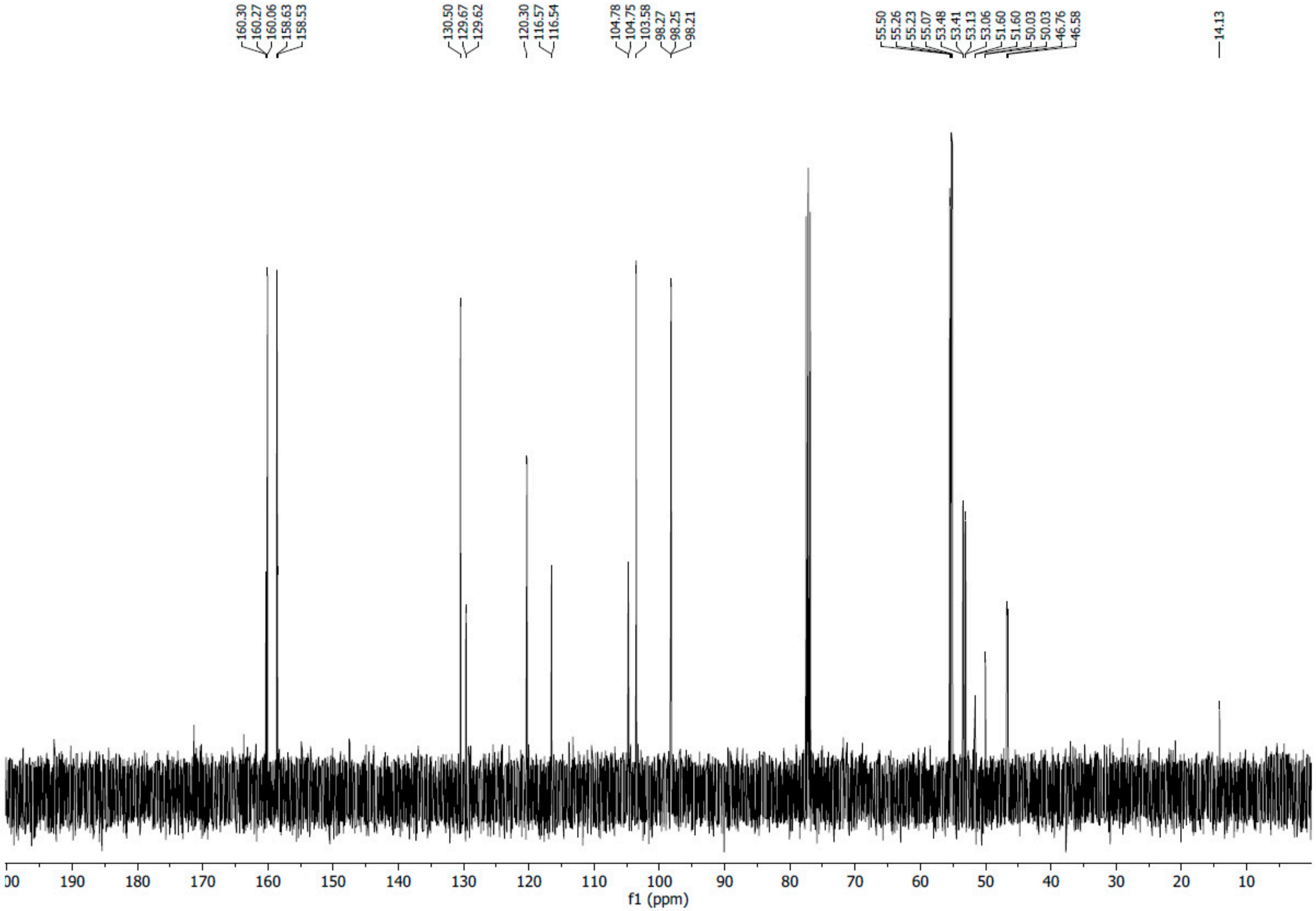

Figure S26. ^{13}CNMR (100 MHz, $CDCl_3$) spectra of compound **8**

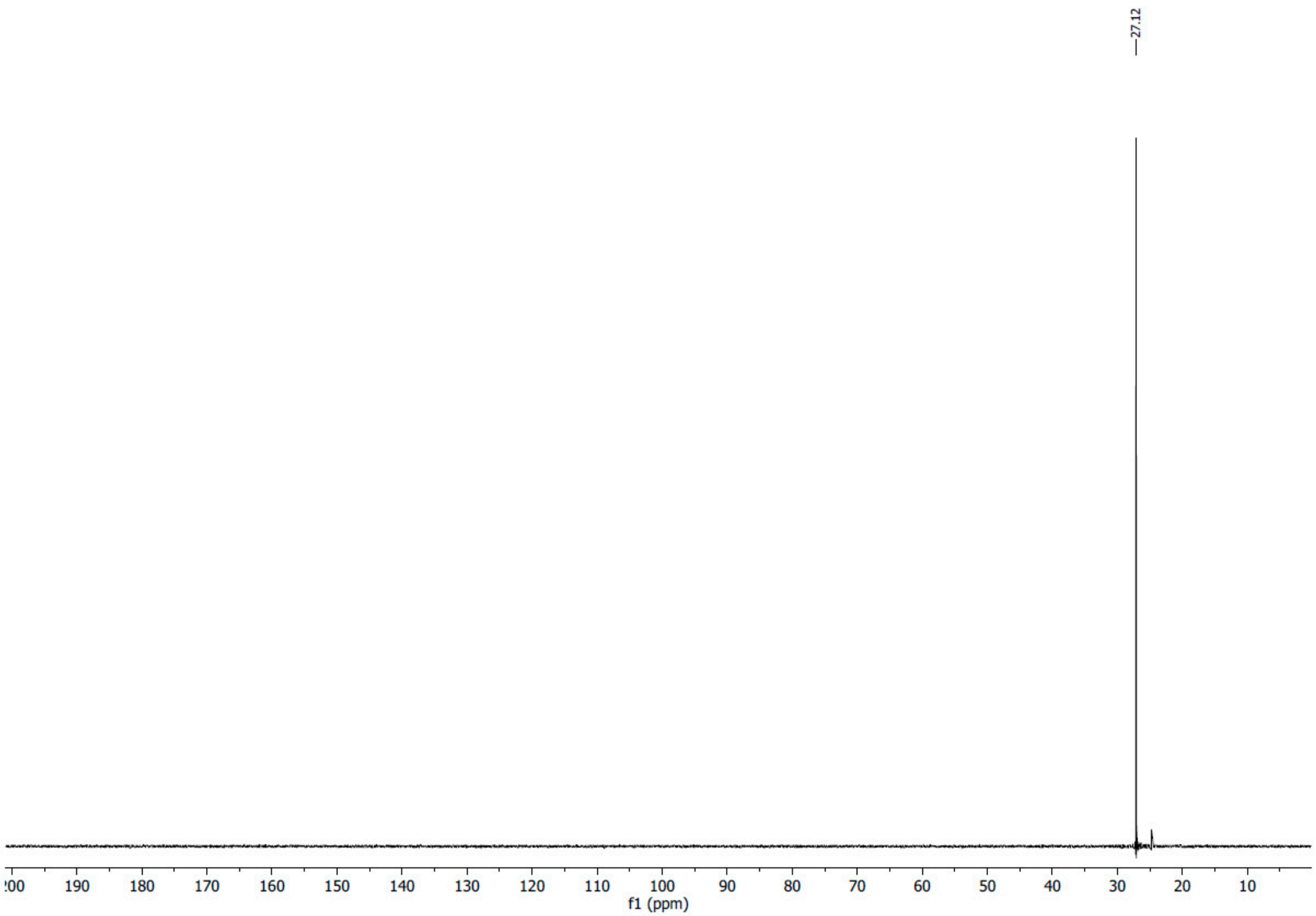

Figure S27. ^{31}PNMR (162 MHz, $CDCl_3$) spectra of compound **8**

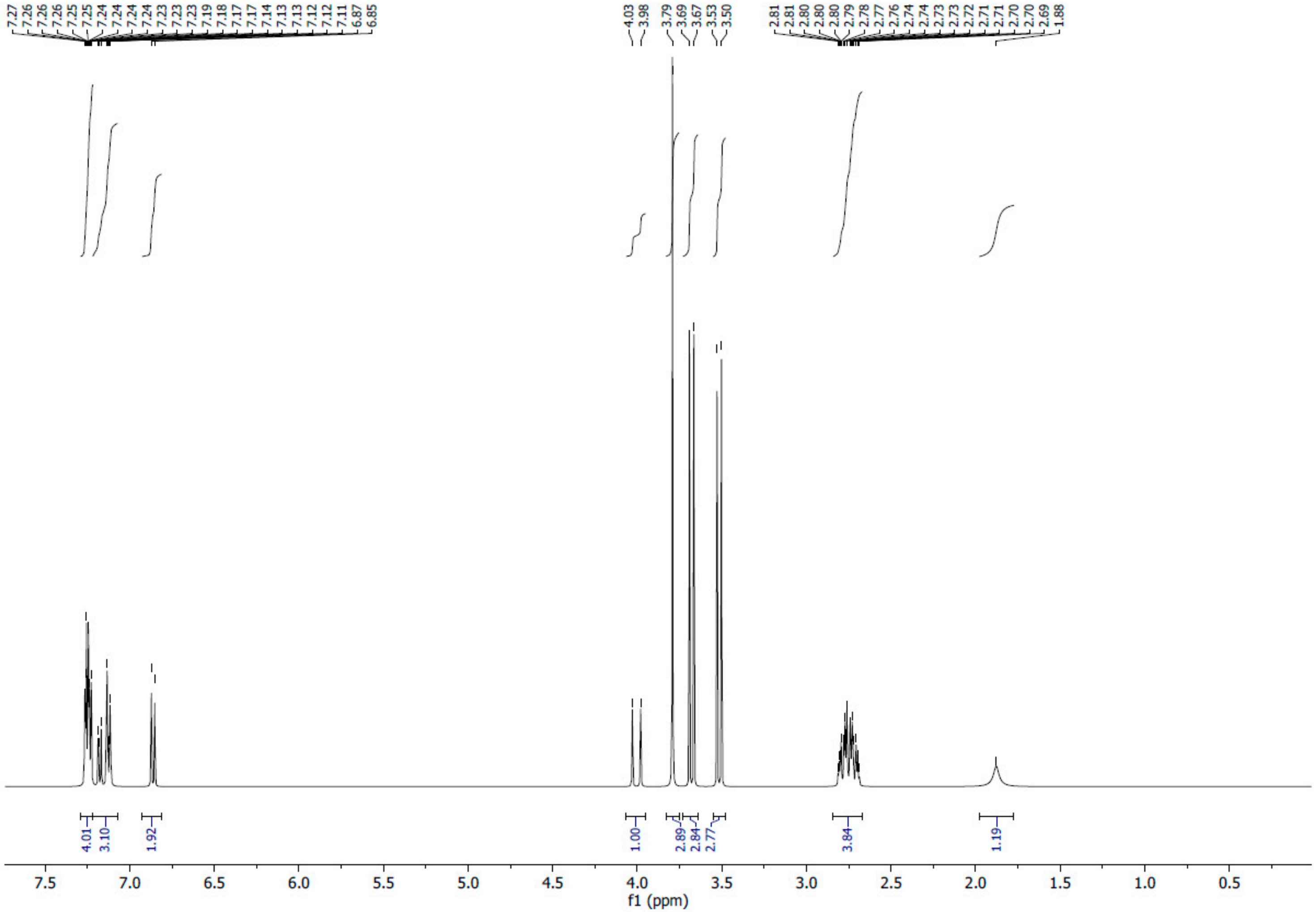

Figure S28. ^{1}HNMR (400 MHz, $CDCl_3$) spectra of compound **9**

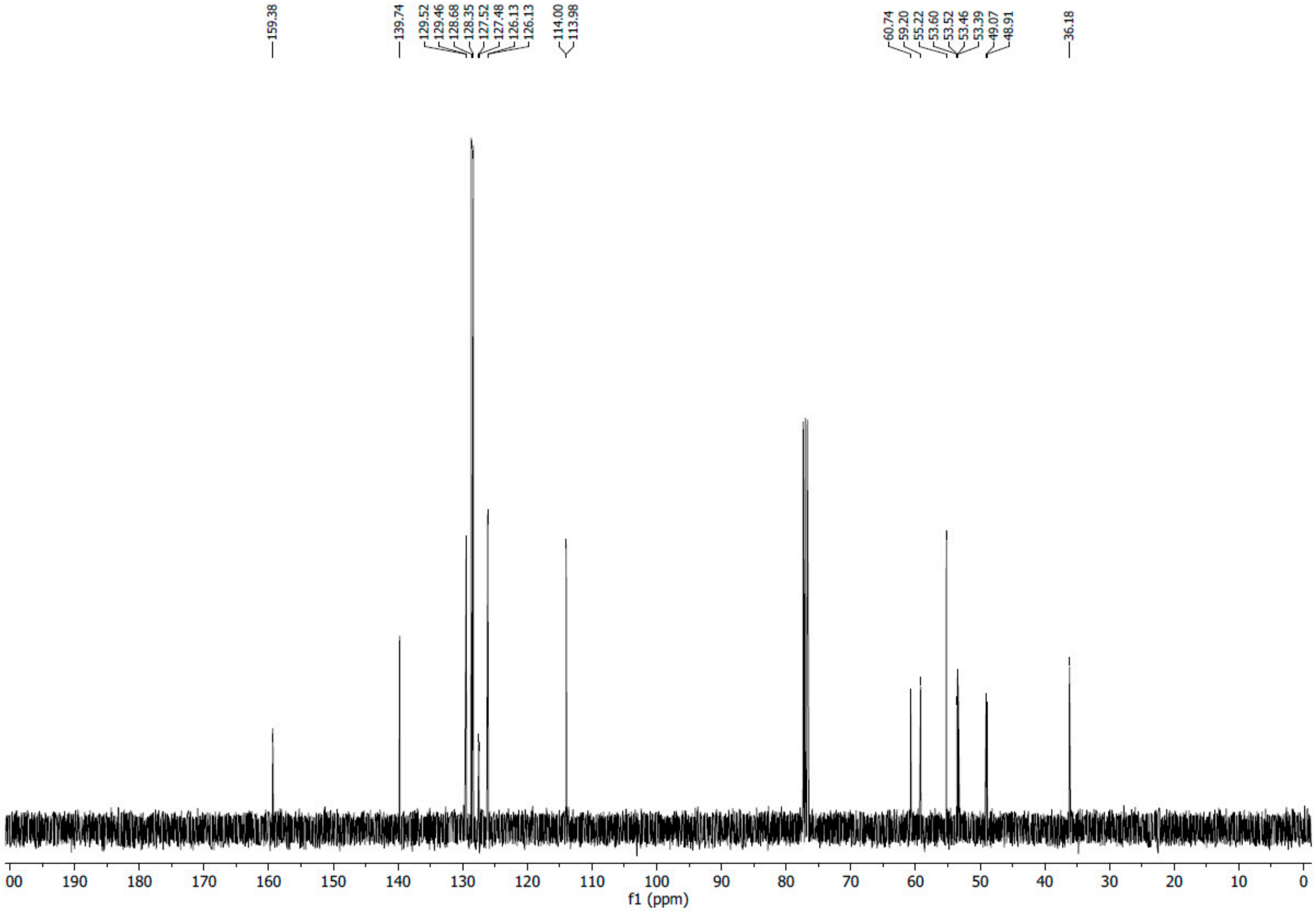

Figure S29. ^{13}CNMR (100 MHz, $CDCl_3$) spectra of compound **9**

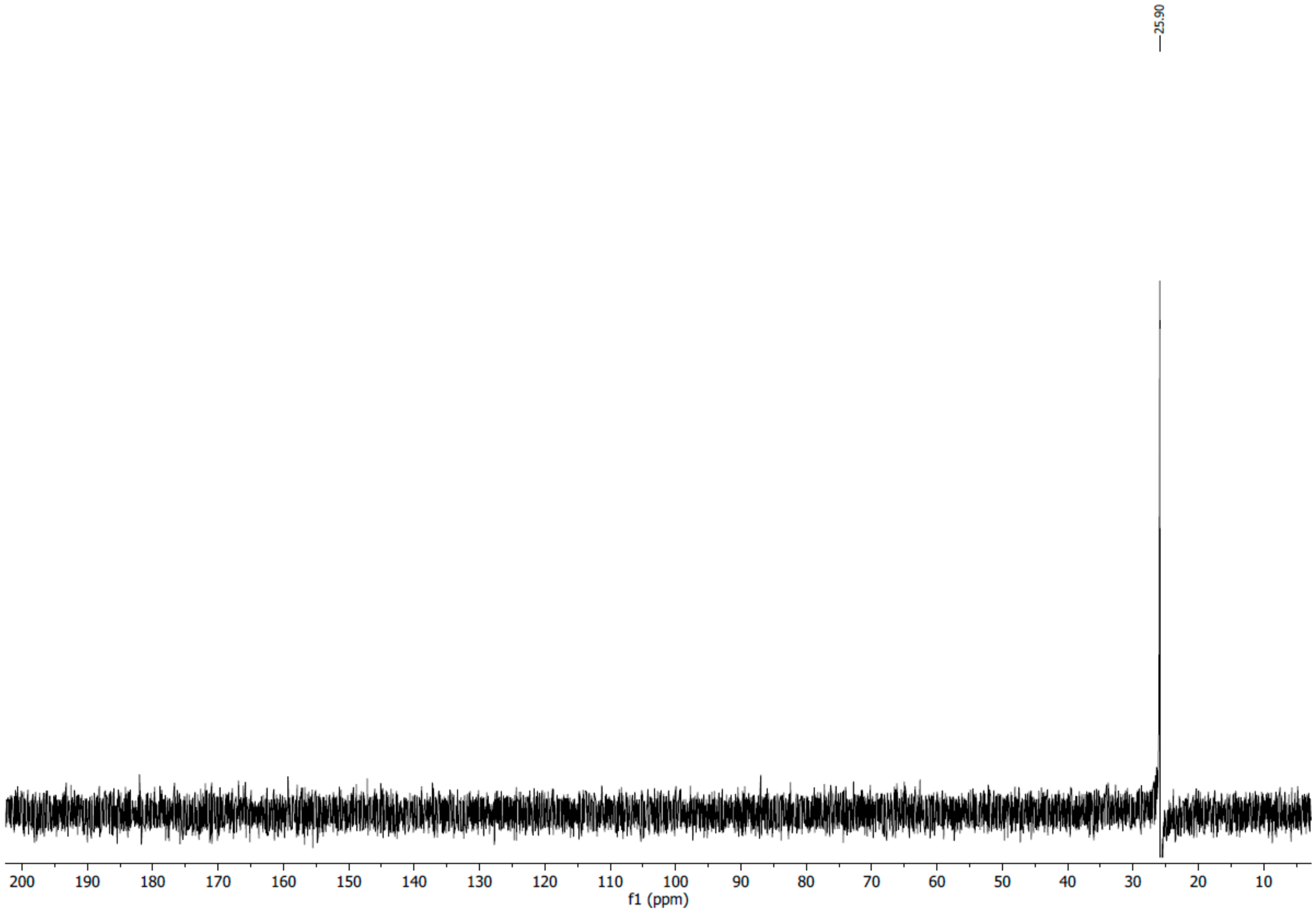

Figure S30. ^{31}PNMR (162 MHz, $CDCl_3$) spectra of compound **9**

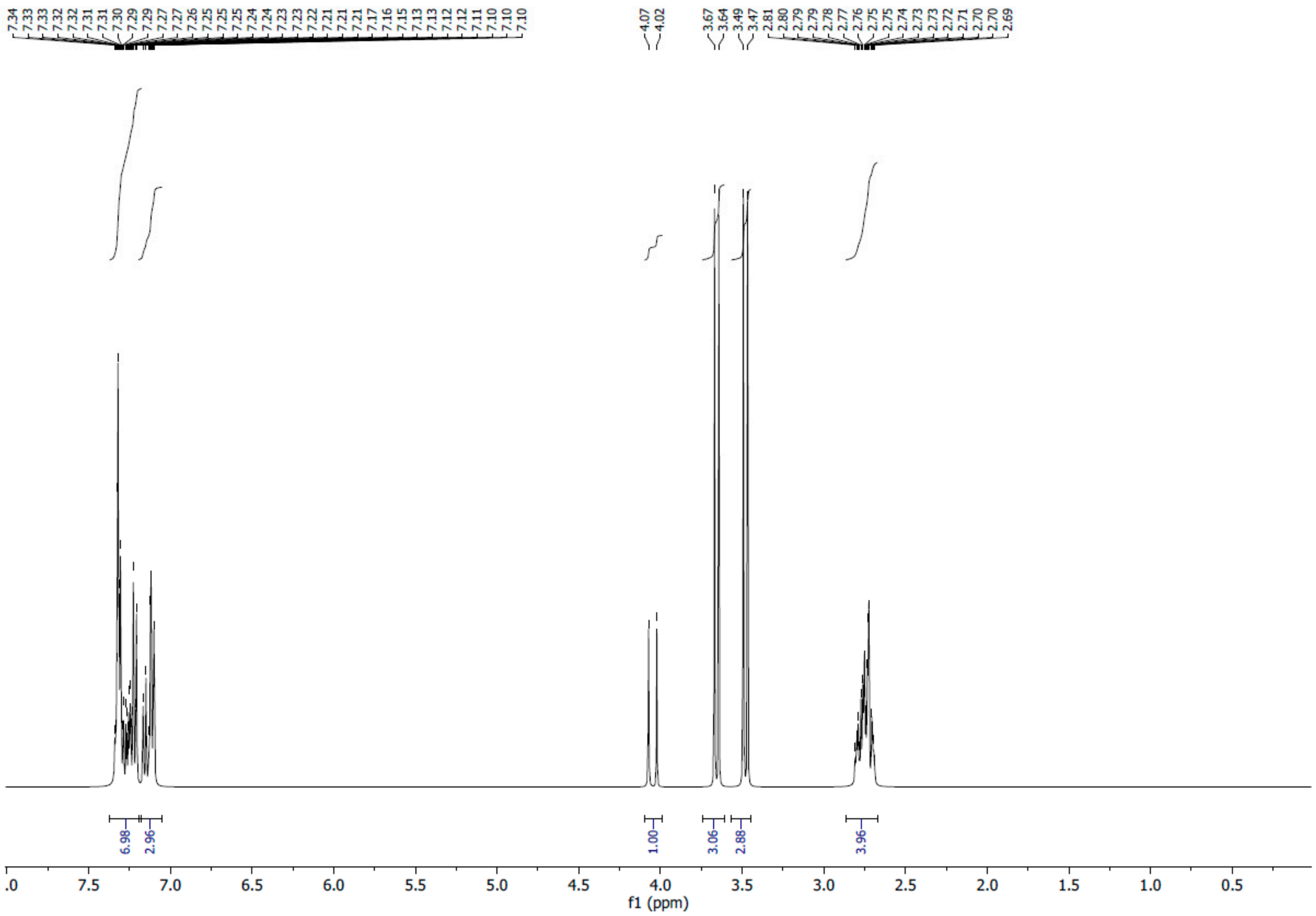

Figure S31. ^{1}HNMR (400 MHz, $CDCl_3$) spectra of compound **10**

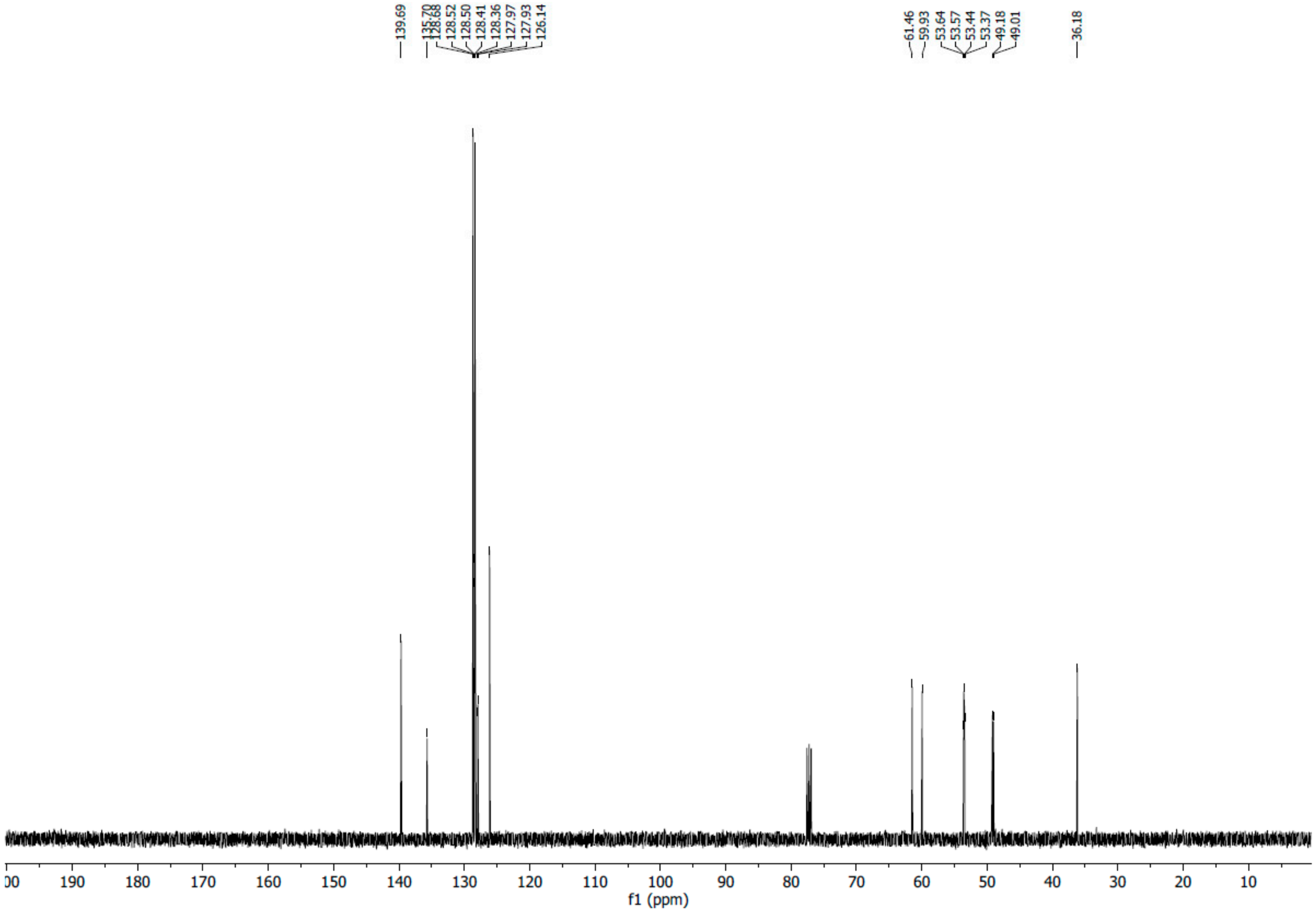

Figure S32. ^{13}CNMR (100 MHz, $CDCl_3$) spectra of compound **10**

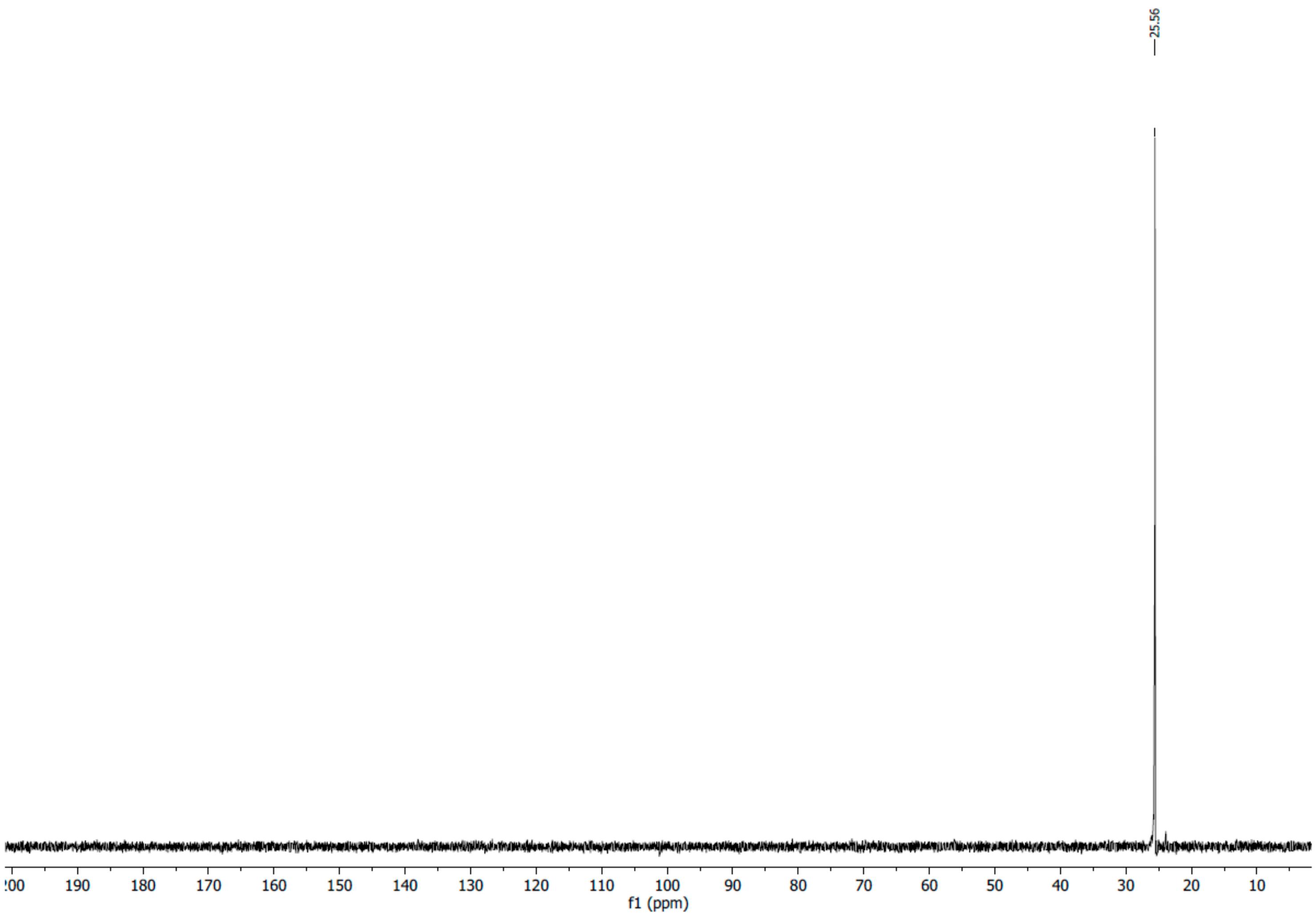

Figure S33. ^{31}PNMR (162 MHz, $CDCl_3$) spectra of compound **10**

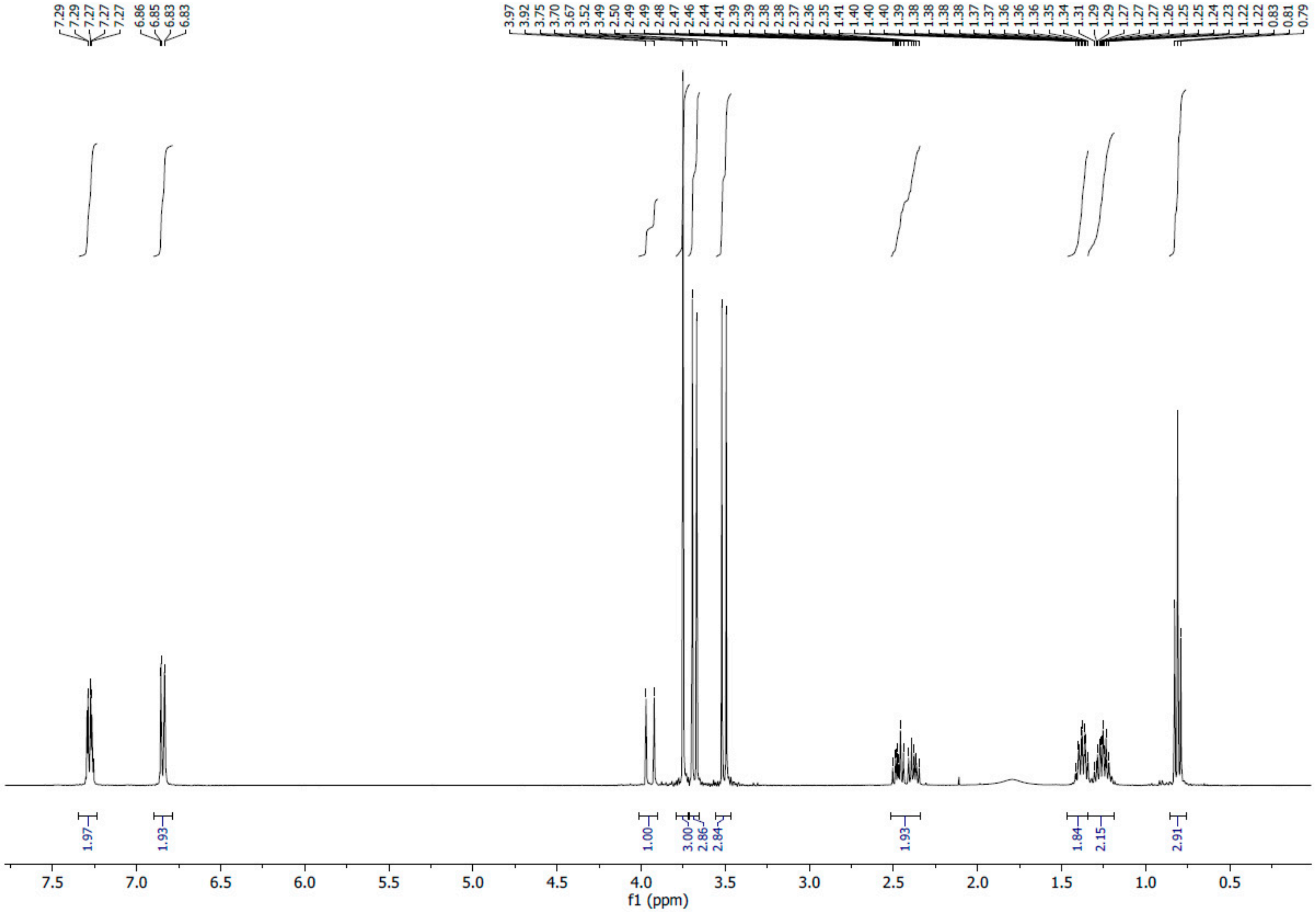

Figure S34. ^{1}HNMR (400 MHz, $CDCl_3$) spectra of compound **11**

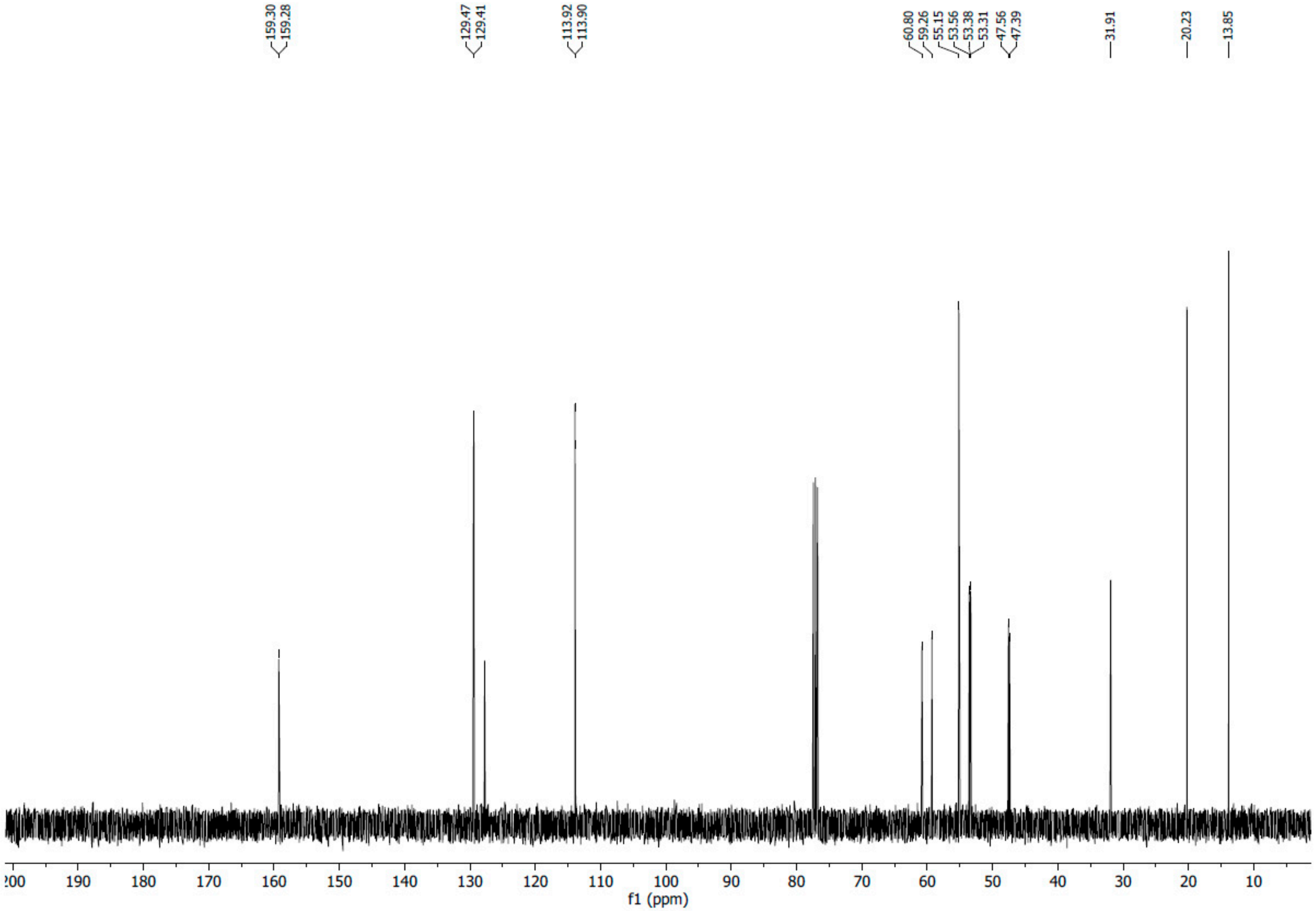

Figure S35. ^{13}CNMR (100 MHz, $CDCl_3$) spectra of compound **11**

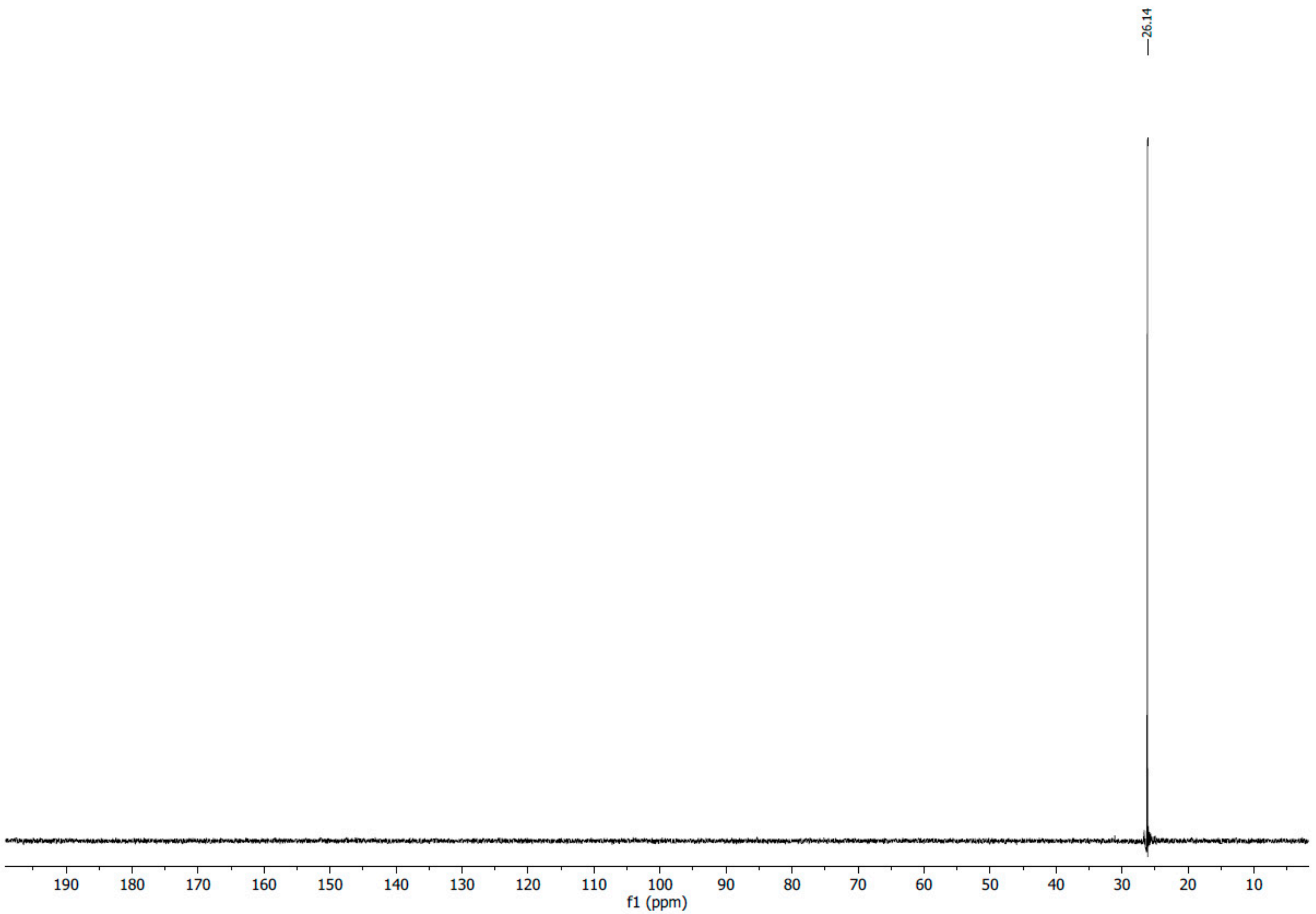

Figure S36. ^{31}PNMR (162 MHz, $CDCl_3$) spectra of compound **11**

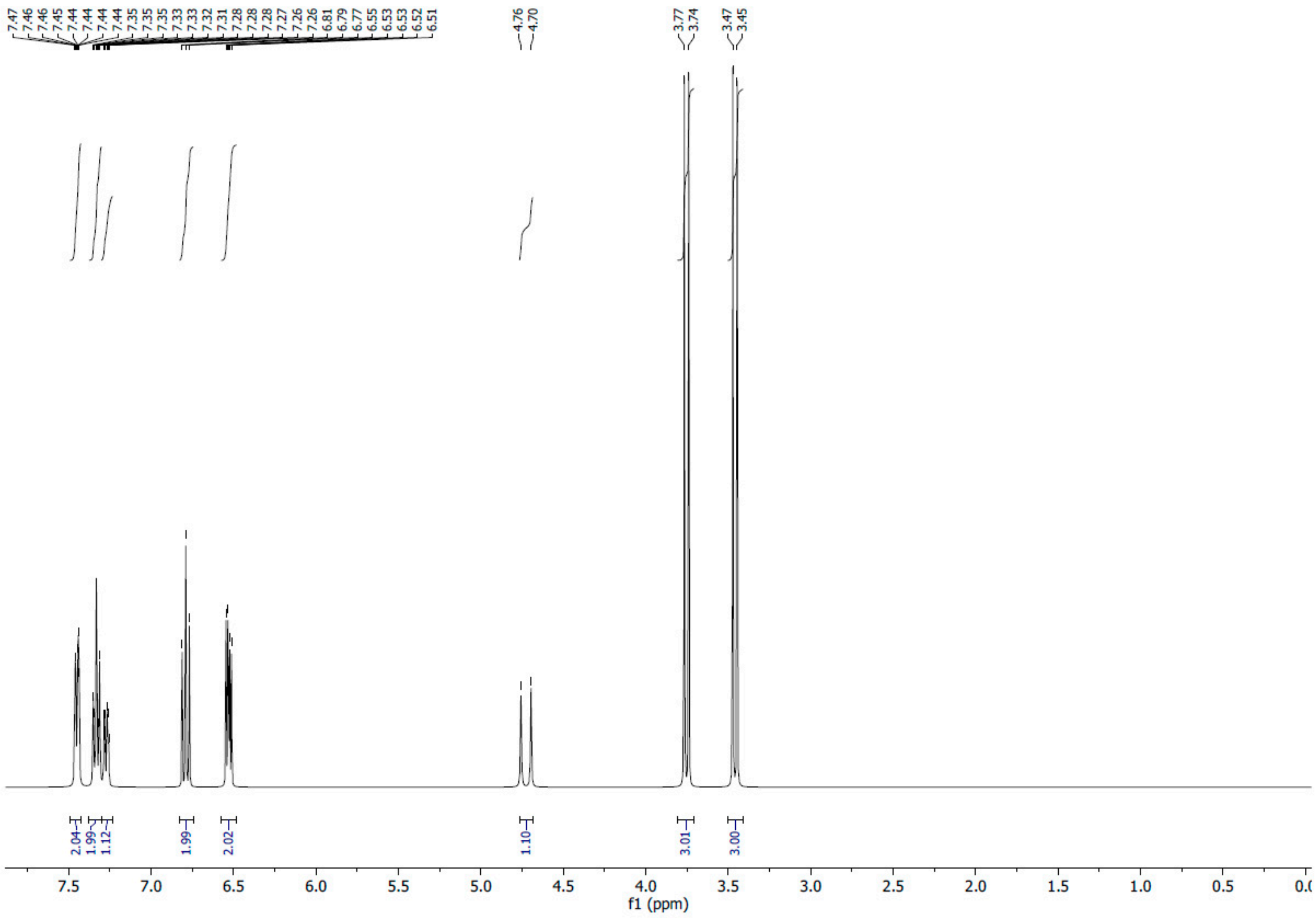

Figure S37. ^{1}HNMR (400 MHz, $CDCl_3$) spectra of compound **12**

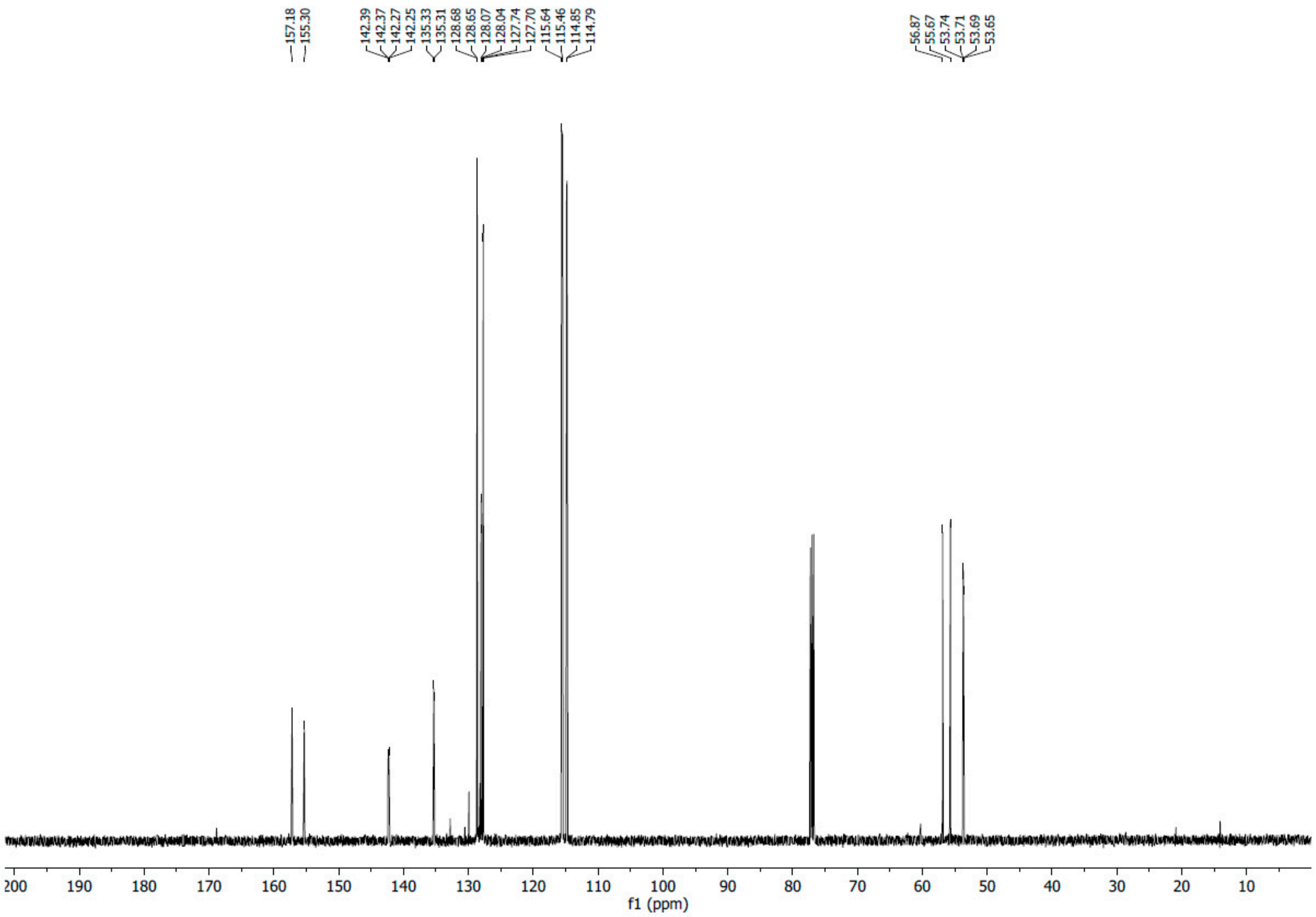

Figure S38. ^{13}CNMR (100 MHz, $CDCl_3$) spectra of compound **12**

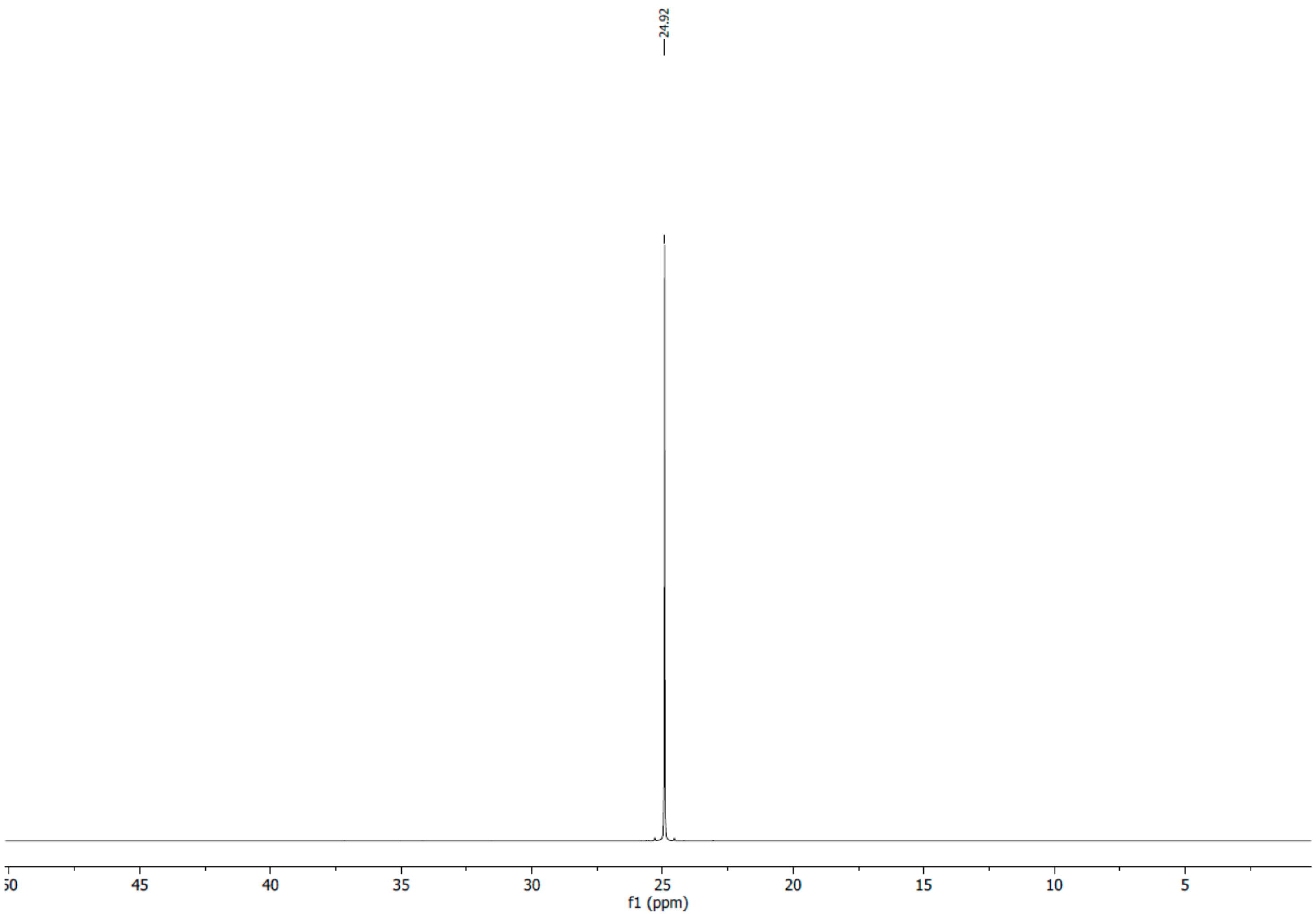

Figure S39. ^{31}PNMR (162 MHz, $CDCl_3$) spectra of compound **12**

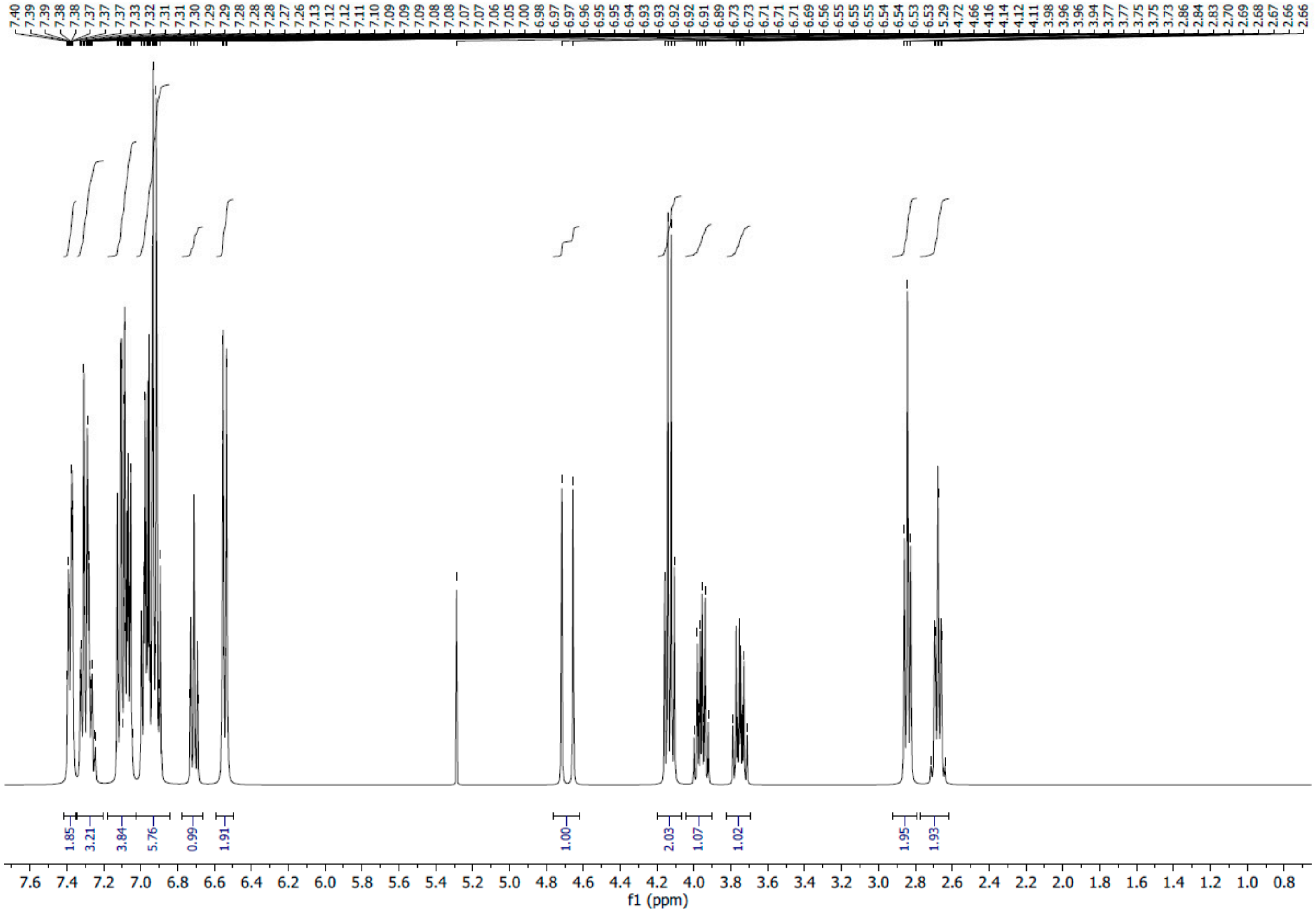

Figure S40. ^{1}HNMR (400 MHz, $CDCl_3$) spectra of compound **13**

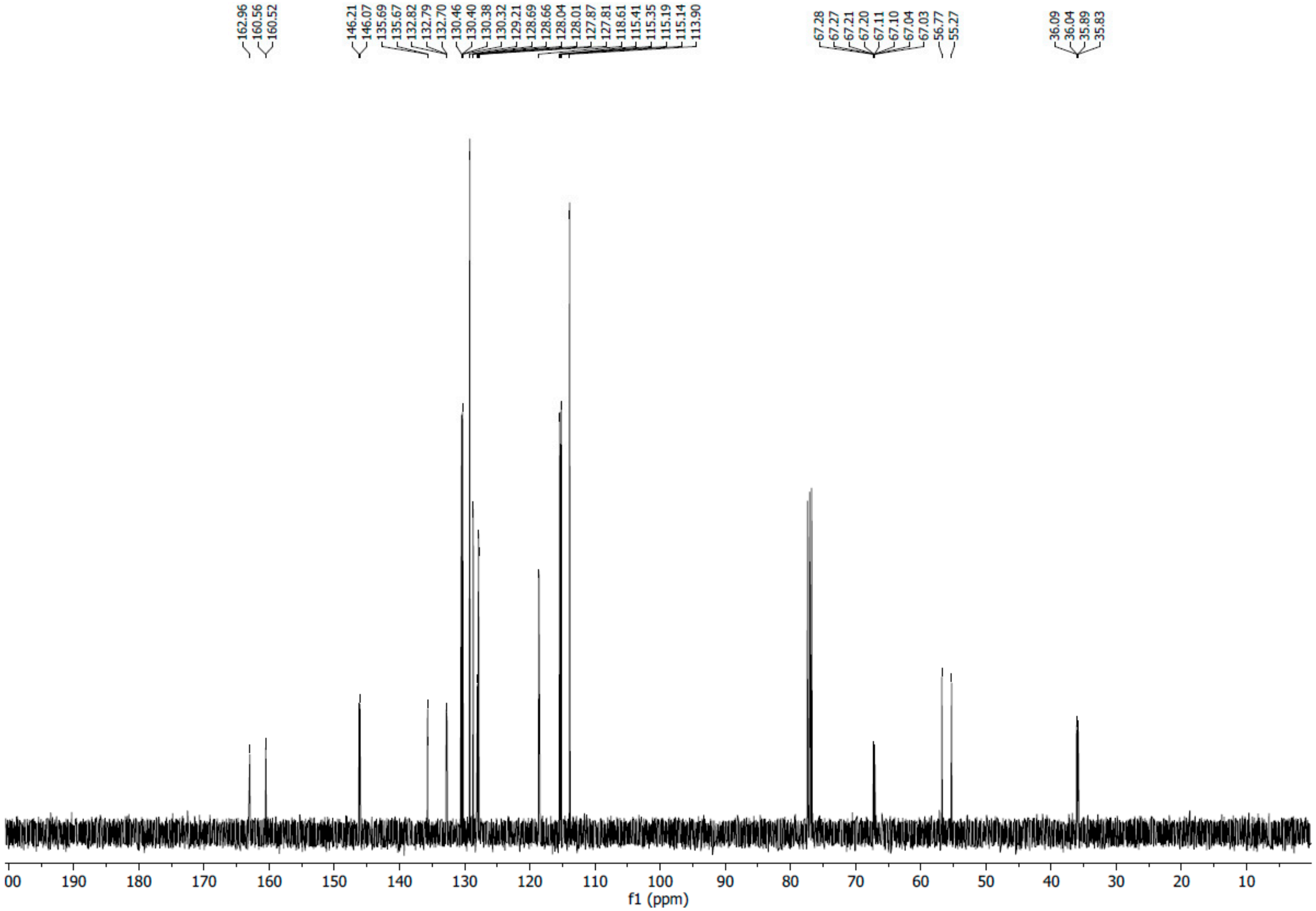

Figure S41. ^{13}CNMR (100 MHz, $CDCl_3$) spectra of compound **13**

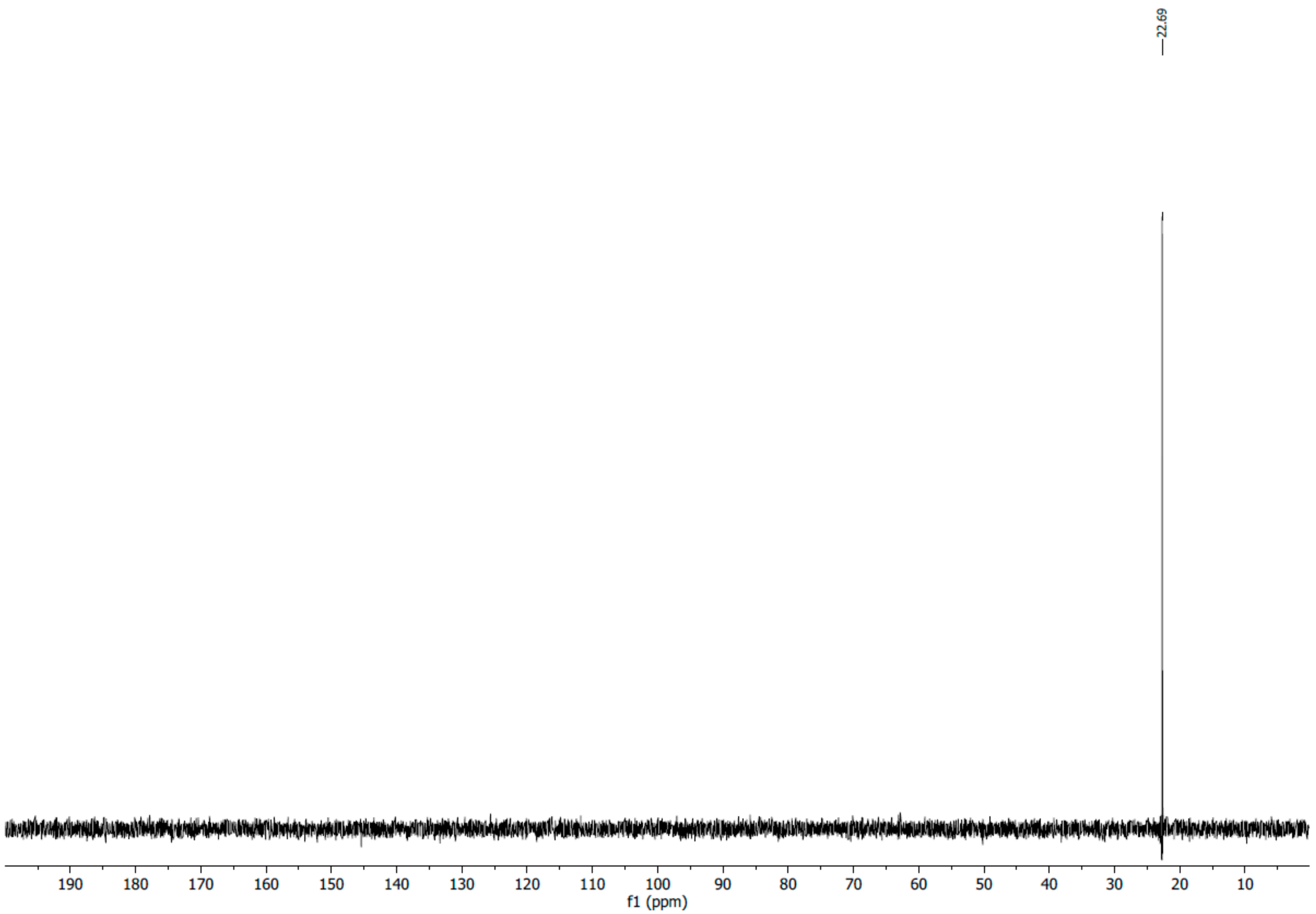

Figure S42. ^{31}PNMR (162 MHz, $CDCl_3$) spectra of compound **13**

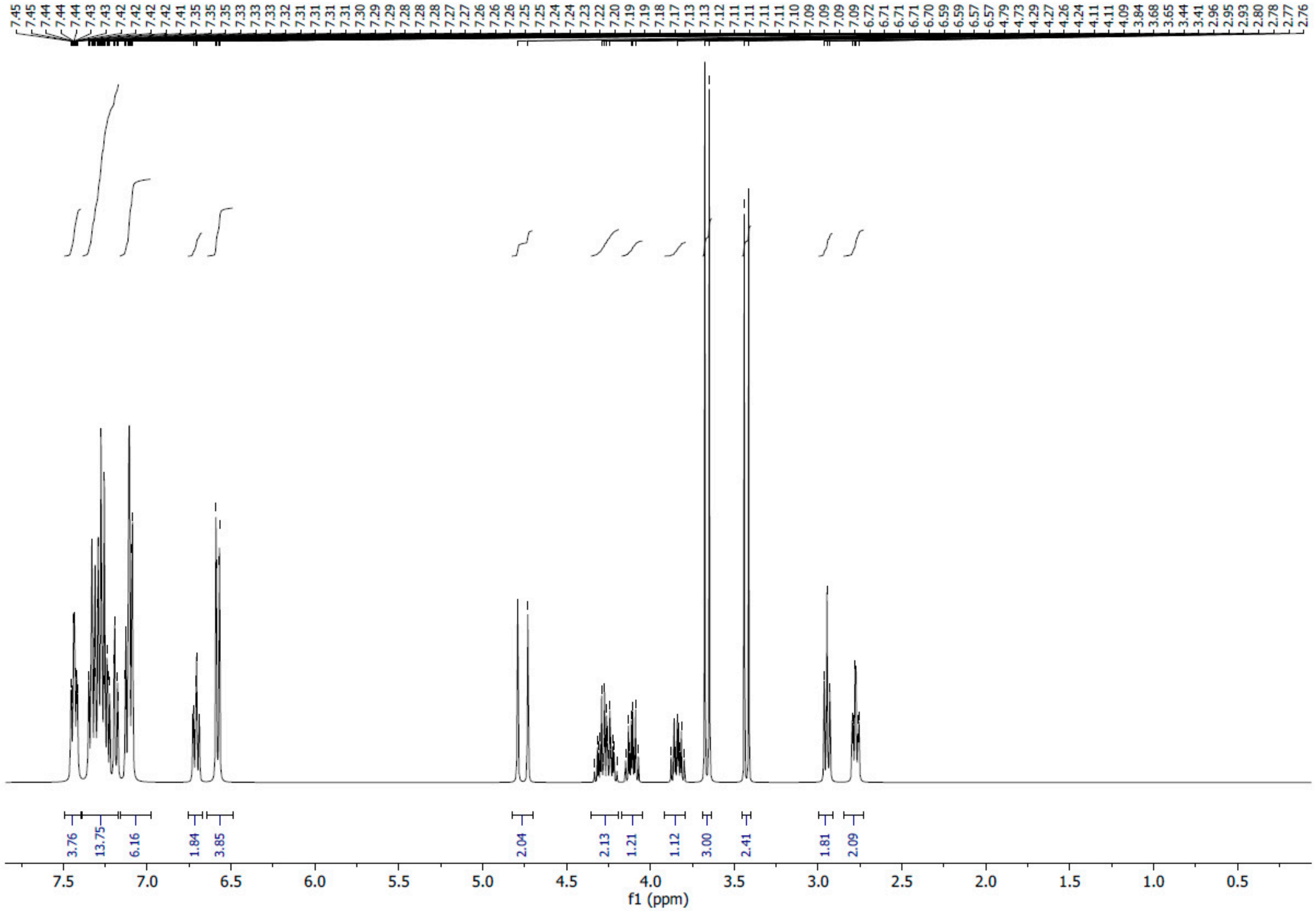

Figure S43. ^{1}HNMR (400 MHz, $CDCl_3$) spectra of compound **14**

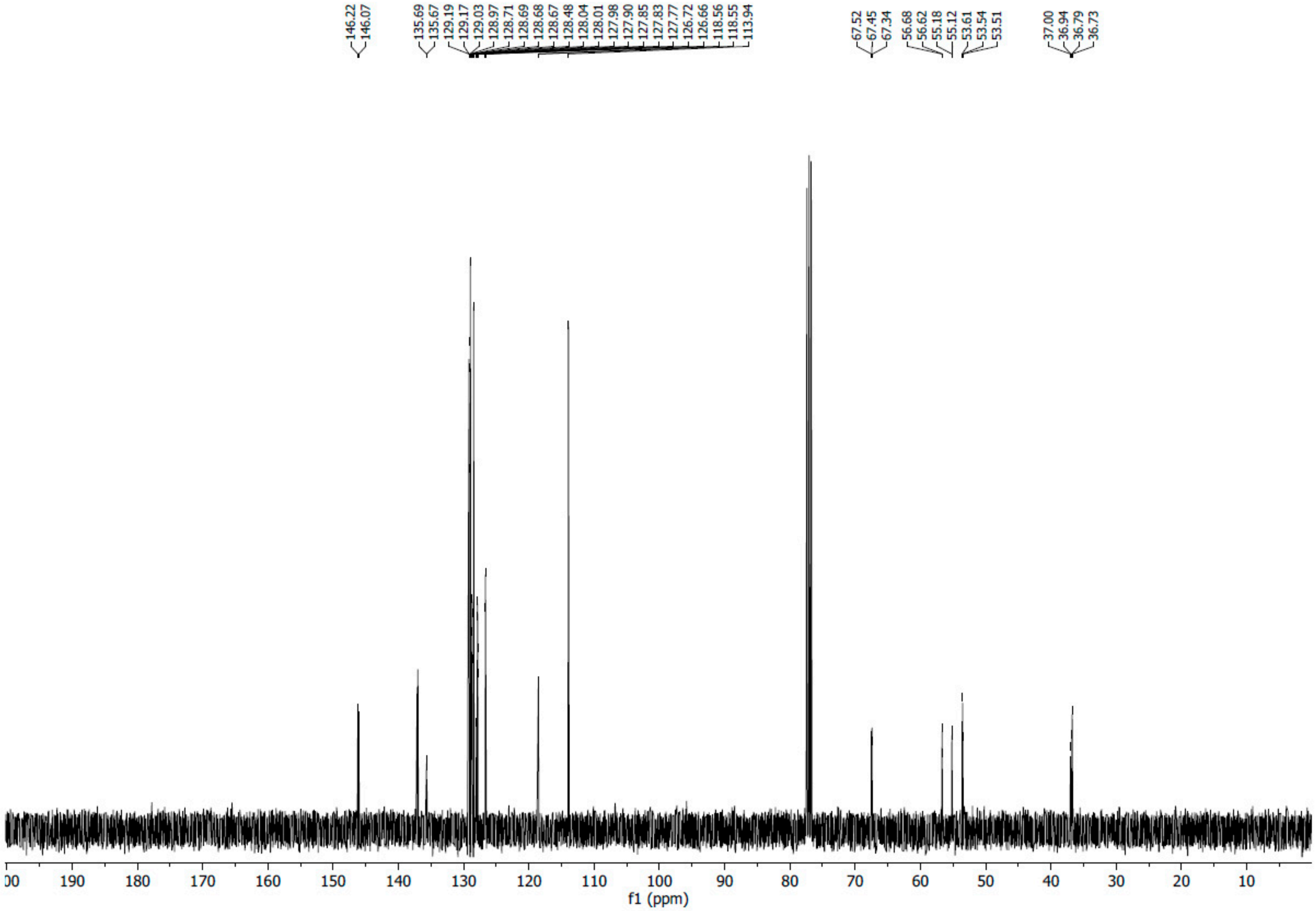

Figure S44. ^{13}CNMR (100 MHz, $CDCl_3$) spectra of compound **14**

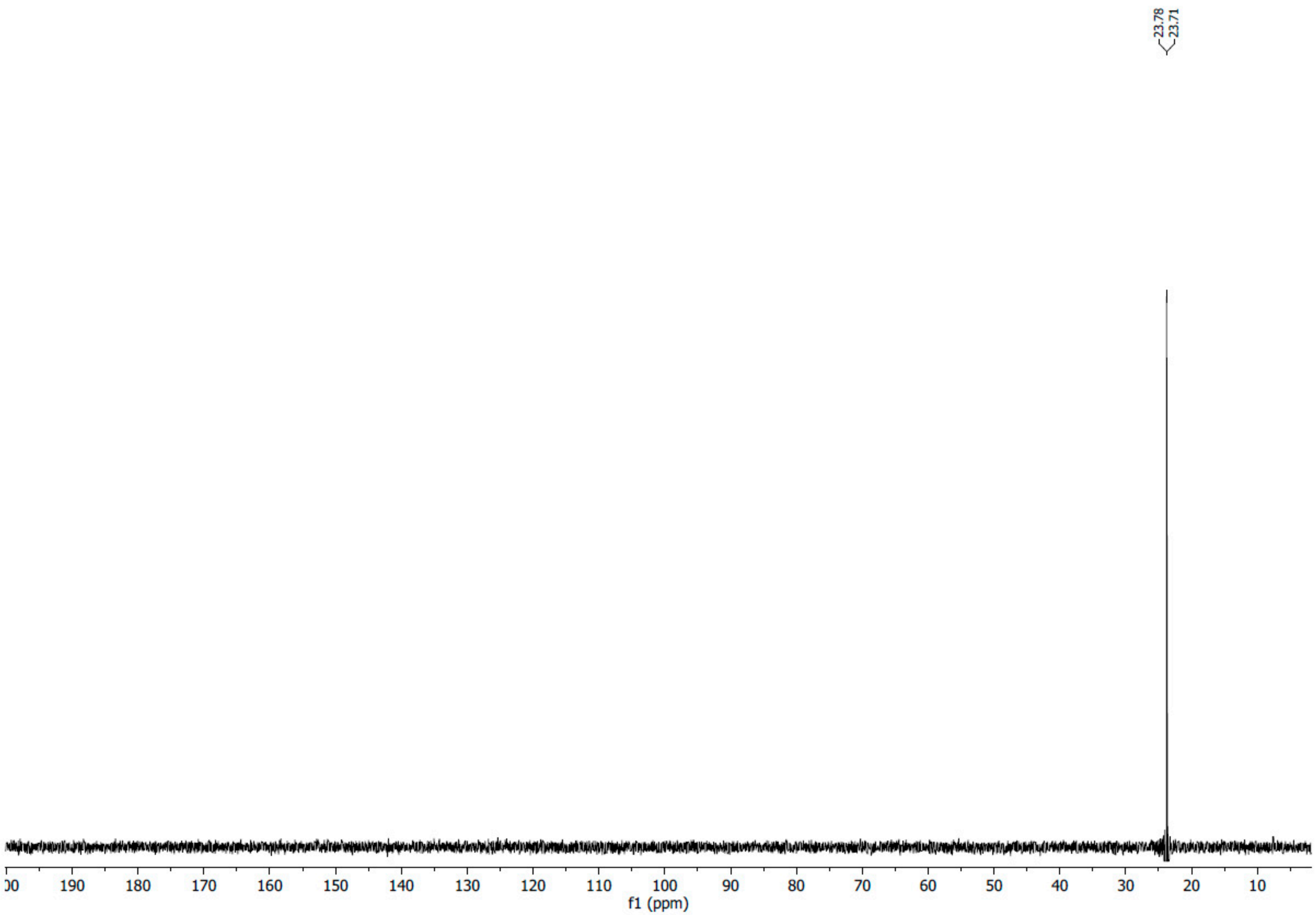

Figure S45. ^{31}PNMR (162 MHz, $CDCl_3$) spectra of compound **14**

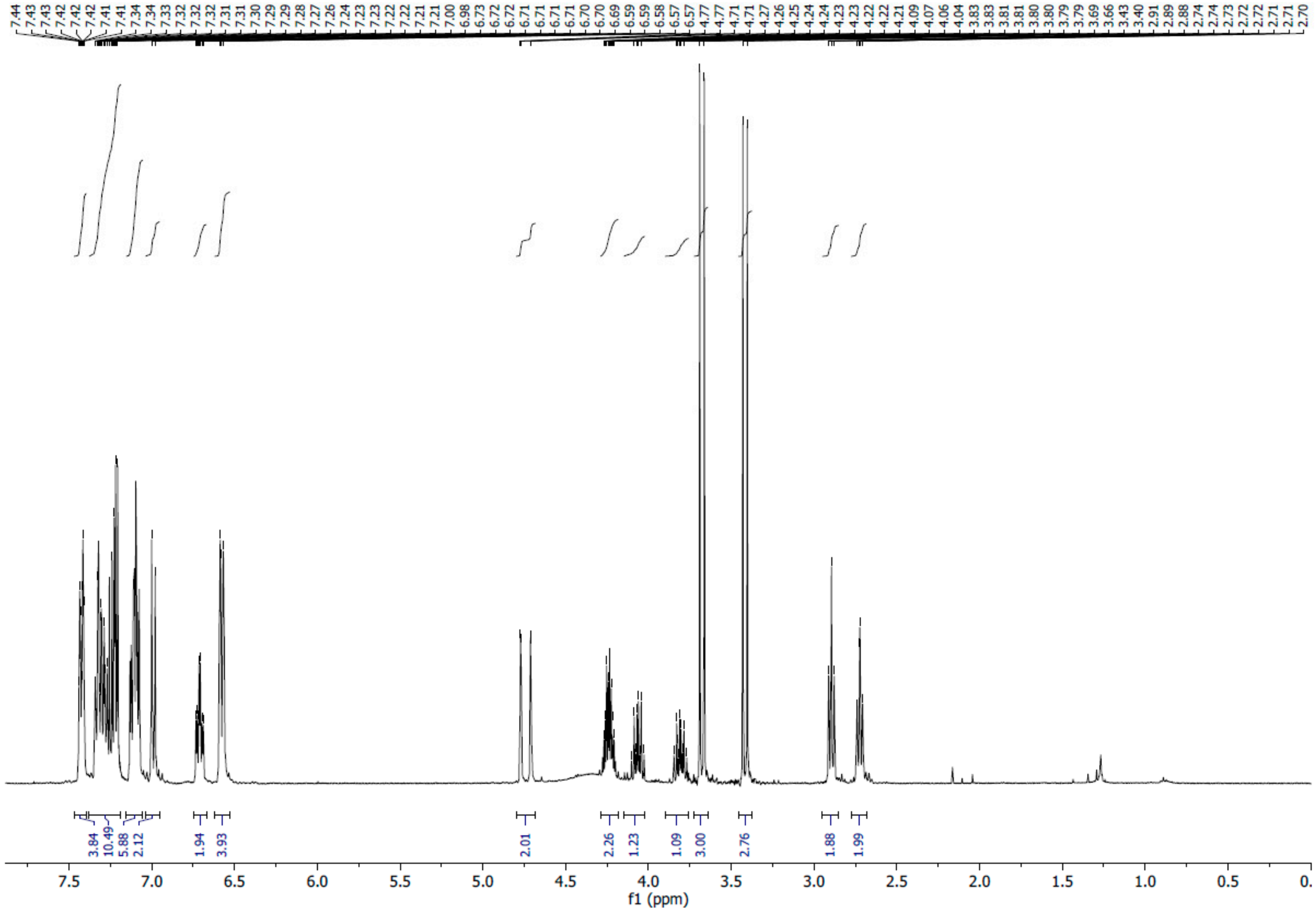

Figure S46. ^{1}HNMR (400 MHz, $CDCl_3$) spectra of compound **15**

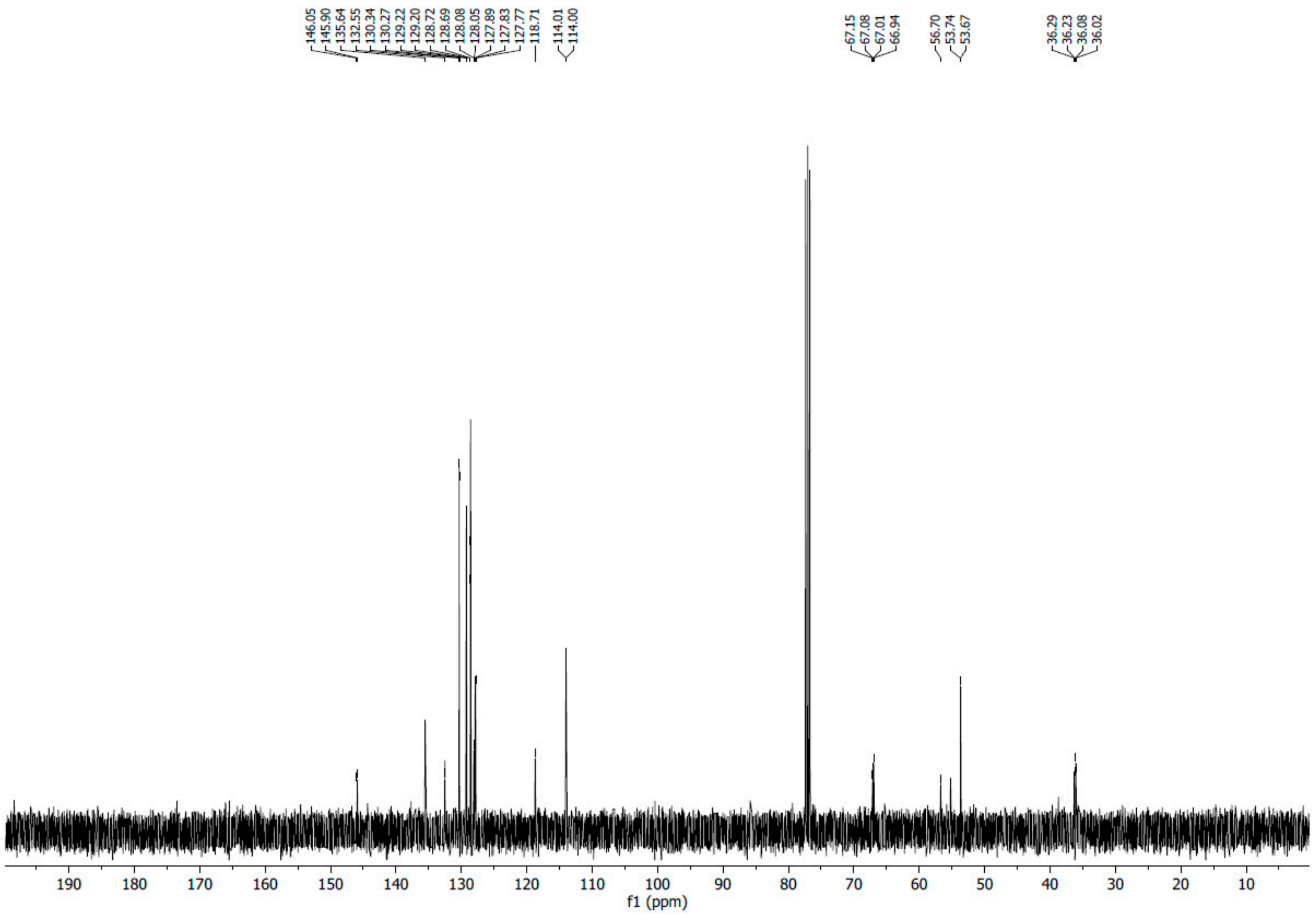

Figure S47. ^{13}CNMR (100 MHz, $CDCl_3$) spectra of compound **15**

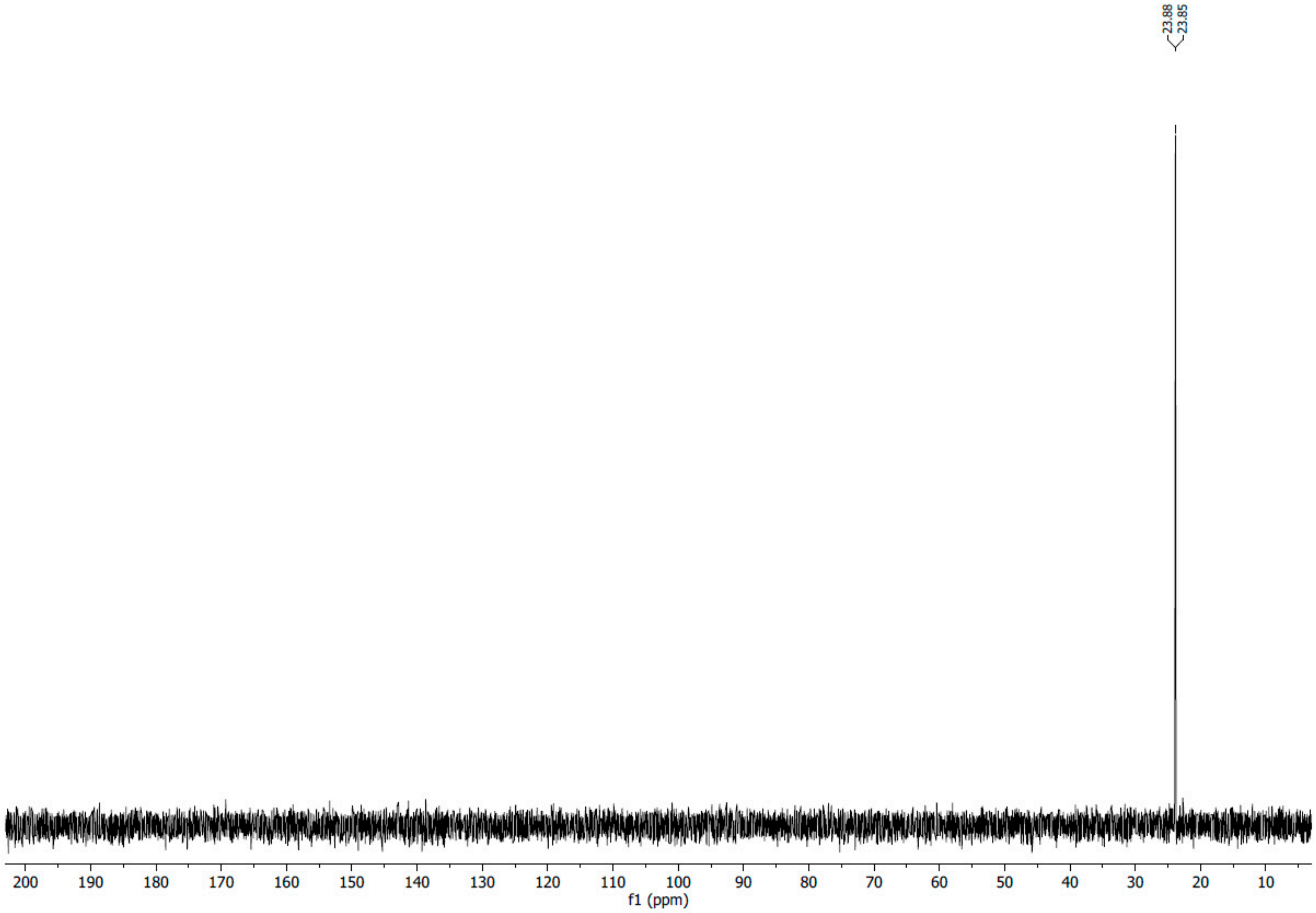

Figure S48. ^{31}PNMR (162 MHz, $CDCl_3$) spectra of compound **15**

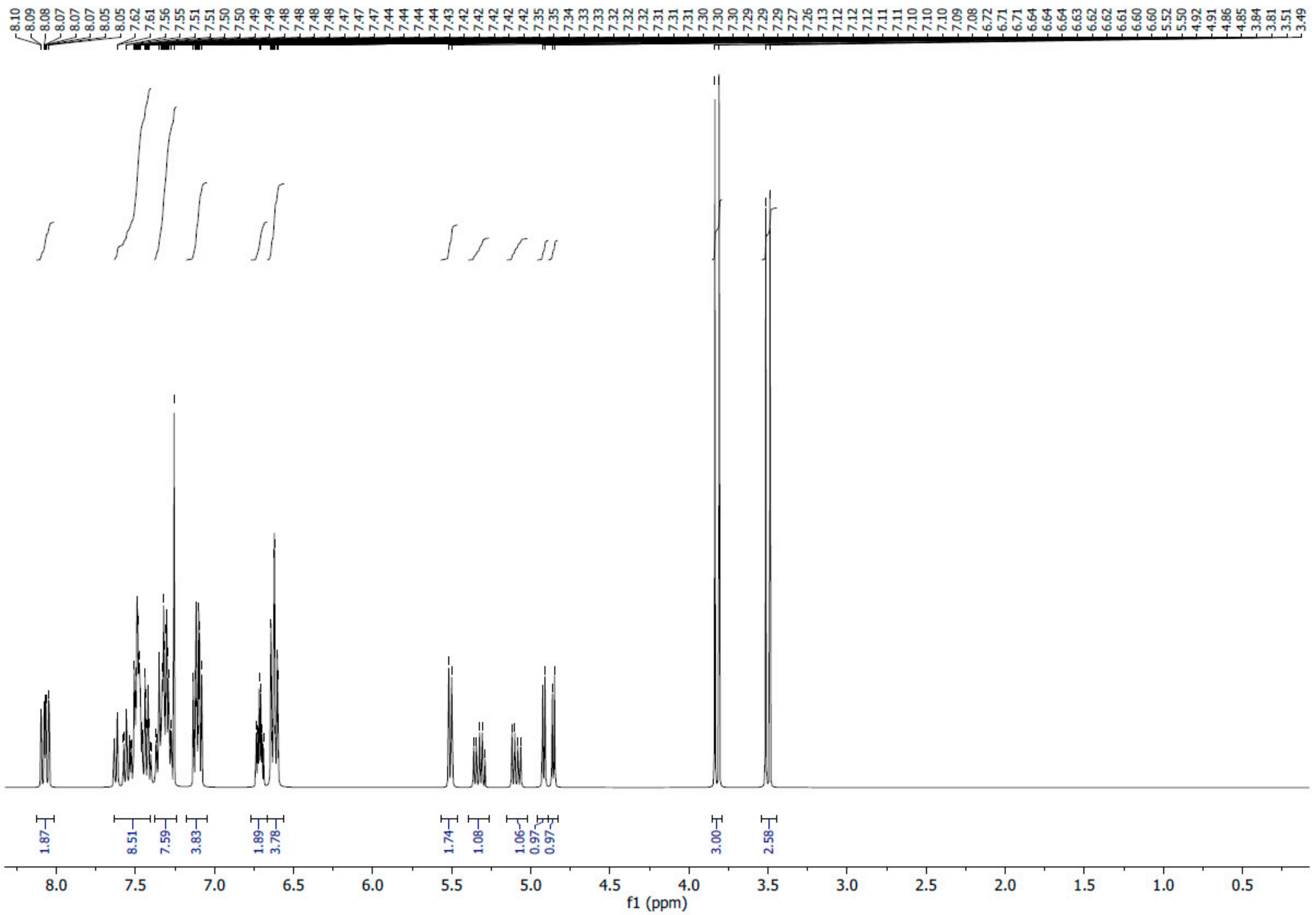

Figure S49. ^{1}HNMR (400 MHz, $CDCl_3$) spectra of compound **16**

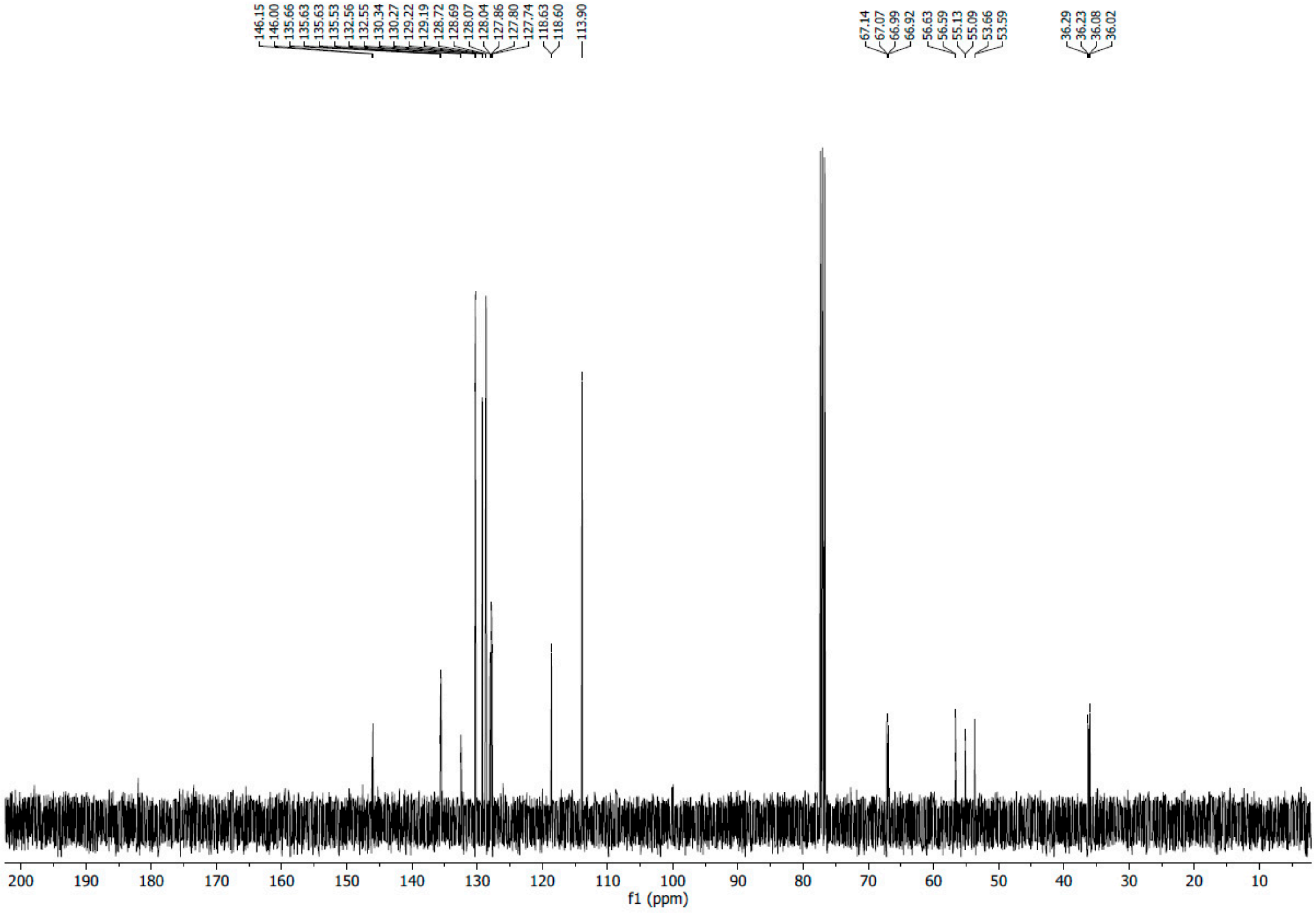

Figure S50. ^{13}CNMR (100 MHz, $CDCl_3$) spectra of compound **16**

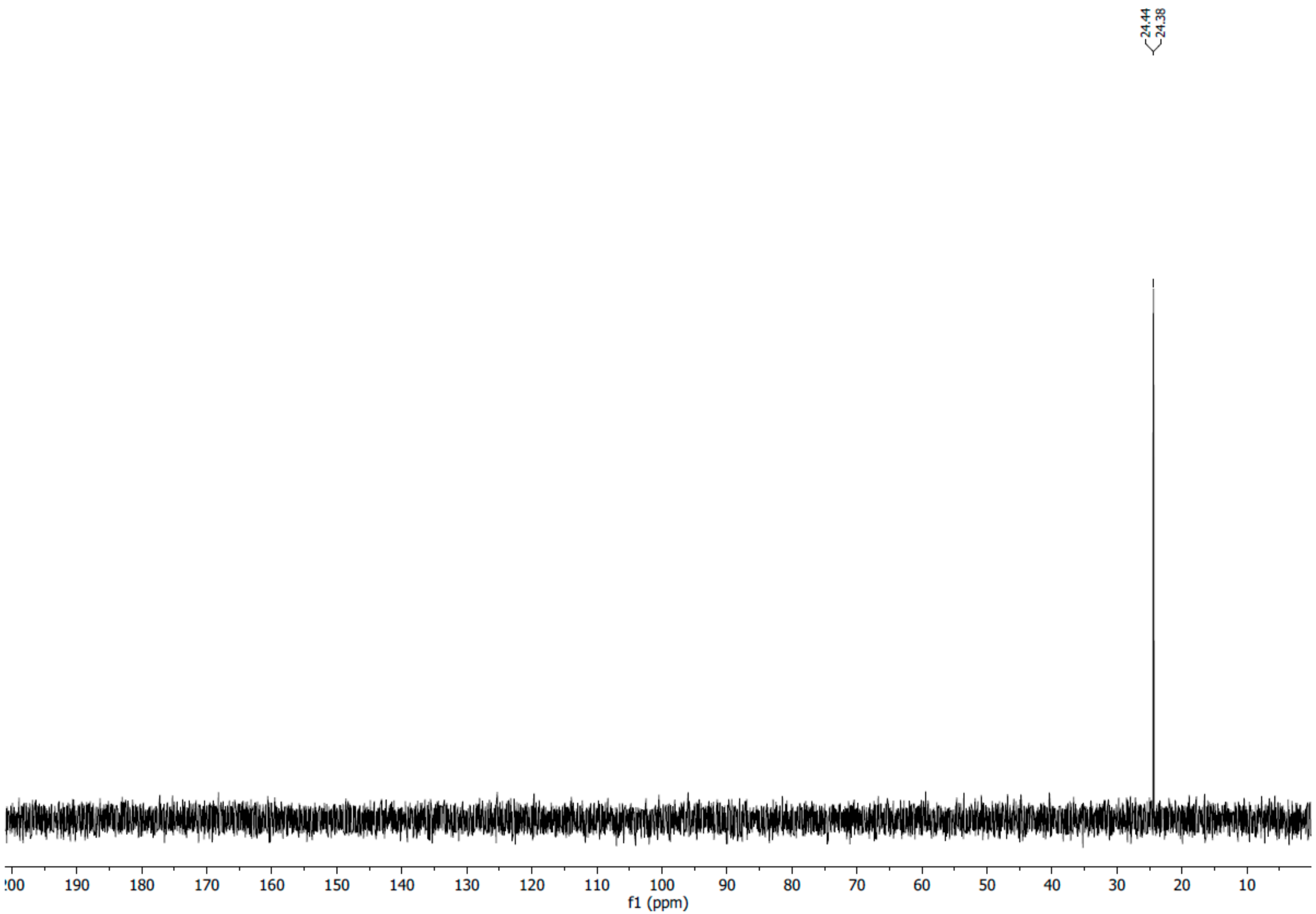

Figure S51. ^{31}PNMR (162 MHz, $CDCl_3$) spectra of compound **16**

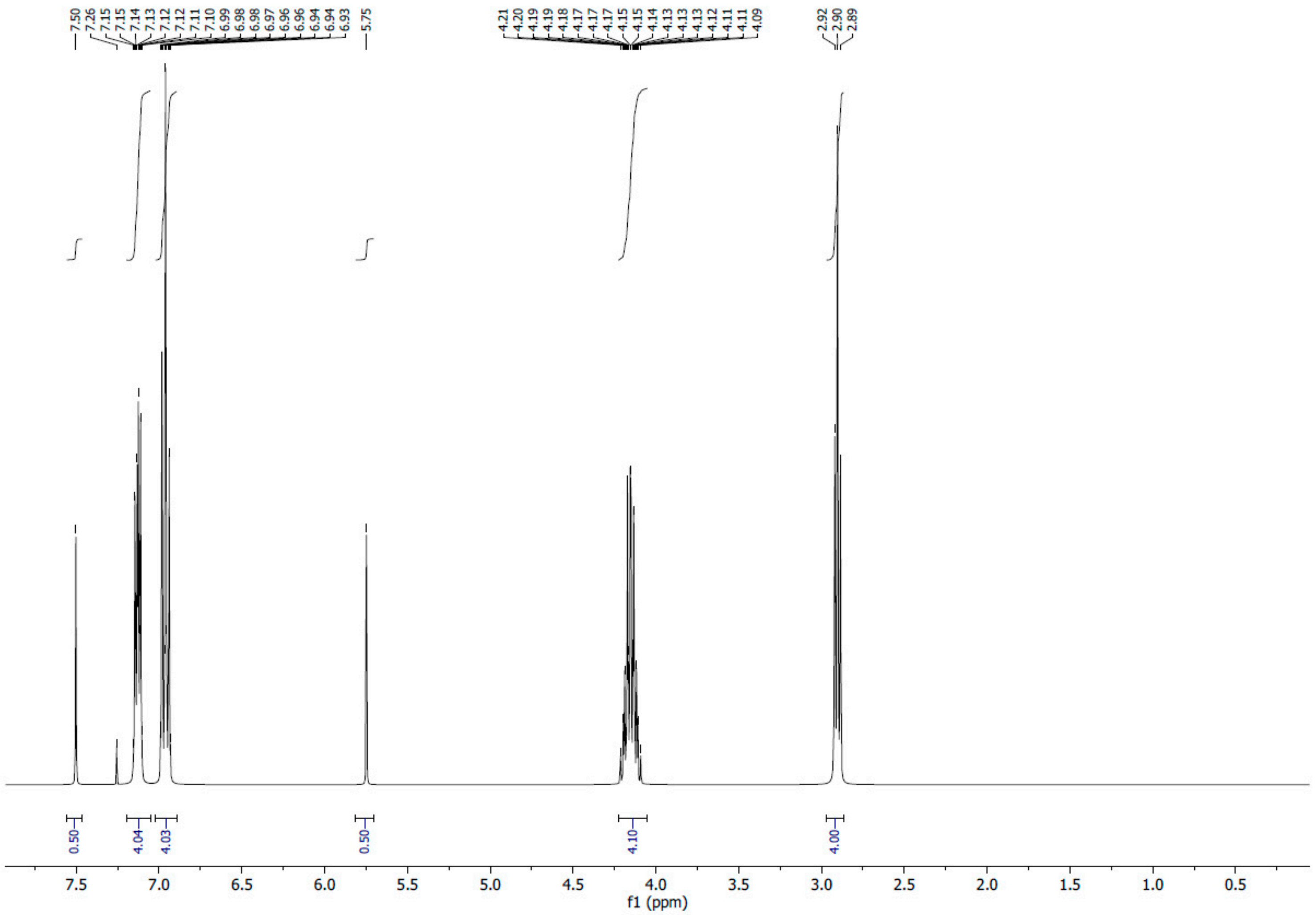

Figure S52. ^{1}HNMR (400 MHz, $CDCl_3$) spectra of bis(4-fluorophenylethyl)phosphite.

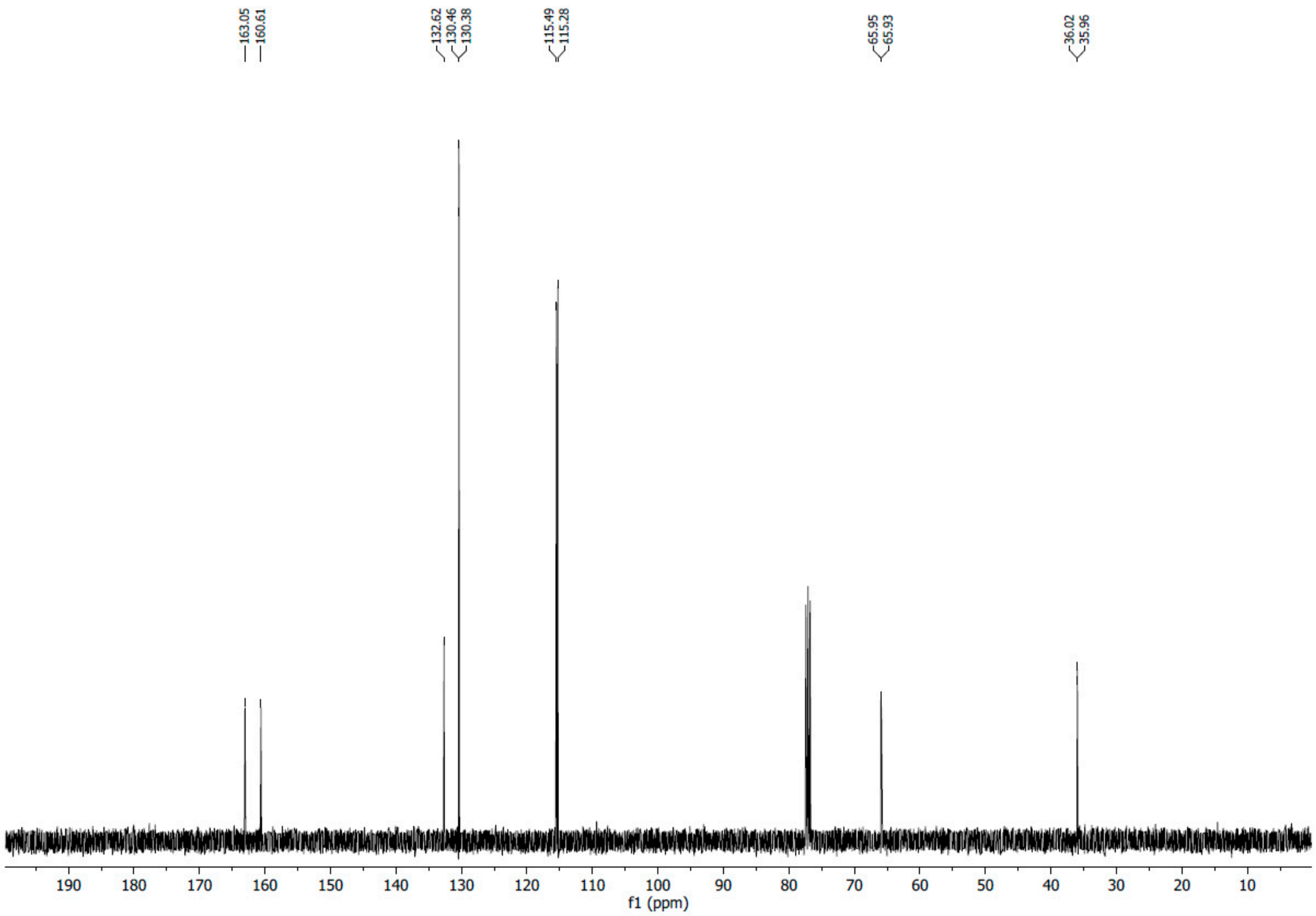

Figure S53. ^{13}CNMR (100 MHz, $CDCl_3$) spectra of bis(4-fluorophenylethyl)phosphite.

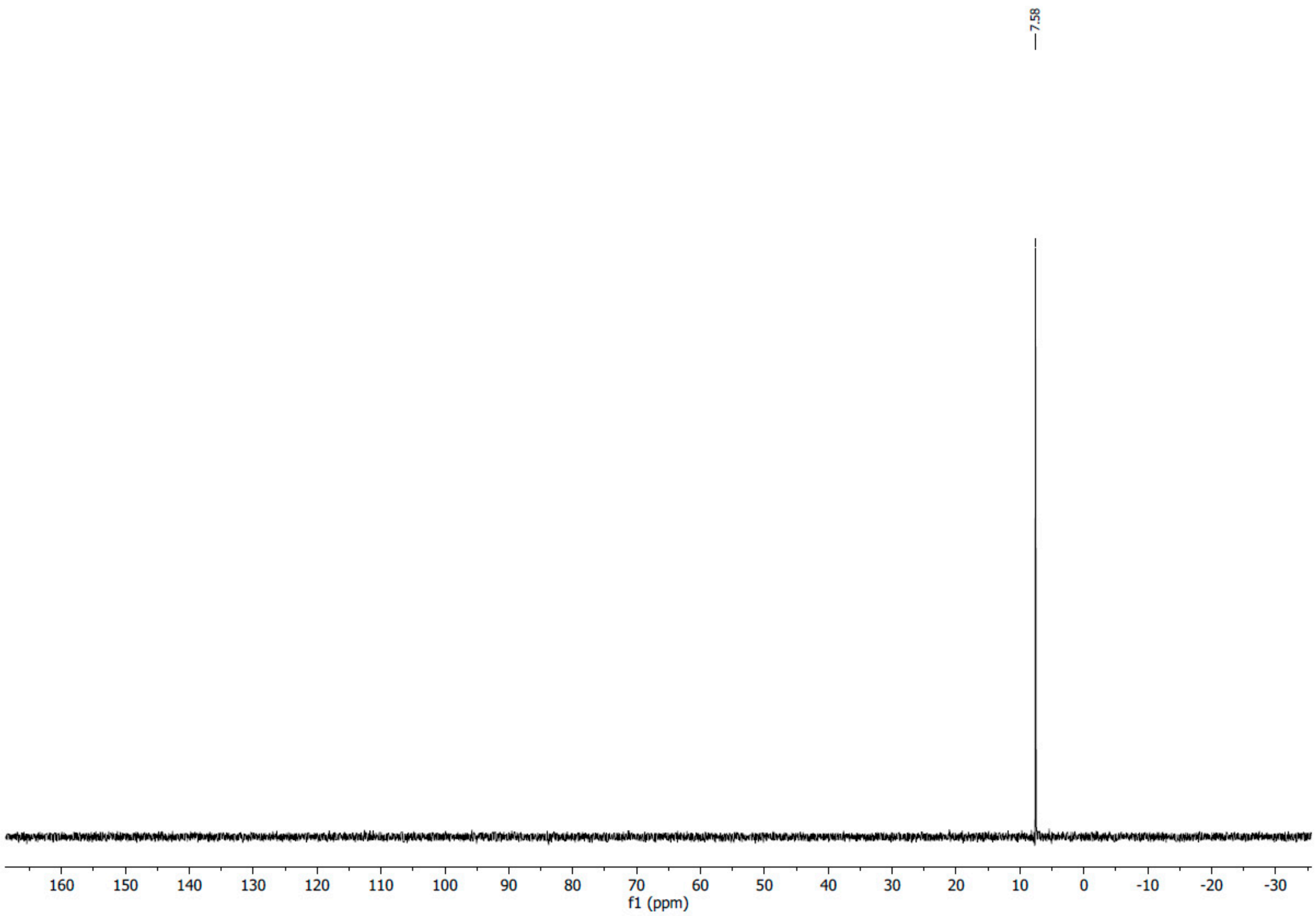

Figure S54. ^{31}PNMR (162 MHz, $CDCl_3$) spectra of bis(4-fluorophenylethyl)phosphite.

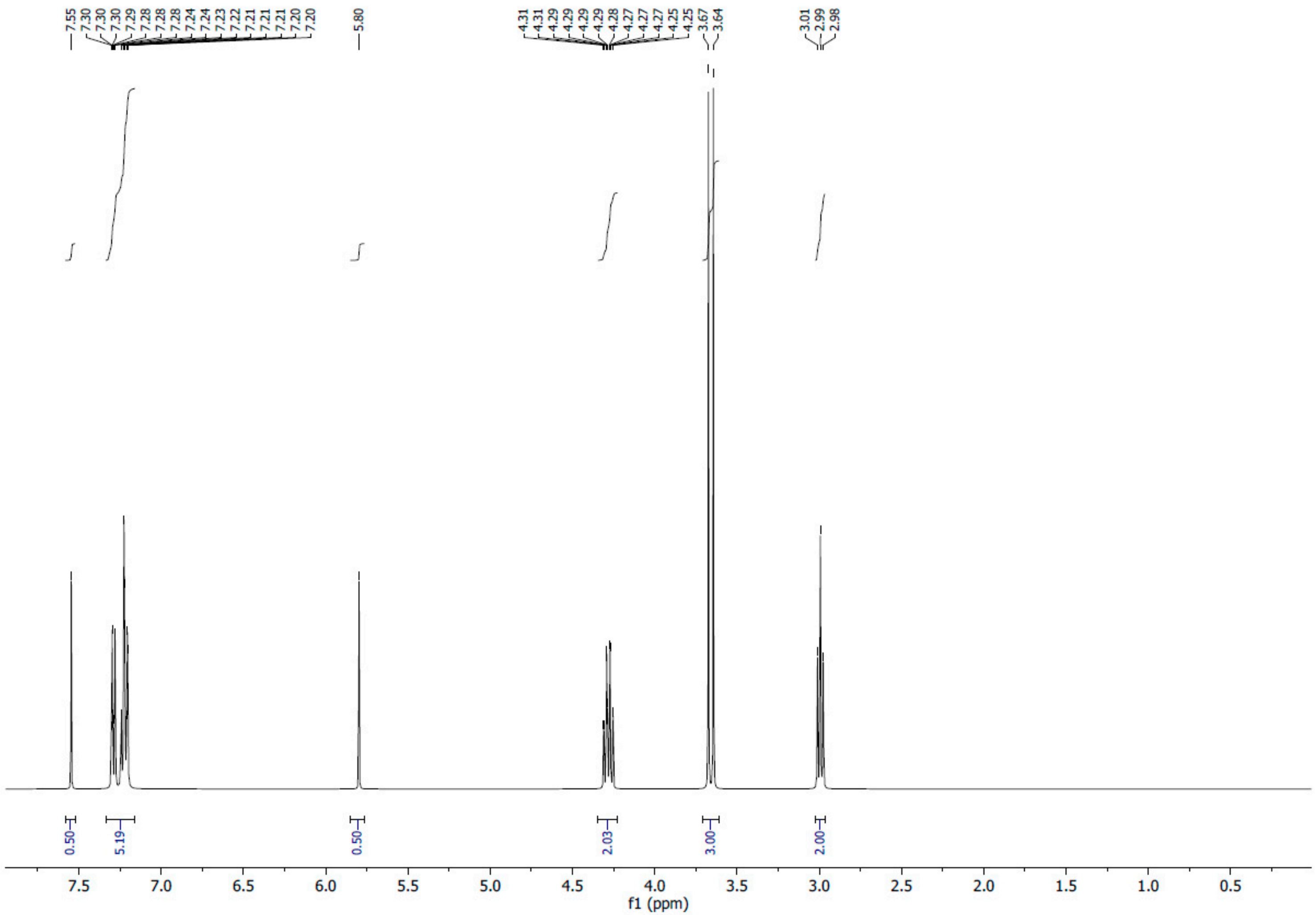

Figure S55. ^{1}HNMR (400 MHz, $CDCl_3$) spectra of methyl (phenylethyl) phosphite.

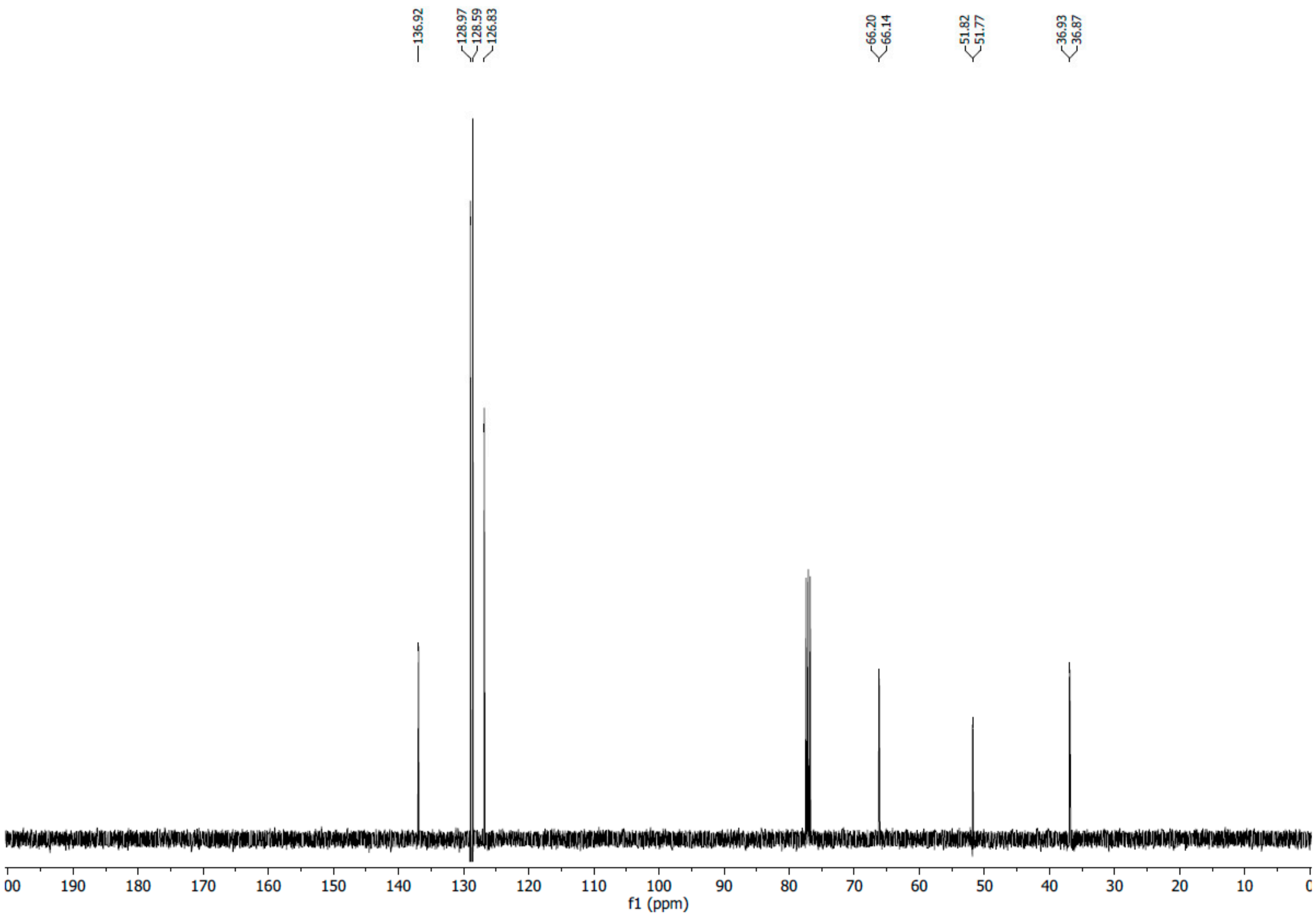

Figure S56. ^{13}CNMR (100 MHz, $CDCl_3$) spectra of methyl (phenylethyl) phosphite.

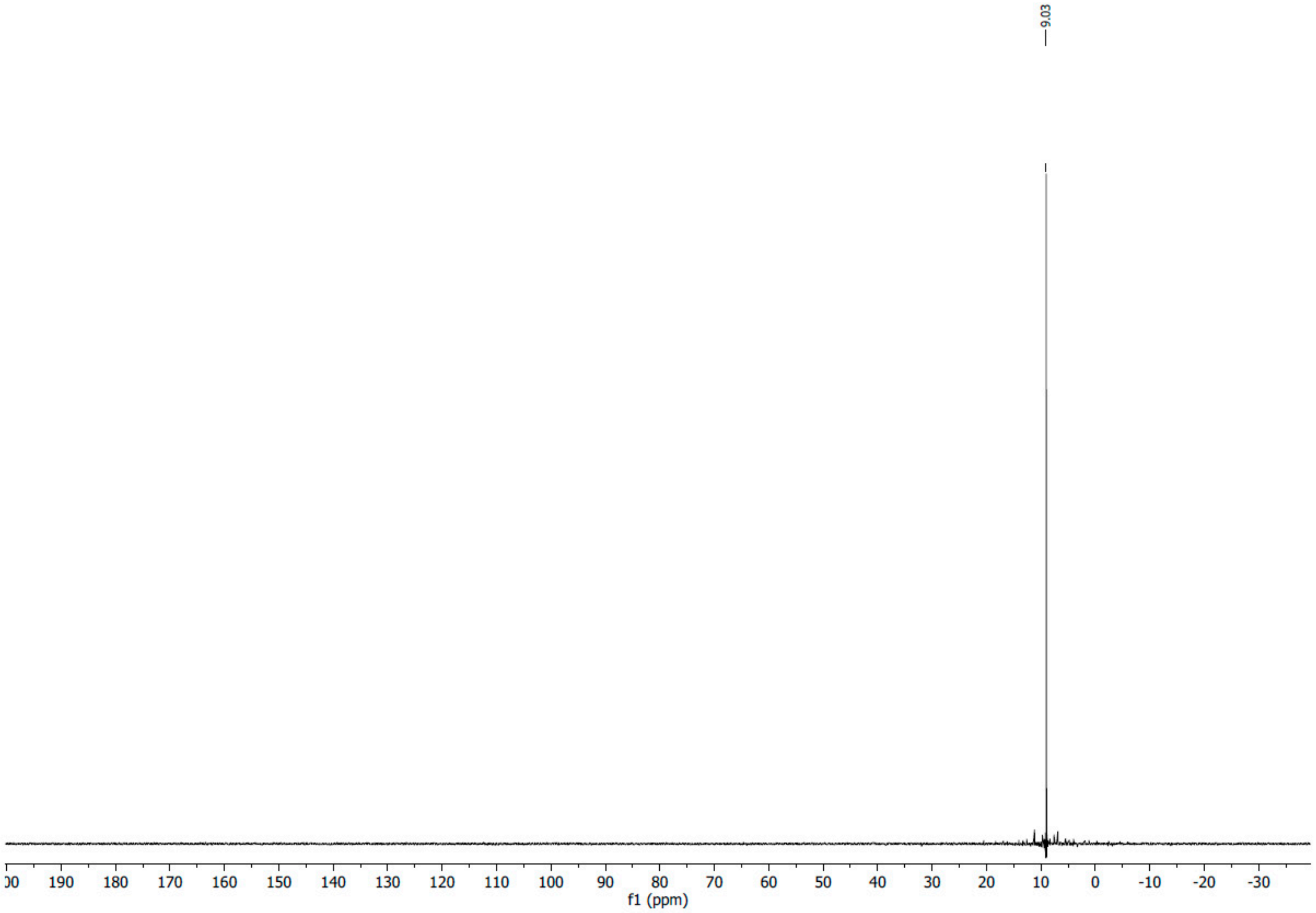

Figure S57. ^{31}PNMR (162 MHz, $CDCl_3$) spectra of methyl (phenylethyl) phosphite.

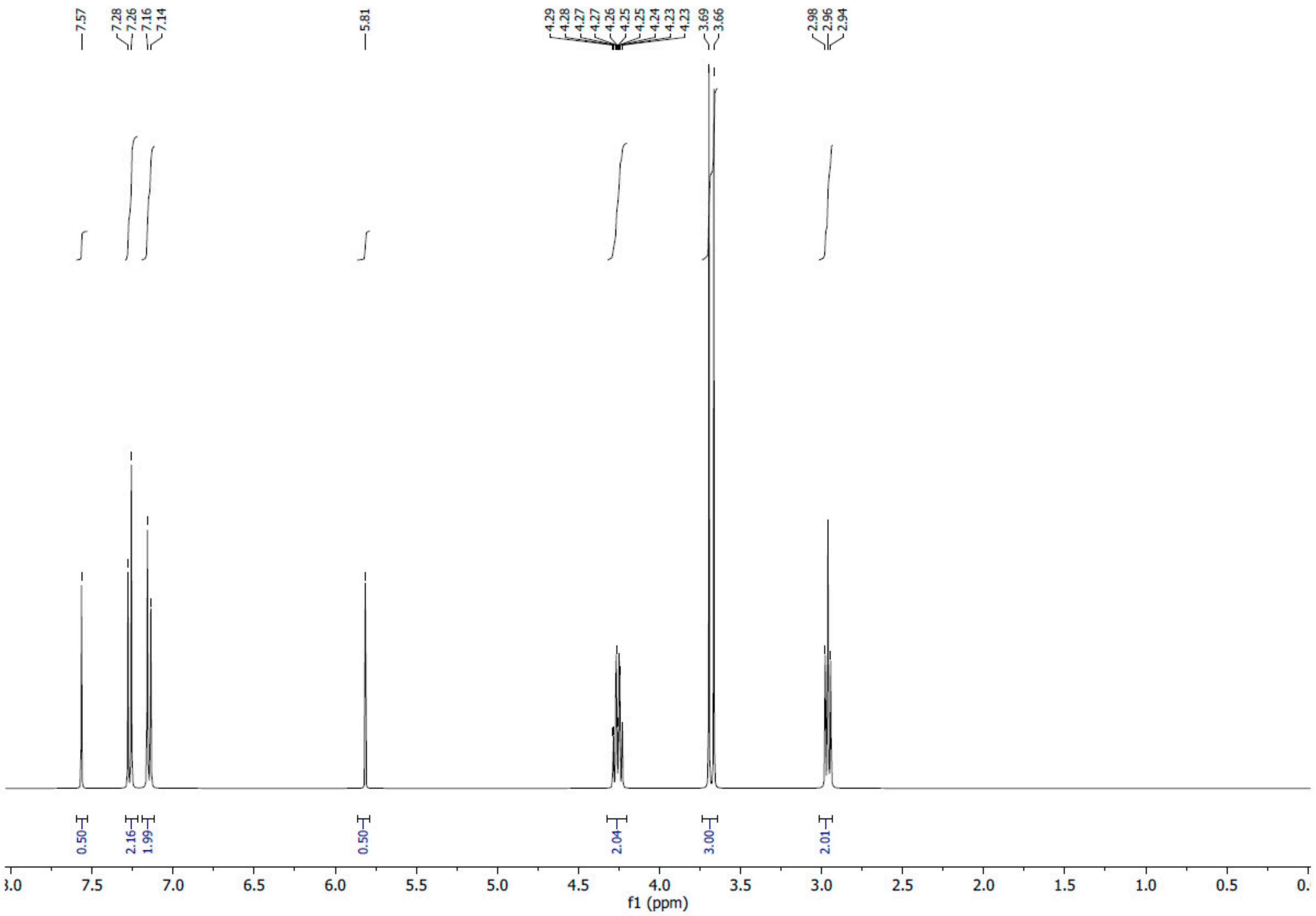

Figure S58. ^{1}HNMR (400 MHz, $CDCl_3$) spectra of methyl (4-chlorophenylethyl) phosphite.

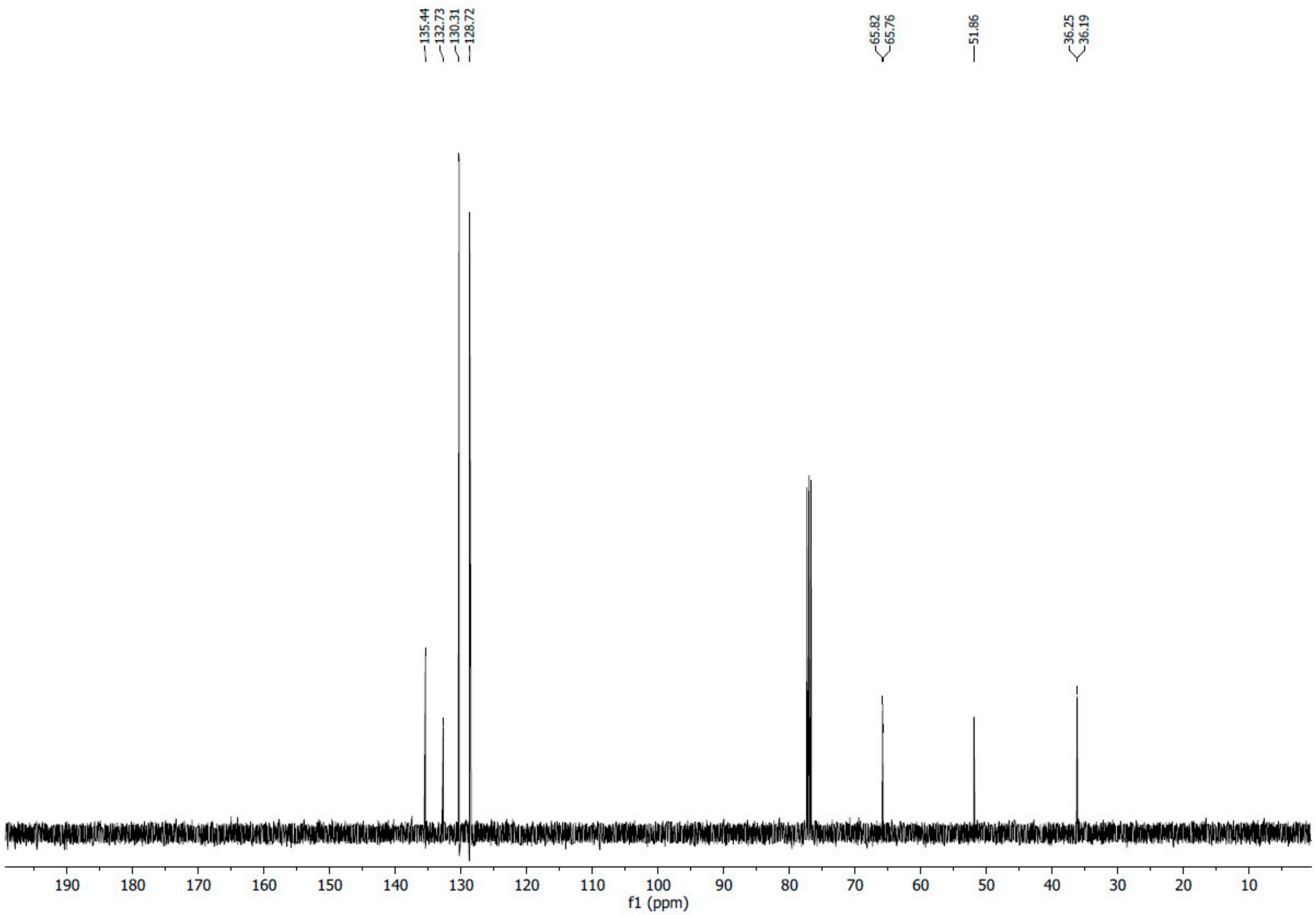

Figure S59. ^{13}CNMR (100 MHz, $CDCl_3$) spectra of methyl (4-chlorophenylethyl) phosphite.

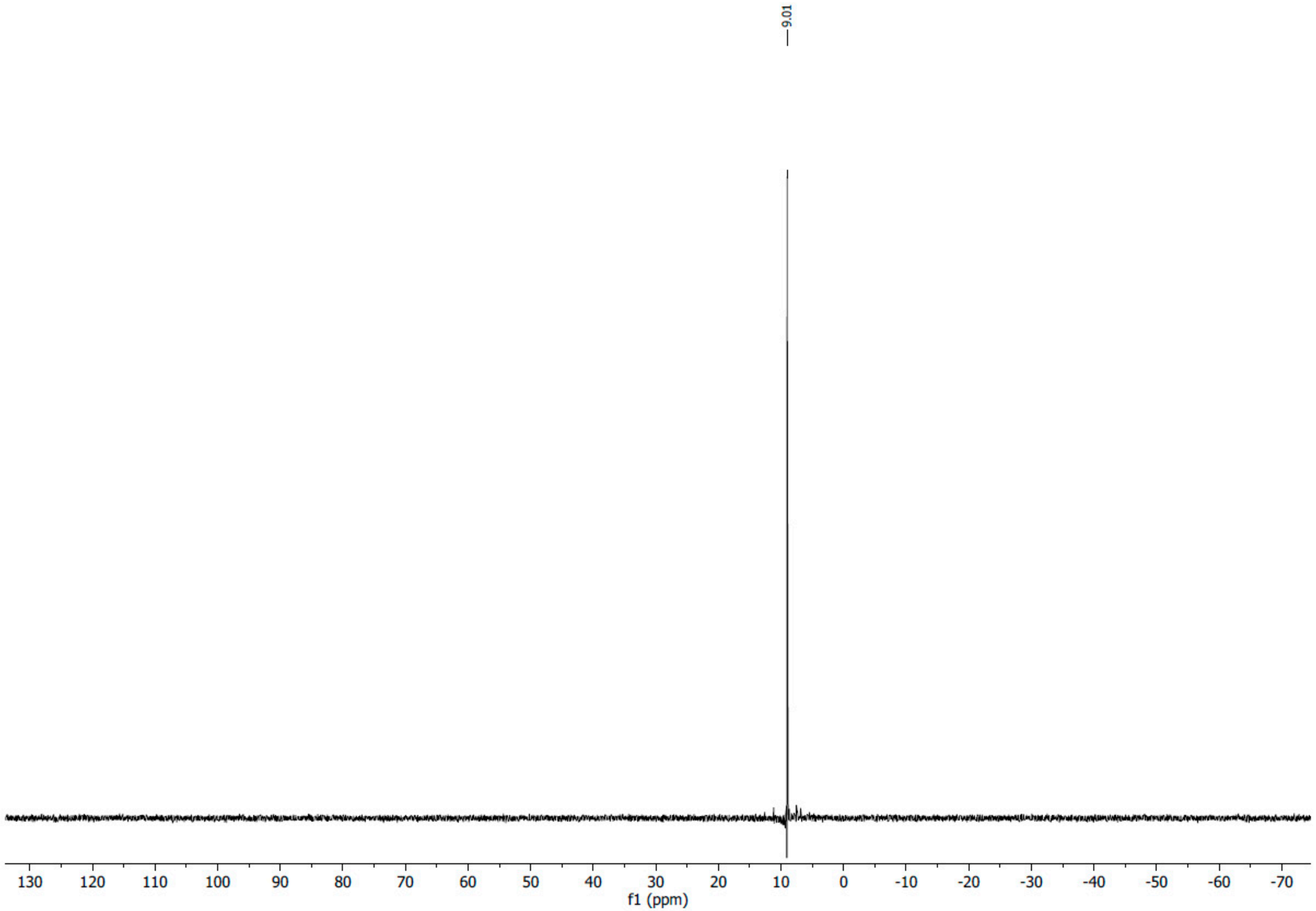

Figure S60. ^{31}PNMR (162 MHz, $CDCl_3$) spectra of methyl (4-chlorophenylethyl) phosphite.

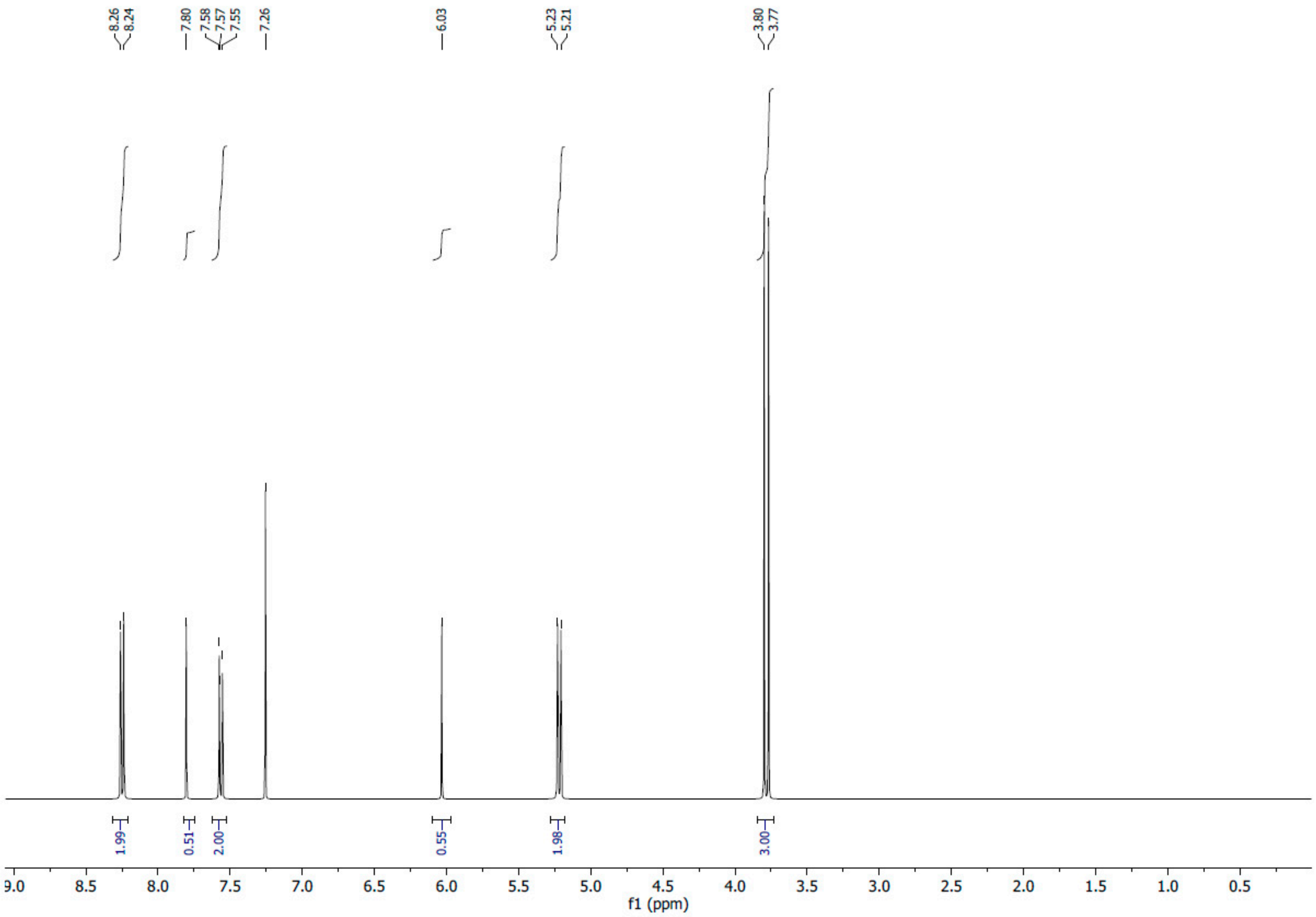

Figure S61. ^{1}HNMR (400 MHz, $CDCl_3$) spectra of methyl (4-nitrobenzyl) phosphite.

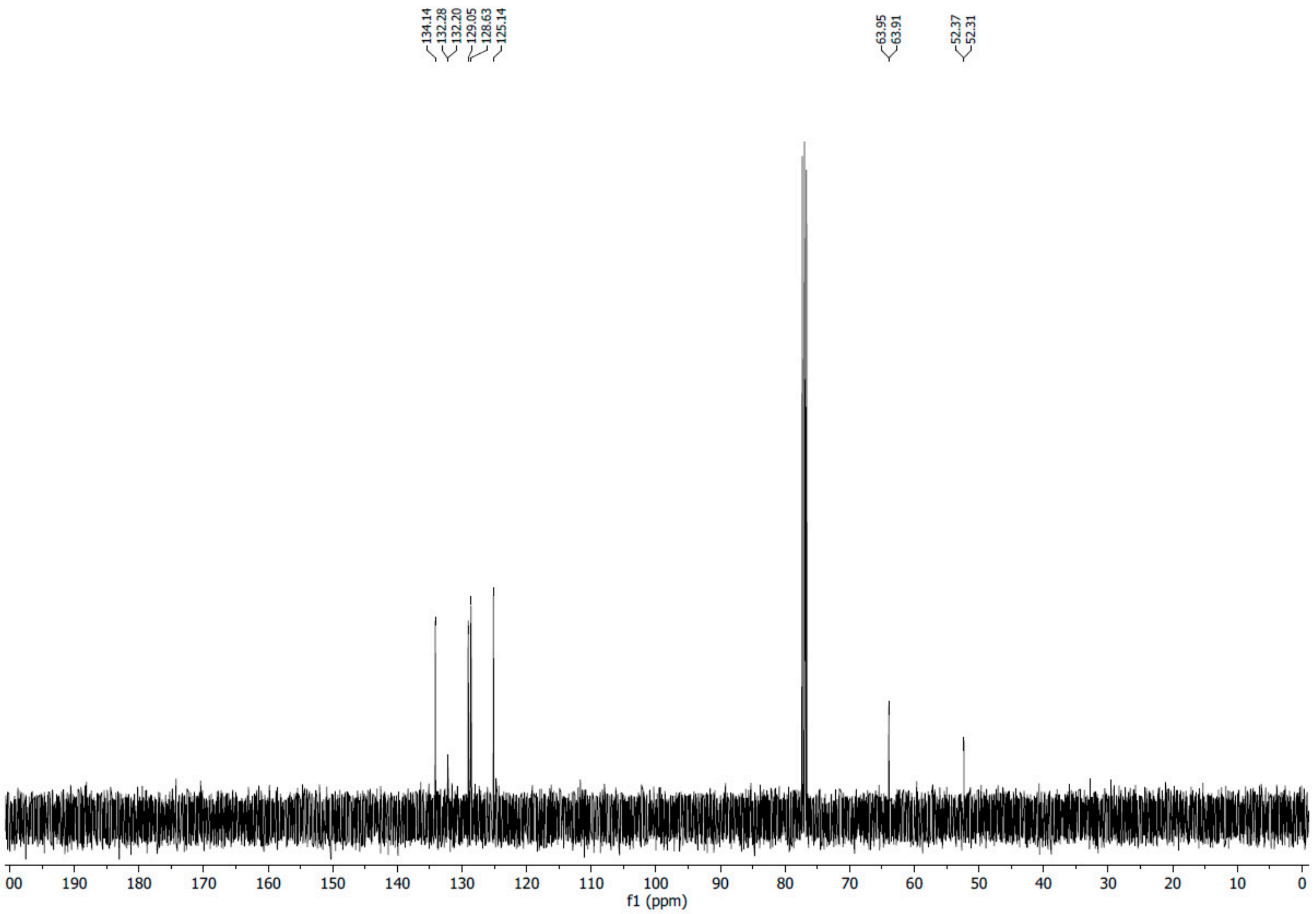

Figure S62. ^{13}CNMR (100 MHz, $CDCl_3$) spectra of methyl (4-nitrobenzyl) phosphite.

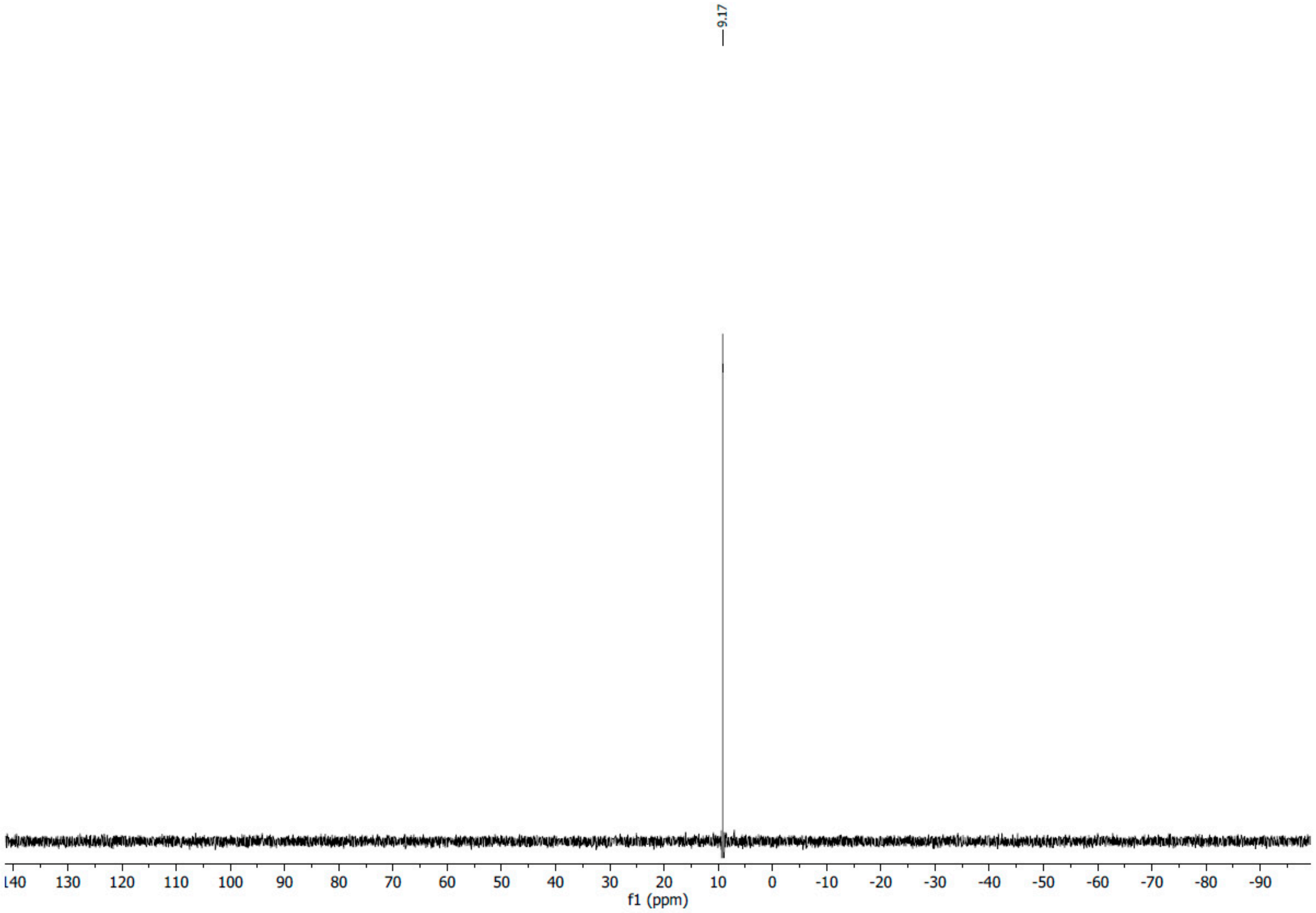

Figure S63. ^{31}PNMR (162 MHz, $CDCl_3$) spectra of methyl (4-nitrobenzyl) phosphite.

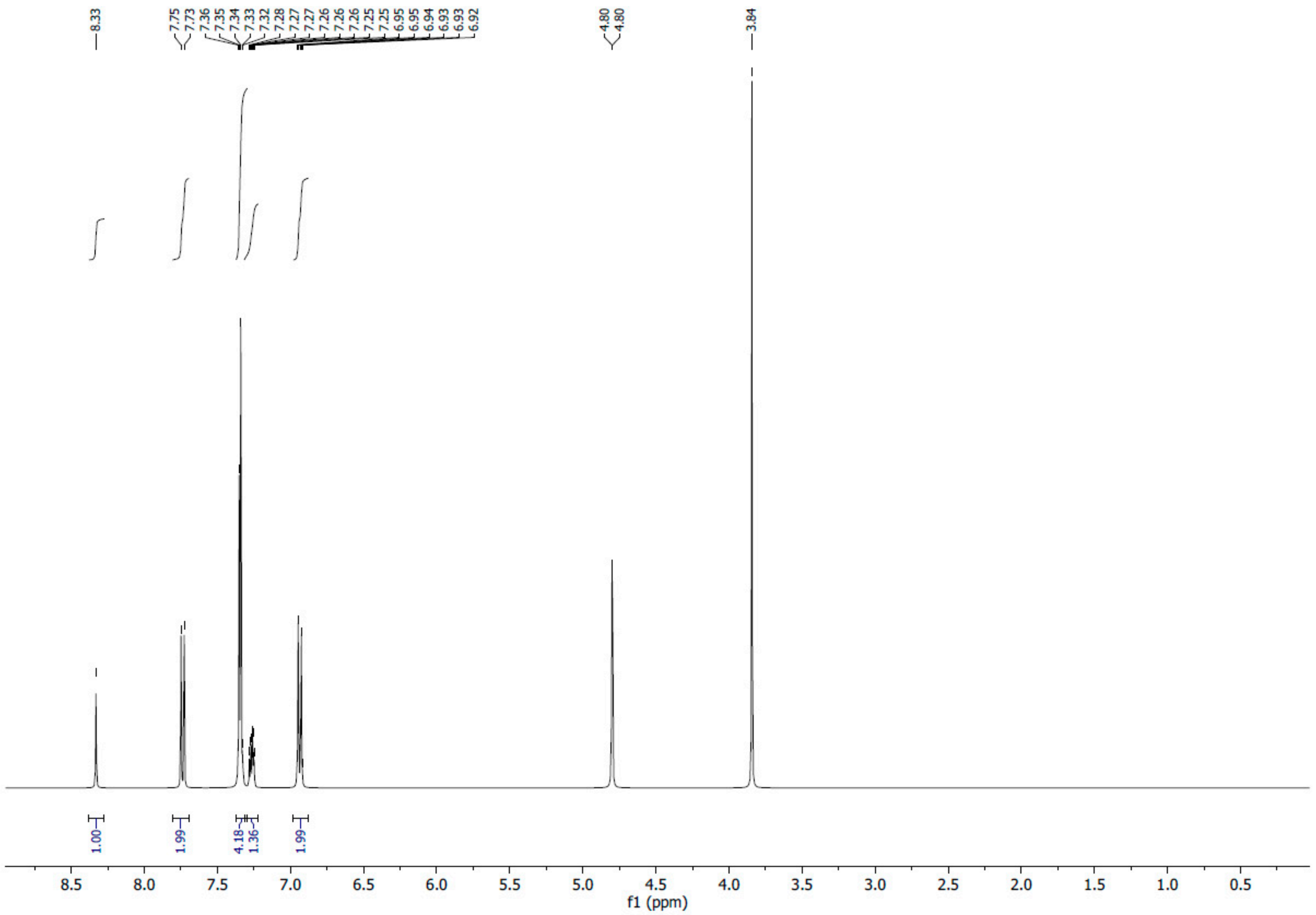

Figure S64. ^{1}HNMR (400 MHz, $CDCl_3$) spectra of *N*-(4-methoxylbenzylidene)benzylamine.

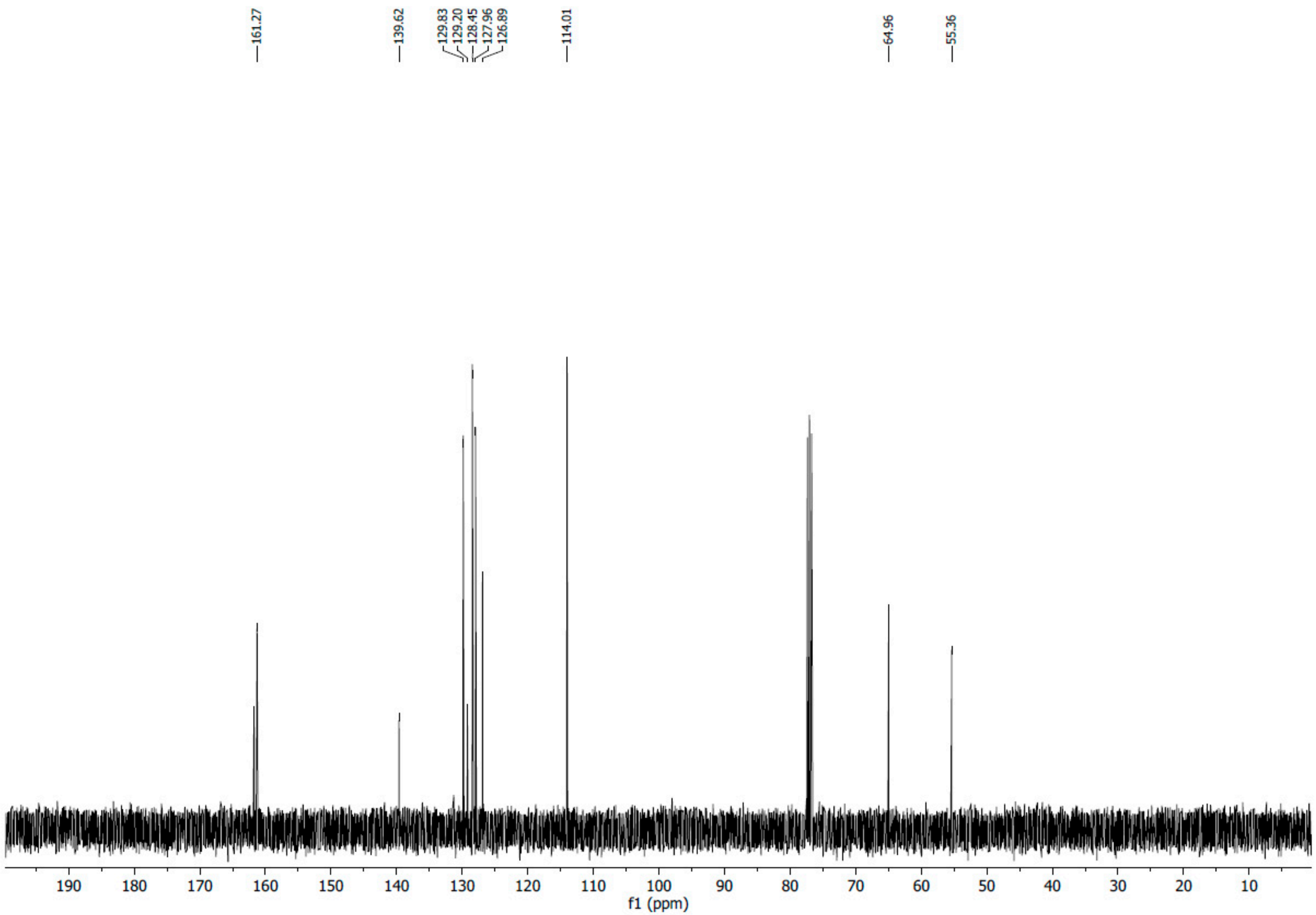

Figure S65. ^{13}CNMR (100 MHz, $CDCl_3$) spectra of *N*-(4-methoxylbenzylidene)benzylamine.

General methods of synthesis α-amino phosphonate derivatives

Bis(4-fluorophenylethyl)phosphite. ^{1}H NMR (400 MHz, $CDCl_3$) δ 7.20 – 7.05 (m, 4H), 7.02 – 6.89 (m, 4H), 5.75 (s, 1H), 4.23 – 4.05 (m, 4H), 2.90 (t, J = 6.8 Hz, 4H); ^{31}P NMR (162 MHz, $CDCl_3$) δ 7.58; ^{13}C NMR (100 MHz, $CDCl_3$) δ 163.0, 160.6, 132.6, 130.4, 130.3, 115.4, 115.2, 65.9, 36.0, 35.9. HRMS (EI) m/z calcd for $C_{16}H_{18}F_2O_3P$ [M+H]$^+$ 327.0956, found 327.0954.

Methyl (phenylethyl) phosphite. ^{1}H NMR (400 MHz, $CDCl_3$) δ 7.55 (s, 1H), 7.34 – 7.16 (m, 5H), 5.80 (s, 1H), 4.28 (dtd, *J* = 8.7, 6.9, 0.8 Hz, 2H), 3.66 (d, *J* = 12.0 Hz, 3H), 2.99 (t, *J* = 6.9 Hz, 2H); ^{31}P NMR (162 MHz, $CDCl_3$) δ 9.03; ^{13}C NMR (100 MHz, $CDCl_3$) δ 136.9, 128.9, 128.5, 126.8, 66.2, 66.1, 51.8, 51.7, 36.9, 36.8. HRMS (EI) m/z calcd for $C_8H_{11}O_3P$ [M+H]$^+$ 187.0518, found 187.0519.

(4-Chlorophenylethyl) methyl phosphite. ^{1}H NMR (400 MHz, $CDCl_3$) δ 7.57 (s, 1H), 7.27 (d, *J* = 8.5 Hz, 2H), 7.15 (d, *J* = 8.4 Hz, 2H), 5.81 (s, 1H), 4.26 (dtd, *J* = 8.1, 6.7, 1.4 Hz, 2H), 3.68 (d, *J* = 12.0 Hz, 3H), 2.96 (t, *J* = 6.8 Hz, 2H); ^{31}P NMR (162 MHz, $CDCl_3$) δ 9.01; ^{13}C NMR (100 MHz, $CDCl_3$) δ 135.4, 132.7, 130.3, 128.7, 65.8, 65.7, 51.8, 36.2, 36.1. HRMS (EI) m/z calcd for $C_9H_{12}ClO_3P$ [M+H]$^+$ 235.0284, found 235.0281.

Methyl (4-nitrobenzyl) phosphite. ^{1}H NMR (400 MHz, $CDCl_3$) δ 8.25 (d, *J* = 8.8 Hz, 2H), 7.80 (s, 1H), 7.63 – 7.52 (m, 2H), 6.03 (s, 1H), 5.22 (d, *J* = 9.3 Hz, 2H), 3.78 (d, *J* = 12.0 Hz, 3H); ^{31}P NMR (162 MHz, $CDCl_3$) δ 9.17; ^{13}C NMR (100 MHz, $CDCl_3$) δ 134.1, 132.2, 132.20 129.0, 128.6, 125.1, 63.9, 63.9, 52.3, 52.3. HRMS (EI) m/z calcd for $C_8H_{10}NO_5P$ [M+H]$^+$ 232.0369, found 232.0366.

*2.4.1. General procedure for the synthesis of α-amino phosphonate derivatives **1–16**.*

General procedure for enzyme catalysed Kabachnik-Fields reaction. A mixture of the corresponding aldehyde (1 mmol), amine (1 mmol), and *H*-phosphite in TBME (2 mL) (1 mmol) was shaken at 200 rpm with porcine pancreas lipase (PPL) (50 mg) at 30 °C for 24 hours. After the completion of reaction. the catalyst was separated on a glass frit funnel. The residue was washed with ethyl acetate. The combined organic phase was concentrated under vacuum. The resulting residue was purified by column chromatography (silica gel, eluent: ethyl acetate/hexanes, 7:3) to afford the target α-amino phosphonate derivatives **1-16**. The yields of the obtained derivatives are shown in Figure 2. The structure of all new compounds were confirmed using NMR and mass spectroscopy.

Dimethyl ((benzylamino)(phenyl)methyl)phosphonate (1). Compound **1** was obtained according to the General procedure as colorless oil with 91% yield (278 mg, 0.91 mmol); ^{1}H NMR (400 MHz, $CDCl_3$) δ 7.47 – 7.19 (m, 10H), 4.05 (d, *J* = 20.2 Hz, 1H), 3.79 (d, *J* = 13.3 Hz, 1H), 3.72 (d, *J* = 10.5 Hz, 3H), 3.53 (dd, *J* = 11.8, 6.4 Hz, 4H), 2.58 (s, 1H); ^{31}P NMR (162 MHz, $CDCl_3$) δ 25.78; ^{13}C NMR (100 MHz, $CDCl_3$) δ 139.2, 135.5 (d, *J* = 2.9 Hz), 128.6 (d, *J* = 5.8 Hz), 128.6 (d, *J* = 6.6 Hz), 128.3 (d, *J* = 2.9 Hz), 127.1, 60.0, 58.5 (d, *J* = 154.2 Hz), 53.7 (d, *J* = 7.1 Hz), 53.4(d, *J* = 7.1 Hz), 51.1 (d, *J* = 17.5 Hz). NMR data were in accordance with those reported in the literature [41].

Dimethyl ((benzylamino)(4-fluorophenyl)methyl)phosphonate (2). Compound **2** was obtained according to the General procedure as colorless oil with 84% yield (271 mg, 0.84 mmol); ^{1}H NMR (400 MHz, $CDCl_3$) δ 7.38 (ddd, *J* = 8.8, 5.3, 2.3 Hz, 2H), 7.33 – 7.26 (m, 2H), 7.25 – 7.15 (m, 3H), 7.05 (td, *J* = 8.7, 0.9 Hz, 2H), 4.02 (d, *J* = 19.8 Hz, 1H), 3.77 (d, *J* = 13.3 Hz, 1H), 3.71 (d, *J* = 10.5 Hz, 3H), 3.54 (d, *J* = 10.5 Hz, 3H), 3.50 (d, *J* = 13.4 Hz, 1H), 2.33 (s, 1H); ^{31}P NMR (162 MHz, $CDCl_3$) δ 25.53; ^{13}C NMR (101 MHz, $CDCl_3$) δ 163.7, 161.3, 161.2,

139.0, 131.2, 131.2, 131.2, 130.2, 130.2, 130.2, 130.1, 128.4, 128.2, 127.2, 115.6, 115.6, 115.4, 59.2, 57.7, 53.7, 53.6, 53.4, 53.3, 51.1, 51.0. NMR data were in accordance with those reported in the literature [42].

Dimethyl ((benzylamino)(4-methoxyphenyl)methyl)phosphonate (3). Compound **3** was obtained according to the General procedure as colorless oil with 87% yield (292 mg, 0.87 mmol); ^{1}H NMR (400 MHz, $CDCl_3$) δ 7.39 – 7.19 (m, 7H), 6.96 – 6.84 (m, 2H), 3.99 (d, *J* = 19.6 Hz, 1H), 3.81 (s, 3H), 3.77 (s, 1H), 3.72 (d, *J* = 10.5 Hz, 3H), 3.55 (d, *J* = 3.2 Hz, 2H), 3.52 (d, *J* = 6.1 Hz, 2H), 2.28 (s, 1H); ^{31}P NMR (162 MHz, $CDCl_3$) δ 26.10; ^{13}C NMR (101 MHz, $CDCl_3$) δ 159.4, 139.2, 129.7, 129.6, 128.3, 127.2, 127.1, 114.0, 59.3, 57.7, 55.2, 53.7, 53.4, 53.3, 51.0, 50.9. NMR data were in accordance with those reported in the literature [43].

Dimethyl [1-(benzylamino)butyl]phosphonate (4). Compound **4** was obtained according to the General procedure as colorless oil with 71% yield (193 mg, 0.71 mmol); ^{1}H NMR (400 MHz, $CDCl_3$) δ 7.33 – 7.17 (m, 5H), 3.98 – 3.81 (m, 2H), 3.76 (d, *J* = 9.1 Hz, 3H), 3.73 (d, *J* = 9.2 Hz, 3H), 2.87 (ddd, *J* = 12.1, 8.5, 4.7 Hz, 1H), 1.77 – 1.65 (m, 1H), 1.65 – 1.46 (m, 3H), 1.43 – 1.28 (m, 1H), 0.85 (t, *J* = 7.2 Hz, 3H); ^{31}P NMR (162 MHz, $CDCl_3$) δ 28.30; ^{13}C NMR (100 MHz, $CDCl_3$) δ 140.0, 128.3, 128.2, 127.0, 54.2, 52.5, 52.2 (d, *J* = 6.4 Hz), 31.9, 19.3 (d, *J* = 10.9 Hz), 13.8. NMR data were in accordance with those reported in the literature [44].

Dimethyl [1-(benzylamino)-3-methylbutyl]phosphonate (5). Compound **5** was obtained according to the General procedure as colorless oil with 63% yield (180 mg, 0.63 mmol); ^{1}H NMR (400 MHz, $CDCl_3$) δ 7.38 – 7.22 (m, 5H), 3.99 (dd, *J* = 13.0, 1.1 Hz, 1H), 3.85 (dd, *J* = 13.1, 2.1 Hz, 1H), 3.80 (d, *J* = 5.5 Hz, 3H), 3.77 (d, *J* = 5.5 Hz, 3H), 3.05 – 2.92 (m, 1H), 1.95 – 1.84 (m, 2H), 1.57 – 1.45 (m, 2H), 0.90 (d, *J* = 6.7 Hz, 3H), 0.75 (d, *J* = 6.6 Hz, 3H); ^{31}P NMR (162 MHz, $CDCl_3$) δ 31.56; ^{13}C NMR (100 MHz, $CDCl_3$) δ 140.0, 128.3, 128.2, 127.0, 52.8 (d, *J* = 7.3 Hz), 52.6 (d, *J* = 6.5 Hz), 52.5 (d, *J* = 3.7 Hz), 52.2 (d, *J* = 147.9 Hz), 39.1 (d, *J* = 2.2 Hz), 24.4 (d, *J* = 11.7 Hz), 23.3, 21.2. NMR data were in accordance with those reported in the literature [45].

Dimethyl [1-(4-methoxybenzylamino)phenyl]phosphonate (6). Compound **6** was obtained according to the General procedure as colorless oil with 93% yield (312 mg, 0.93 mmol); ^{1}H NMR (400 MHz, $CDCl_3$) δ 7.43 – 7.28 (m, 5H), 7.15 (d, *J* = 8.8 Hz, 2H), 6.83 (d, *J* = 8.7 Hz, 2H), 4.02 (d, *J* = 20.3 Hz, 1H), 3.77 (s, 3H), 3.72 (d, *J* = 13.1 Hz, 4H), 3.71 (d, *J* = 10.4 Hz, 3H), 3.52 (d, *J* = 10.4 Hz, 3H), 3.47 (d, *J* = 13.1 Hz, 1H); ^{31}P NMR (162 MHz, $CDCl_3$) δ 25.88; ^{13}C NMR (100 MHz, $CDCl_3$) δ 158.8, 135.5, 131.2, 129.5, 128.6, 128.5, 128.0, 127.9, 113.7, 59.8, 58.3, 55.2, 53.7, 53.4, 50.5, 50.4. HRMS (ESI) m/z calcd for $C_{17}H_{22}NO_4NaP$ $[M+Na]^+$ 334.1208, found 334.1206.

Dimethyl [1-(4-methoxybenzylamino)-4-methoxyphenyl]phosphonate (7). Compound **7** was obtained according to the General procedure as colorless oil with 76% yield (278 mg, 0.76 mmol); ^{1}H NMR (400 MHz, $CDCl_3$) δ 7.34 – 7.26 (m, 2H), 7.13 (d, *J* = 8.7 Hz, 2H), 6.88 (dd, *J* = 8.9, 0.8 Hz, 2H), 6.81 (d, *J* = 8.7 Hz, 2H), 3.95 (d, *J* = 19.6 Hz, 1H), 3.78 (s, 3H), 3.75 (s, 3H), 3.73 – 3.66 (m, 4H), 3.50 (s, 3H), 3.44 (d, *J* = 13.0 Hz, 1H), 2.43 (s, 1H); ^{31}P NMR (162 MHz, $CDCl_3$) δ 26.18; ^{13}C NMR (100 MHz, $CDCl_3$) δ 159.3, 158.7, 131.2, 129.7, 129.6, 129.5, 127.3, 127.2, 114.0, 113.7, 59.0, 57.5, 55.2, 55.1, 53.6, 53.5, 53.3, 50.4, 50.2. HRMS (ESI) m/z calcd for $C_{18}H_{23}NO_5P$ $[M-H]^-$ 364.1314, found 364.1312.

Dimethyl [1-(4-methoxybenzylamino)-2,4-dimethoxyphenyl]phosphonate (8). Compound **8** was obtained according to the General procedure as colorless oil with 69% yield (294 mg, 0.69 mmol); ^{1}H NMR (400 MHz, $CDCl_3$) δ 7.44 (dd, *J* = 8.5, 2.6 Hz, 1H), 6.94 (d, *J* = 8.8 Hz, 1H), 6.48 (dd, *J* = 8.5, 2.4 Hz, 1H), 6.38 (dd, *J* = 2.5, 1.1 Hz, 1H), 6.31 (d, *J* = 7.1 Hz, 2H), 4.52 (d, *J* = 19.7 Hz, 1H), 3.74 (s, 3H), 3.72 – 3.70 (m, 6H), 3.69 (s, 2H), 3.68 (s, 3H), 3.65 (d, *J* = 14.5 Hz, 3H), 3.48 (d, *J* = 10.4 Hz, 3H), 3.42 (d, *J* = 13.2 Hz, 1H), 2.49 (s, 1H); ^{31}P NMR (162 MHz, $CDCl_3$) δ 27.12; ^{13}C NMR (100 MHz, $CDCl_3$) δ 160.3, 160.2, 160.0, 158.6, 158.5, 130.5, 129.6, 120.3, 116.5, 104.7, 104.7, 103.5, 98.2, 55.5, 55.2, 55.0, 53.4, 53.4, 53.1, 53.0, 51.6, 50.0, 46.7, 46.5, 14.1. HRMS (ESI) m/z calcd for $C_{20}H_{29}NO_7P$ $[M+H]^+$ 426.1682, found 426.1684.

Dimethyl [1-(2-phenylethyl)amino)-2,4-dimethoxyphenyl]phosphonate (9). Compound **9** was obtained according to the General procedure as colorless oil with 51% yield (178 mg, 0.51 mmol); ^{1}H NMR (400 MHz, $CDCl_3$) δ 7.29 – 7.22 (m, 4H), 7.22 – 7.07 (m, 3H), 6.86 (d, *J* = 8.0 Hz, 2H), 4.00 (d, *J* = 19.6 Hz, 1H), 3.79 (s, 3H), 3.68 (d, *J* = 10.5 Hz, 3H), 3.52 (d, *J* = 10.4 Hz, 3H), 2.84 – 2.67 (m, 4H), 1.88 (s, 1H); ^{31}P NMR (162 MHz, $CDCl_3$) δ 25.90; ^{13}C NMR (100 MHz, $CDCl_3$) δ 159.3, 139.7, 129.5, 129.4, 128.6, 128.3, 127.5, 127.4, 126.1, 126.1, 114.0, 113.9, 60.7, 59.2, 55.2, 53.6, 53.5, 53.4, 53.3, 49.0, 48.9, 36.1. HRMS (ESI) m/z calcd for $C_{18}H_{24}NO_4NaP$ $[M+H]^+$ 372.1341, found 372.1343.

Dimethyl [1-(2-phenylethyl)amino)-phenyl]phosphonate (10). Compound **10** was obtained according to the General procedure as colorless oil with 55% yield (176 mg, 0.55 mmol); ^{1}H NMR (400 MHz, $CDCl_3$) δ 7.37 – 7.18 (m, 7H), 7.19 – 7.05 (m, 3H), 4.05 (d, *J* = 20.2 Hz, 1H), 3.66 (d, *J* = 10.5 Hz, 3H), 3.48 (d, *J* = 10.5 Hz, 3H), 2.87 – 2.67 (m, 4H); ^{31}P NMR (162 MHz, $CDCl_3$) δ 25.56; ^{13}C NMR (100 MHz, $CDCl_3$) δ 139.6, 135.7, 128.6, 128.5, 128.4, 128.3, 127.9, 126.1, 61.4, 59.9, 53.6, 53.5, 53.4, 53.3, 49.1, 49.0, 36.1. HRMS (ESI) m/z calcd for $C_{17}H_{22}NO_3NaP$ $[M+H]^+$ 342.1235, found 342.1236.

Dimethyl [1-butylamino)-4-methoxyphenyl]phosphonate (11). Compound **11** was obtained according to the General procedure as colorless oil with 69% yield (208 mg, 0.69 mmol); ^{1}H NMR (400 MHz, , $CDCl_3$) δ 7.34 – 7.24 (m, 2H), 6.90 – 6.78 (m, 2H), 3.95 (d, *J* = 19.5 Hz, 1H), 3.75 (s, 3H), 3.68 (d, *J* = 10.5 Hz, 3H), 3.51 (d, *J* = 10.4 Hz, 3H), 2.52 – 2.34 (m, 2H), 1.47 – 1.34 (m, 2H), 1.34 – 1.19 (m, 2H), 0.81 (t, *J* = 7.3 Hz, 3H); ^{31}P NMR (162 MHz, $CDCl_3$) δ 26.14; ^{13}C NMR (100 MHz, $CDCl_3$) δ 159.3, 159.2, 129.4, 113.9, 60.8, 59.2, 55.1, 53.5, 53.3, 47.5, 47.3, 31.9, 20.2, 13.8. HRMS (ESI) m/z calcd for $C_{14}H_{23}NO_4P$ $[M+Na]^+$ 300.1365, found 300.1369.

Dimethyl (phenyl)(4-fluorophenylamino)methylphosphonate (12). Compound **12** was obtained according to the General procedure as colorless oil with 82% yield (254 mg, 0.82 mmol); ^{1}H NMR (400 MHz, $CDCl_3$) δ 7.49 – 7.43 (m, 2H), 7.38 – 7.30 (m, 2H), 7.27 (dt, *J* = 8.5, 2.2 Hz, 1H), 6.79 (t, *J* = 8.7 Hz, 2H), 6.57 – 6.48 (m, 2H), 4.73 (d, *J* = 24.3 Hz, 1H), 3.75 (d, *J* = 10.7 Hz, 3H), 3.46 (d, *J* = 10.6 Hz, 3H); ^{31}P NMR (202 MHz, $CDCl_3$) δ 24.92; ^{13}C NMR (126 MHz, $CDCl_3$) δ 157.1, 155.3, 142.3, 142.2, 135.3, 128.6, 128.0, 127.7, 115.6, 115.4, 114.8, 114.7, 56.8, 55.6, 53.7, 53.6. NMR data were in accordance with those reported in the literature [46].

Di-2-(4-fluorophenyl)ethyl methyl (phenyl)(phenylamino)methylphosphonate (13). Compound **13** was obtained according to the General procedure as colorless oil with 79% yield (401 mg, 0.79 mmol); ^{1}H NMR (400 MHz, , $CDCl_3$) δ 7.42 – 7.35 (m, 2H), 7.34 – 7.20 (m, 3H), 7.18 – 7.02 (m, 4H), 7.02 – 6.84 (m, 6H), 6.71 (tt, *J* = 7.4, 1.1 Hz, 1H), 6.59 – 6.50 (m, 2H), 4.69 (d, *J* = 24.3 Hz, 1H), 4.13 (q, *J* = 7.0

Hz, 2H), 3.96 (dq, *J* = 10.1, 6.9 Hz, 1H), 3.75 (ddt, *J* = 10.1, 7.5, 6.7 Hz, 1H), 2.84 (t, *J* = 6.8 Hz, 2H), 2.68 (td, *J* = 6.9, 1.7 Hz, 2H); ^{31}P NMR (162 MHz, $CDCl_3$) δ 22.69; ^{13}C NMR (100 MHz, $CDCl_3$) δ 162.9, 160.5, 146.2, 146.0, 135.6, 132.8, 132.7, 130.4, 130.3, 129.2, 128.6, 128.0, 127.8, 118.6, 115.4, 115.3, 115.1, 113.9, 67.2, 67.2, 67.1, 67.0, 56.7, 55.2, 36.0, 35.8, 35.8. HRMS (ESI) m/z calcd for $C_{29}H_{28}NO_3NaF_2P$ $[M+Na]^+$ 530.1673, found 530.1676.

2-(Phenyl)ethyl methyl (phenyl)(phenylamino)methylphosphonate (14). Compound **14** was obtained as a mixture of diasteroisomers (ratio: 1:1) according to the General procedure as colorless oil with 63% yield (240 mg, 0.63 mmol); ^{1}H NMR (400 MHz, $CDCl_3$) δ 7.49 – 7.40 (m, 4H), 7.39 – 7.17 (m, 14H), 7.16 – 6.98 (m, 6H), 6.76 – 6.67 (m, 2H), 6.58 (dd, *J* = 7.5, 1.2 Hz, 4H), 4.76 (d, *J* = 24.3 Hz, 2H), 4.35 – 4.19 (m, 2H), 4.11 (dq, *J* = 10.1, 6.9 Hz, 1H), 3.92 – 3.79 (m, 1H), 3.66 (d, *J* = 10.7 Hz, 3H), 3.43 (d, *J* = 10.6 Hz, 2H), 2.95 (t, *J* = 6.9 Hz, 2H), 2.78 (td, *J* = 6.9, 2.4 Hz, 2H); ^{31}P NMR (162 MHz, $CDCl_3$) δ 23.78, 23.71; ^{13}C NMR (100 MHz, $CDCl_3$) δ 146.22, 146.07, 135.69, 135.67, 129.19, 129.17, 129.03, 128.97, 128.71, 128.69, 128.68, 128.67, 128.48, 128.04, 128.01, 127.98, 127.90, 127.85, 127.83, 127.77, 126.72, 126.66, 118.56, 118.55, 113.94, 67.52, 67.45, 67.34, 56.68, 56.62, 55.18, 55.12, 53.61, 53.54, 53.51, 37.00, 36.94, 36.79, 36.73. HRMS (ESI) m/z calcd for $C_{22}H_{24}NO_3NaP$ $[M+Na]^+$ 404.1391, found 404.1393.

2-(4-Chlorophenyl)ethyl methyl (phenyl)(phenylamino)methylphosphonate (15). Compound **15** was obtained as a mixture of diasteroisomers (ratio: 1:1) according to the General procedure as colorless oil with 74% yield (315 mg, 0.74 mmol); ^{1}H NMR (400 MHz, $CDCl_3$) δ 7.47 – 7.39 (m, 4H), 7.38 – 7.19 (m, 10H), 7.15 – 7.05 (m, 6H), 6.99 (d, *J* = 8.4 Hz, 2H), 6.75 – 6.67 (m, 2H), 6.62 – 6.53 (m, 4H), 4.74 (dd, *J* = 24.3, 1.7 Hz, 2H), 4.24 (qd, *J* = 6.8, 3.9 Hz, 2H), 4.06 (dq, *J* = 10.1, 6.8 Hz, 1H), 3.81 (ddt, *J* = 10.1, 7.5, 6.7 Hz, 1H), 3.68 (d, *J* = 10.7 Hz, 3H), 3.42 (d, *J* = 10.6 Hz, 3H), 2.89 (t, *J* = 6.8 Hz, 2H), 2.78 – 2.68 (m, 2H); ^{31}P NMR (162 MHz, $CDCl_3$) δ 23.88, 23.85; ^{13}C NMR (100 MHz, $CDCl_3$) δ 146.05, 145.90, 135.64, 132.55, 130.34, 130.27, 129.22, 129.20, 128.72, 128.69, 128.08, 128.05, 127.89, 127.83, 127.77, 118.71, 114.01, 114.00, 67.15, 67.08, 67.01, 66.94, 56.70, 53.74, 53.67, 36.28, 36.22, 36.12, 36.04. HRMS (ESI) m/z calcd for $C_{22}H_{23}NO_3NaClP$ $[M+Na]^+$ 438.1002, found 438.1005.

Methyl 2-(4-nitrophenyl)methyl (phenyl)(phenylamino)methylphosphonate (16). Compound **16** was obtained as a mixture of diasteroisomers (ratio: 1:0.7) according to the General procedure as colorless oil with 76% yield (313 mg, 0.76 mmol); ^{1}H NMR (400 MHz, $CDCl_3$) δ 8.07 (ddd, *J* = 11.4, 8.1, 1.3 Hz, 2H), 7.63 – 7.40 (m, 9H), 7.38 – 7.24 (m, 8H), 7.18 – 7.05 (m, 4H), 6.77 – 6.67 (m, 2H), 6.67 – 6.56 (m, 4H), 5.51 (d, *J* = 7.4 Hz, 2H), 5.40 – 5.26 (m, 1H), 5.09 (dd, *J* = 14.9, 7.4 Hz, 1H), 4.92 (d, *J* = 5.8 Hz, 1H), 4.86 (d, *J* = 5.7 Hz, 1H), 3.82 (d, *J* = 10.8 Hz, 3H), 3.50 (d, *J* = 10.7 Hz, 3H); ^{31}P NMR (162 MHz, $CDCl_3$) δ 24.44, 24.38; ^{13}C NMR (100 MHz, $CDCl_3$) δ 146.15, 146.00, 135.66, 135.63, 135.63, 135.53, 132.56, 132.55, 130.34, 130.27, 129.22, 129.19, 128.72, 128.69, 128.07, 128.04, 127.86, 127.80, 127.74, 118.63, 118.60, 113.90, 67.14, 67.07, 66.99, 66.92, 56.63, 56.59, 55.13, 55.09, 53.66, 53.59, 36.29, 36.23, 36.08, 36.02. HRMS (ESI) m/z calcd for $C_{21}H_{21}N_2O_5P$ $[M+H]^+$ 413.1261, found 413.1263.

***N*-(4-methoxylbenzylidene)benzylamine.** The mixture of 4-methoxybenzaldehyde (300 mg, 2.2 mmol), benzylamine (0.24 mL, 0.22 mmol) and anhydrous $MgSO_4$ (1 g) in dry DCM (50 mL) was stirred overnight at room temperature. The mixture was filtered and concentrated under vacuum. The crude product was purified by column chromatography (silica gel; ethyl acetate/hexanes) to afford target product with 99% yield (2.2 mmol, 440 mg) as a colorless oil. ^{1}H NMR (400 MHz, $CDCl_3$) δ 8.33 (s, 1H), 7.74 (d, *J* = 8.8 Hz, 2H),

7.35 (d, *J* = 4.5 Hz, 4H), 7.32 – 7.22 (m, 1H), 6.98 – 6.88 (m, 2H); ^{13}C NMR (100 MHz, $CDCl_3$) δ 161.27, 139.62, 129.83, 129.20, 128.45, 127.96, 126.89, 114.01, 64.96, 55.36. NMR data were in accordance with those reported in the literature [47].
